# Practical Considerations in the Design and Use of Non‐Crystalline Metal–Organic Frameworks

**DOI:** 10.1002/adma.202505579

**Published:** 2025-07-28

**Authors:** Hamidreza Mahdavi, Farnaz Zadehahmadi, Mehran Arzani, Leena Melag, Ashley L. Sutton, Muhammad M. Sadiq, Zongli Xie, Matthew R. Hill, Benny D. Freeman

**Affiliations:** ^1^ Department of Chemical and Biological Engineering Monash University Clayton VIC 3800 Australia; ^2^ CSIRO Manufacturing Private Bag 10 Clayton South VIC 3169 Australia; ^3^ Department of Materials Science and Engineering Monash University Clayton VIC 3800 Australia; ^4^ Department of Chemical Engineering University of Illinois at Chicago 929 W Taylor St Chicago IL 60607 USA; ^5^ CSIRO Mineral Resources Private Bag 10 Clayton South VIC 3169 Australia; ^6^ John J. McKetta Jr. Department of Chemical Engineering The University of Texas at Austin 200 E. Dean Keeton Street Austin TX 78712 USA

**Keywords:** amorphous MOF (a‐MOF), glass MOF (g‐MOF), liquid MOF (l‐MOF), non‐crystalline metal–organic framework (MOF)

## Abstract

The concept of non‐Crystalline Metal–Organic Frameworks (MOFs) is both theoretically exciting and rich in potential applications. Since their conceptual introduction, research in this field has experienced significant growth. This review provides a comprehensive overview of the design, synthesis, and applications of non‐crystalline MOFs, highlighting the current state of the art. It examines the fundamental principles of non‐crystalline MOFs, the various synthetic approaches, and the nature of non‐crystalline MOFs. Additionally, the review outlines their pathway from the laboratory to industrial applications, emphasizing challenges and opportunities for further development.

## Graphical abstract

This review explores the emerging field of non‐crystalline MOFs ‐ amorphous, liquid, and glassy forms ‐ highlighting how they differ from traditional crystalline MOFs. It discusses their unique synthesis strategies, fundamental principles, and diverse applications. The paper also addresses current challenges andindustrial potential, offering insights into future directions and the transformative role of computational tools in their development.

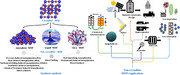

## 1 Introduction

Metal–organic frameworks (MOFs) are an emerging class of porous materials that are composed of metal ions or clusters that are connected by organic linkers to form a 3D network.^[^ ^1^ ^]^ The formation of these frameworks is achieved through coordination bonds, which involve the connection of metal centers into extended, highly ordered structures with long‐range periodicity, which results in crystalline structures. This is typically achieved through the use of multitopic organic linkers, which are molecules with multiple binding sites.^[^ ^2^, ^3^, ^4^ ^]^ MOFs are known for their tunable porosity and exceptionally high surface areas, which can be precisely customized by selecting specific metal ions and organic linkers.^[^ ^5^, ^6^ ^]^ This structural diversity, which is the result of the extensive combination possibilities of inorganic and organic building blocks, enables the development of MOFs with customized pore sizes, shapes, and chemical environments, making them highly versatile materials. The rigid yet porous nature of MOFs allows them to function as robust hosts for immobilizing guest molecules, nanoparticles, or catalytic species within their frameworks.^[^ ^7^ ^]^ Additionally, the chemical tunability of the ligands can be enhanced by the incorporation of functional groups, either during synthesis or through post‐synthetic modification. Furthermore, the thermal and chemical stability of the ligands is based on the composition of the metal nodes, the organic linkers, and the overall framework topology.^[^ ^8^ ^]^ Due to these exceptional characteristics, MOFs have been recognized as a transformative discovery in materials science, providing unique molecular‐level control in material design and exhibiting significant potential across various applications,^[^ ^9^, ^10^ ^]^ including gas adsorption/separation,^[^ ^11^, ^12^, ^13^, ^14^ ^]^ catalysis,^[^ ^15^ ^]^ biocatalysis,^[^ ^16^, ^17^ ^]^ drug delivery,^[^ ^18^ ^]^ and sensing.^[^ ^19^ ^]^

Although MOFs are generally classified as crystalline materials, IUPAC recommendations do not include crystallinity as a prerequisite for their identification or classification. However, the majority of research on MOFs has traditionally focused on their crystallinity.^[^ ^20^, ^21^ ^]^ This emphasis might be because of precise control of chemical functionality afforded by crystalline structures, the limitation of generally accessible tools to adequately depict structural disorder, or a simple lack of awareness of the potential for non‐crystallinity in MOFs.^[^ ^22^ ^]^ However, the search for larger surface area MOFs has led to the development of open and disordered MOF‐based structures that are often more unstable and vulnerable to thermal or mechanical collapse. Such instability presents difficulties for both practical applications and standard methods for dealing with large‐scale morphologies.^[^ ^23^, ^24^, ^25^, ^26^ ^]^ Non‐crystalline MOFs provide a variety of peculiar characteristics that distinguish them from those of crystalline MOFs and conventional materials.^[^ ^20^ ^]^ A paradigm shift from crystalline to non‐crystalline MOFs is underway, driven by several factors. These include the development of novel applications, the ability to induce functionality by introducing defects into structures, and the creation of flexible structures with greater reactivity to external stimuli, along with a growing acceptance of disorder.^[^ ^3^, ^22^, ^27^ ^]^

Unlike their crystalline counterparts, non‐crystalline MOFs are characterized by short‐range order or non‐crystalline networks of metal ions or clusters connected to ligands to create 3D structures with possible porosity. At the same time, non‐crystalline MOFs preserve the basic building blocks, chemical configuration, and connectivity of their crystalline counterparts and structural and compositional variability. However, due to the heterogeneity of their structure and composition, non‐crystalline MOFs are difficult to characterize and reproduce.^[^ ^28^ ^]^ In contrast to crystalline materials, non‐crystalline MOFs possess highly desired bulk properties, such as isotropy and the absence of grain boundaries. They also possess notable mechanical properties, such as flexibility and toughness, that can be utilized in a variety of applications.^[^ ^3^ ^]^ Although non‐crystalline MOFs have already demonstrated applications such as drug delivery, waste storage, and gas separation, their potential in a broader range of applications is only beginning to be explored.^[^ ^29^, ^30^, ^31^ ^]^ The fact that only ≈200 non‐crystalline MOFs are now known lends credence to this assertion.^[^ ^3^ ^]^

There is considerable interest in the design, fabrication, and application of non‐crystalline MOFs due to their exceptional properties. Despite the challenges posed by their heterogeneous composition and structure, the potential applications of non‐crystalline MOFs make them a fascinating and active research topic. Non‐crystalline MOFs have been the subject of numerous brief and general reviews. Bennett and Horike (2018) offered critical insights into the structural design and applications of non‐crystalline MOFs, with particular focus on melt‐quenched glasses.^[^ ^3^ ^]^ Ma and Horike (2022) conducted an analysis of the synthesis, glass formation, and properties of MOFs in amorphous, liquid, and glass states.^[^ ^27^ ^]^ In 2023, Lin et al. made significant contributions to the field by emphasizing the development of direct synthesis methods for MOFs in amorphous state under mild conditions.^[^ ^32^ ^]^ However, a comprehensive article that examines the design, synthesis, and applicability of non‐crystalline MOFs is still lacking. This review distinguishes itself by addressing this gap. Therefore, this review serves to address this gap by investigating the platform approaches to their development. It critically evaluates historical perspectives, research trends, and state‐of‐the‐art design, while also describing their evolution from initial conceptualization to laboratory‐scale implementation through discussions on synthesis methods, properties, and characterization. Furthermore, it investigates the potential pathways for scaling up these materials by investigating the integration of non‐crystalline MOFs into composites and their applications, which is a critical step toward real‐world uses. The review concludes by identifying critical challenges and future research opportunities, thereby establishing itself as a comprehensive resource that complements and extends the existing literature.

## 2 Definition and Chronology of Non‐Crystalline MOFs

### 2.1 Definition

**Figure 1:**
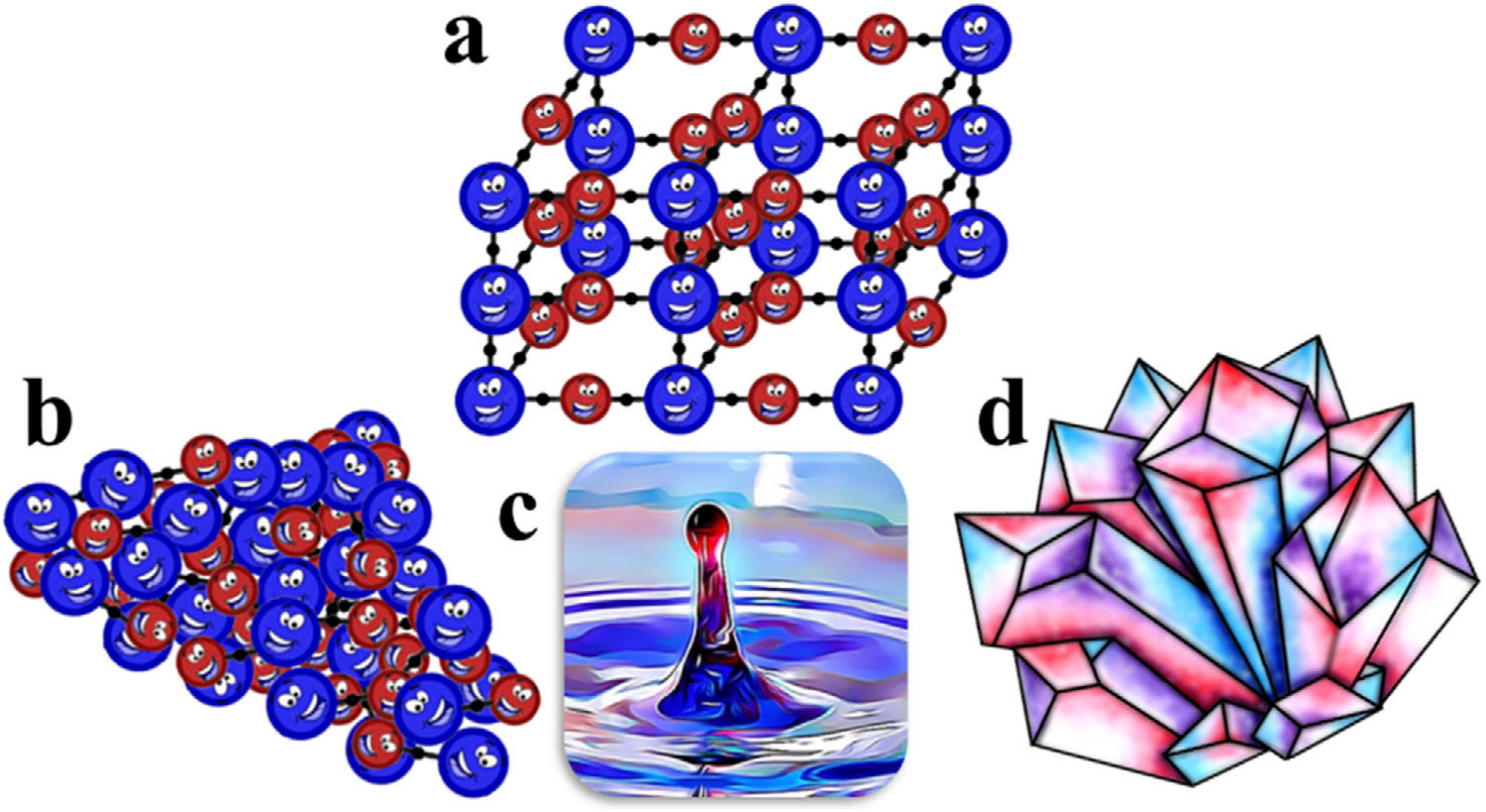
The schematics of a) Crystalline MOFs, b) a‐MOFs, c) l‐MOFs, and d) g‐MOFs.

Non‐crystalline MOFs as an umbrella term for MOFs lacking long‐range order have been classified into three distinct subsets based on their unique structures: amorphous MOFs (a‐MOFs), which are solid, disordered materials typically in powder form; liquid MOFs (l‐MOFs), which exist in a liquid state; and glass MOFs (g‐MOF), which are solid but exhibit a glassy structure. Differences in intermolecular interactions and molecular arrangement make non‐crystalline MOFs structurally different.^[^ ^33^, ^34^ ^]^ The schematics of a‐MOF, l‐MOF, and g‐MOFs are illustrated in **Figure** **1**.

#### 2.1.1 a‐MOFs

a‐MOFs are non‐crystalline MOFs with solid networks, short‐range order (not as other types of non‐crystalline MOFs), and disordered structures that preserve the basic building blocks, chemical configuration, and connectivity of their crystalline counterparts. As a result, they have lower entropy than other types of non‐crystalline MOFs.^[^ ^3^, ^20^, ^35^ ^]^ In a‐MOFs, an increase in atomic mobility while maintaining short‐range order increases atomic entropy. This reduces the pore volume and anisotropy of the microstructure while increasing its density and elastic moduli. Indeed, heterogeneity can improve the mechanical properties of a‐MOFs, such as their flexibility and toughness. As a result, a‐MOFs can trap and delay the transport of guest molecules by collapsing their porous networks in response to stimuli.^[^ ^21^ ^]^ In addition, the a‐MOFs exhibit superior thermal and chemical stabilities, high surface areas, excellent porosities, and the ability to adsorb gases and other small molecules selectively.^[^ ^20^, ^36^ ^]^

Furthermore, a‐MOFs have been shown to possess tunable pore sizes, shapes, and chemical functions typical of crystalline MOFs, as well as the distinctive characteristics of the non‐crystalline domain, such as isotropy, absence of grain boundaries, an abundance of defects and active sites, and flexibility.^[^ ^28^ ^]^ Recent advancements in synthesis and characterization techniques have created new opportunities for the development of these one‐of‐a‐kind materials. The study of a‐MOFs is still in its early stages, and numerous questions remain regarding their fundamental properties and potential applications. However, the exceptional properties of a‐MOFs make them an intriguing research topic for materials scientists and engineers. For instance, a‐MOFs may be more scalable and processable than conventional MOFs due to their amorphous nature, making them desirable for applications like gas storage and separation, catalysis, drug delivery, and sensing.^[^ ^20^ ^]^

#### 2.1.2 l‐MOFs

The l‐MOFs are non‐crystalline MOFs that exist in a liquid state at room temperature or below, possessing a disordered, liquid‐like structure. l‐MOFs maintain a network structure and chemical configuration with short‐range order and retain a significant portion of the porosity of their solid, crystalline counterparts.^[^ ^20^, ^37^ ^]^ The strength of the interactions between the metal nodes and ligands plays a crucial role in the melting of MOFs.^[^ ^38^ ^]^ On the other hand, this bonding is often stronger and more resistant to heat degradation than the covalent bonds that make up the ligands.^[^ ^38^, ^39^ ^]^ Consequently, many have concluded that l‐MOFs cannot be produced because the ligands' thermal decomposition often occurs before melting.^[^ ^22^ ^]^ However, it was found that only certain MOFs could melt before decomposition, a phenomenon associated with metal‐ligand dissociation. These MOFs maintain a stable structure in the liquid state, leading to the discovery of this unique class of non‐crystalline MOFs.^[^ ^37^ ^]^ In contrast to the a‐MOFs and g‐MOFs, l‐MOFs are generated when MOFs are heated to the point where the liquid phase becomes the thermodynamically preferred state.^[^ ^20^ ^]^ The formation of l‐MOFs is followed by an increase in disorder in the metal‐ligand arrangement while short‐range order is maintained, leading to an increase in entropy and structural density. The characteristics of l‐MOFs can be tailored through the selection of specific metal ions and organic compounds during their synthesis. Since research on l‐MOFs is just getting started, many issues about their fundamental properties and future applications remain unanswered. Despite this, l‐MOFs' unusual properties make them an intriguing research topic for materials scientists and technologists. Coatings, sensors, and energy storage are just some of the many potential applications for l‐MOFs because of their ability to be processed and shaped like liquids.^[^ ^37^ ^]^

#### 2.1.3. g‐MOFs

The g‐MOFs are a class of non‐crystalline MOFs with a solid, disordered, glassy structure. g‐MOFs preserve the metal‐ligand arrangement and chemical configuration of their l‐MOF counterparts,^[^ ^20^ ^]^ which primarily act as a precursor to their synthesis by preventing crystallization due to the quenching of l‐MOFs from above the melting temperature (*T*_m_) to below the glass transition temperature (*T*_g_) at a rate fast enough to prevent crystallization.^[^ ^40^ ^]^ The capacity of a supercooled liquid to resist crystallization can be considered an indicator of its glass‐forming ability (GFA).^[^ ^41^ ^]^ The inverse relationship between GFA and liquid fragility, which corresponds to the activation energy of viscosity at *T*_g_, is frequently observed.^[^ ^40^, ^41^ ^]^ Additionally, the ratio *T*_g_/*T*_m_ is used to determine GFA; thus, a liquid with a greater *T*_g_/*T*_m_ has a greater GFA.^[^ ^42^ ^]^ MOFs whose *T*_g_/*T*_m_ ratios exceed the empirically determined limits by more than 2/3 have a high GFA due to the fact that their viscosity increases during the reduced supercooled state.^[^ ^27^, ^40^, ^42^, ^43^ ^]^ Furthermore, g‐MOFs, unlike a‐MOFs, exhibit the same short‐range order as l‐MOFs. This is the outcome of reduced defects in a glassy structure generated from a phase with a high entropy and high molecular mobility.^[^ ^44^ ^]^

In the early phases of research into g‐MOFs, many questions regarding their fundamental properties and potential applications remain unresolved. Still, due to their fascinating characteristics, g‐MOFs present a novel challenge for materials scientists and technologists. One advantage of g‐MOFs is their high stability and structural collapse resistance, allowing them to be utilized in harsh conditions. The g‐MOFs are desirable for various applications such as gas storage, catalysis, and sensing due to their high porosity and diversity of properties.^[^ ^20^, ^43^, ^45^ ^]^

### 2.2 Chronology

**Figure 2:**
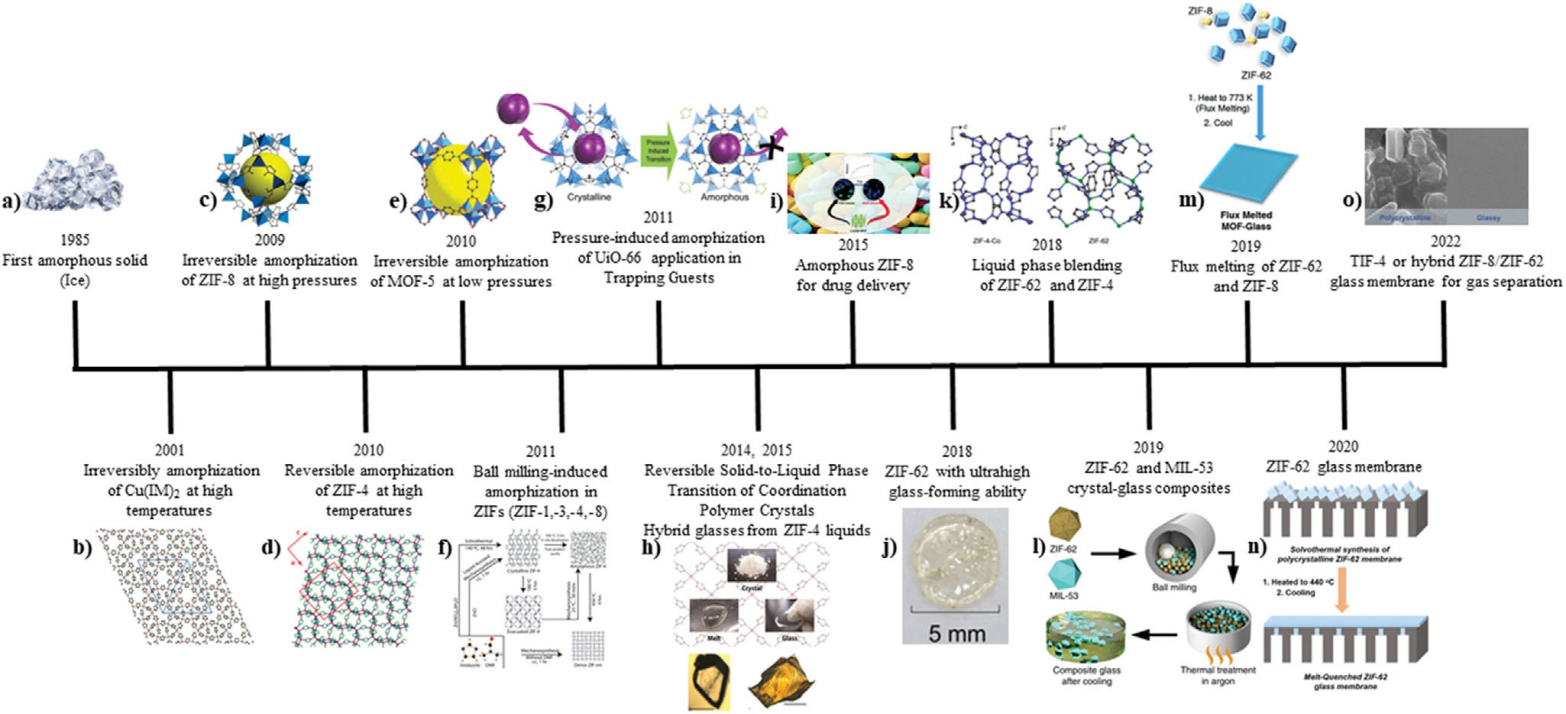
General chronological history of Non‐crystalline MOFs (Image for b): Reproduced with permission.^[^ ^47^ ^]^ Copyright 2001, ACS; Image for c): Reproduced with permission.^[^ ^48^ ^]^ Copyright 2009, ACS; Image for d): Reproduced with permission.^[^ ^5^ ^]^ Copyright 2010, APS; Image for e): Reproduced with permission.^[^ ^49^ ^]^ Copyright 2010, APS; Image for f): Reproduced with permission.^[^ ^50^ ^]^ Copyright 2011, ACS; Image for g): Reproduced with permission.^[^ ^30^ ^]^ Copyright 2011, ACS; Image for h – top one): Reproduced with permission.^[^ ^51^ ^]^ Copyright 2015, ACS; Image for h – bottom one): Reproduced under the terms of the CC‐BY Creative Commons Attribution 4.0 International License.^[^ ^52^ ^]^ Copyright 2015, Springer Nature; Image for i): Reproduced with permission.^[^ ^29^ ^]^ Copyright 2015, RSC; Image for j): Reproduced with permission.^[^ ^43^ ^]^ Copyright 2018; American Association for the Advancement of Science; Image for k): Reproduced under the terms of the CC‐BY Creative Commons Attribution 4.0 International License.^[^ ^53^ ^]^ Copyright 2018, Springer Nature; Image for l): Reproduced under the terms of the CC‐BY Creative Commons Attribution 4.0 International License.^[^ ^54^ ^]^ Copyright 2019, Springer Nature; Image for m): Reproduced under the terms of the CC‐BY Creative Commons Attribution 3.0 Unported License.^[^ ^55^ ^]^ Copyright 2019, RSC; Image for n): Reproduced with permission.^[^ ^56^ ^]^ Copyright 2020, John Wiley and Sons; Image for o): Reproduced with permission.^[^ ^31^ ^]^ Copyright 2022, RSC).

The foundations of non‐crystalline MOFs were established in the late 20th century. The general chronological history of non‐crystalline MOFs was defined by listing significant findings in a timeline and highlighting their significance to the field (**Figure** **2**). The timeline of significant activities in the history of non‐crystalline MOFs will include findings that may not directly concern non‐crystalline MOFs but are crucial to their evolution.^[^ ^3^ ^]^

In 1985, the first amorphous solid was identified when ice underwent an apparent first‐order transition between two non‐crystalline states.^[^ ^46^ ^]^ This led to extensive research into amorphization through pressure and temperature‐induced transitions and amorphization from crystalline to non‐crystalline states. Later, applying the amorphization technique to MOFs led to the discovery of the first non‐crystalline MOFs. In 2001, it was found that certain crystalline polymorphs of copper(II) bisimidazolate (Cu(IM)_2_) were irreversibly amorphized and formed glossy amorphous material using high temperatures (240 °C) under vacuum conditions.^[^ ^47^ ^]^ Zeolitic imidazole framework‐8 (ZIF‐8) was also irreversibly amorphized in 2009 using high pressure of 0.34 GPa.^[^ ^48^ ^]^ In 2010, the first reversible amorphization of a MOF using ZIF‐4 at high temperatures (300 °C) and ambient pressure was recorded. The amorphous form can be restored to its original state at room temperature or transformed into a dense crystalline phase by heating it to 400 °C. The non‐crystalline phase formed on heating was glasslike and capable of plastic flow.^[^ ^5^ ^]^ In 2010, the irreversible amorphization of a MOF‐5 was documented for the first time at low pressure (3.5 MPa) and ambient temperature. This pressure was 100 times lower than is typically needed to amorphize other materials at room temperature.^[^ ^49^ ^]^

In 2011, the first research was published describing the use of ball milling to induce amorphization in MOFs (ZIF‐1,‐3,‐4,‐8). These ZIFs were prepared from various imidazolate ligands by ball milling; once prepared, they were unrecognizable from one another and temperature‐amorphized ZIFs. Higher ball milling frequencies, larger balls, and longer ball milling times all resulted in full amorphization of the ZIFs.^[^ ^50^ ^]^ Through pressure‐induced amorphization, I_2_ has been reported as being captured in ZIF‐8 in 2011. I_2_ is a common harmful by‐product of nuclear energy production, and non‐crystalline MOFs might be used to dispose of it. The amorphization restricted the release of guest species through the deformation of the pore apertures in ZIF‐8 to kinetically trap I_2_ and enhance I_2_ retention. The amorphization did not affect the trapped I_2_ molecules in the ZIF‐8, and the presence of I_2_ molecules did not alter the amorphization conditions.^[^ ^30^ ^]^ In 2014 and 2015, coordination polymer crystals ([Zn(HPO_4_)(H_2_PO_4_)_2_]·2H_2_Im, [Zn(H_2_PO_4_)_2_(HTr)_2_], [Zn_3_(H_2_PO_4_)_6_(H_2_O)_3_]·HBim, [Zn_3_(H_2_PO_4_)_6_(H_2_O)_3_]·H(2‐MeBim), [Zn_3_(H_2_PO_4_)_6_(H_2_O)_3_]·H(2‐ClBim), and [Co_3_(H_2_PO_4_)_6_(H_2_O)_3_]·HBim) and ZIF‐4 underwent melting for the first time to form liquid through a reversible phase transition. This was also cooled to make glasses, as the two new important categories of non‐crystalline MOFs. The coordination bonds were not completely maintained in the liquid state but reformed in the glass state.^[^ ^51^, ^52^ ^]^ In 2015, the encapsulation of calcein as model drug molecules in UiO‐66, followed by ball‐milling to amorphize the framework, was reported. The controlled release of the drug over time in amorphized UiO‐66 was explored. It was determined that UiO‐66 was nontoxic at the concentrations used and that the controlled release of calcein occurred over more than 30 days, as opposed to the 2‐day release time of crystalline UiO‐66.^[^ ^29^ ^]^ In 2018, it was discovered that ZIF‐62 had the highest recorded glass‐forming ability, meaning it has the greatest ability of any known material to prevent crystallization during cooling. After melting, the glass formed from ZIF‐62 had a high viscosity, a very high Poisson's ratio, the greatest *T*_g_/*T*_m_ ratio that has ever been recorded, and a low fragility.^[^ ^43^ ^]^ In 2018, the possibility of combining MOF in its liquid state with another MOF component was investigated. The resultant mixture of ZIF‐62 and ZIF‐4 produced a MOF glass with a domain structure and a single, controllable *T*_g_.^[^ ^53^ ^]^ In 2019, the formation of MOF crystal‐glass composites by dispersing crystalline MOFs within a MOF‐glass matrix was reported. It was discovered that the coordinative bonding and chemical structure of a MIL‐53 crystalline phase is conserved inside the ZIF‐62 glass matrix. In contrast, the composite glass's mechanical properties were improved by interfacial interactions between the closely connected microdomains.^[^ ^54^ ^]^ In 2019, flux melting of MOFs was performed for the first time using the high‐temperature liquid state of one MOF as a solvent for a secondary non‐melting MOF. The liquid state of ZIF‐62, which facilitates the melting of ZIF‐8, provides a way for assessing the *T*_m_ of a non‐melting framework. This increases the porosity available to various guest molecules in the resulting flux‐melted MOF glass.^[^ ^55^ ^]^ In 2020, MOF glass membranes became promising candidates for gas separation membranes because of their high porosity, simplicity of processing, and, most importantly, the possibility of removing the grain boundary that is unavoidable for polycrystalline MOF membranes. The inherent gas‐separation capabilities of a ZIF‐62 MOF glass membrane by melt‐quenching treatment of an in situ solvothermally produced polycrystalline ZIF‐62 MOF membrane on a porous ceramic alumina substrate were evaluated. The resultant membrane exhibited a molecular sieving ability that is far above the Robeson upper bounds.^[^ ^56^ ^]^ In 2022, MOF glass membranes using TIF‐4 with ultra‐high glass forming ability through the melt‐quenching process from polycrystalline membranes and a hybrid ZIF‐8/ZIF‐62 glass membrane through flux melting of MOFs were prepared based on the ingesting advantage of g‐MOFs for gas separation application. The membranes have considerable long‐term stability along with high separation performance, which is beneficial to their industrial use in gas separation.^[^ ^31^, ^44^ ^]^

### 2.3 Bibliometric Analysis

**Figure 3:**
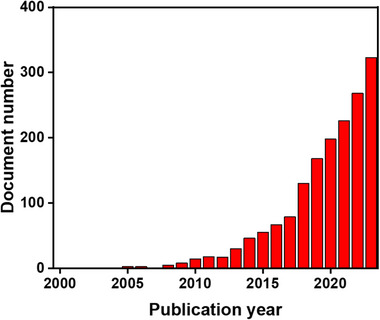
The cumulative number of non‐crystalline MOFs research advancements throughout time (Scopus).

Bibliometric analysis is a quantitative method for analyzing research publications that can shed light on the trends and patterns of research in a specific field.^[^ ^57^ ^]^ In the context of non‐crystalline MOFs, increased focus has been placed on the synthesis, characterization, understanding of the properties, and potential applications of a‐MOFs, l‐MOFs, and g‐MOFs. As demonstrated in **Figure** **3**, the cumulative number of publications each year gives an insight into the non‐crystalline MOFs research advancements over time. A search of the Scopus database for the terms “non‐crystalline MOF”, “amorphous MOF”, “glass MOF”, and “liquid MOF” shows a distinct increase in the number of publications on these subjects over the past two decades. The pioneering research was conducted in the 2000s, sparking significant attention to the topic. The examined bibliometric data indicate that the study of non‐crystalline MOFs is growing significantly and becoming increasingly dynamic, with a broad variety of subjects being investigated by scientists all over the world.

**Figure 4:**
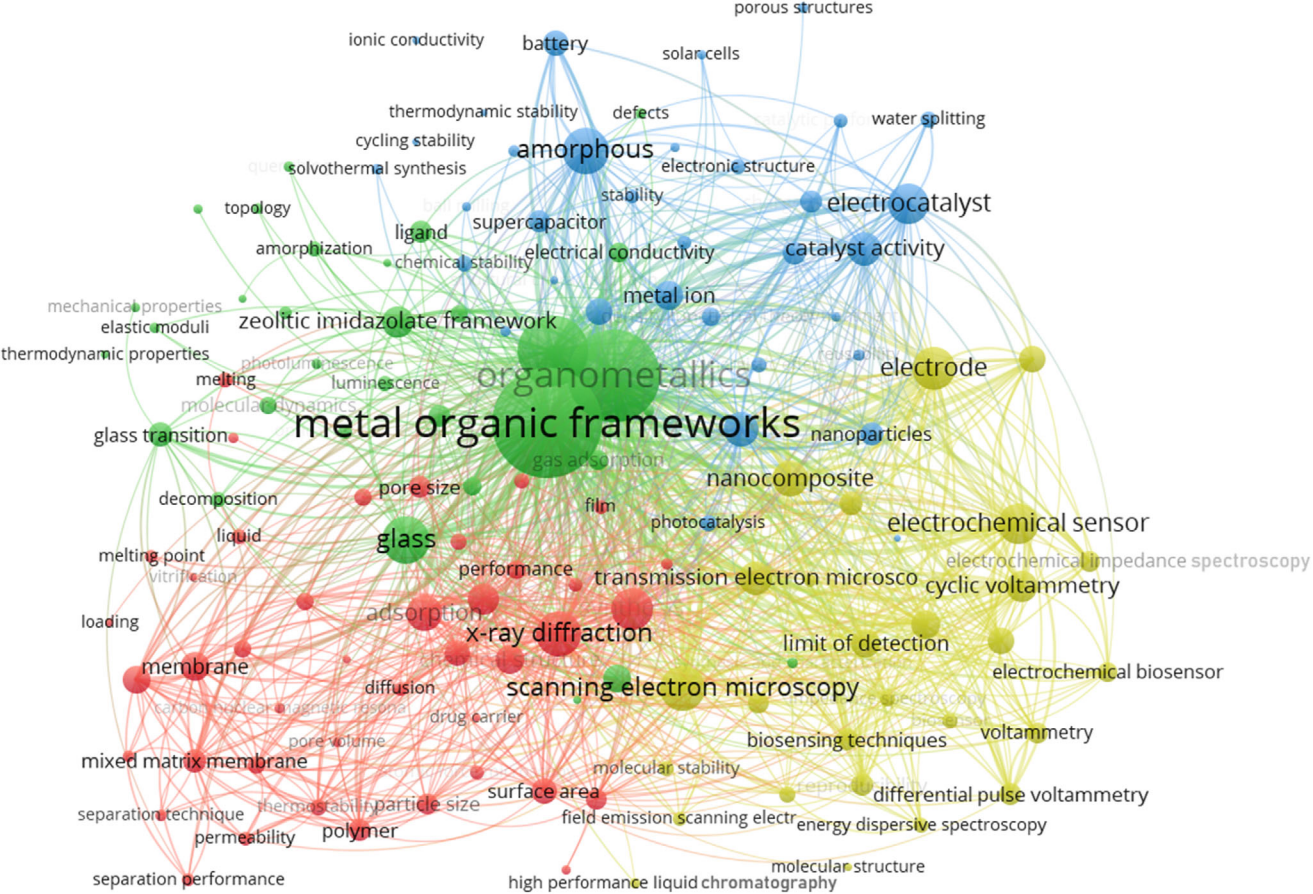
Cluster keywords network in the non‐crystalline MOF field with the most frequent co‐occurrence.

**Figure** **4** illustrates a clustering keywords network developed in the VOSviewer software through co‐occurrence analysis to determine the most frequent keywords in publications associated to the non‐crystalline MOF research. Keywords such as metal–organic frameworks, amorphous, liquid, glass, amorphization, melting, quenching, scanning electron microscopy (SEM), x‐ray diffraction (XRD), thermogravimetric analysis (TGA), transmission electron microscopy (TEM), membrane, mixed matrix membrane (MMM), and gas separation were mapped. By presenting numerous search keywords, the provided material sheds light on such vast issues. As illustrated in Figure 4, the keywords were grouped into three primary clusters using a clustering algorithm based on their co‐occurrence. By clustering the keywords, it is possible to obtain a deeper understanding of the primary and current research trends and patterns in non‐crystalline MOFs and determine potential areas for further investigation. The red‐colored cluster 1, including the keywords metal–organic frameworks, crystalline materials, amorphous, amorphization, pore structure, pore size, chemical stability, and mechanical stability, alludes to the synthesis, characterization, and application of a‐MOFs. Cluster 2, shown by the blue, included keywords such as crystal structure, chemical structure, melting, quenching, liquid, glass, and ZIFs. This cluster focuses primarily on the synthesis and characterization of l‐MOFs and g‐MOFs. The green‐colored Cluster 3 was primarily concerned with the properties and characterization of non‐crystalline MOFs. This cluster includes the keywords SEM, XRD, TGA, TEM, sensor, biosensor, and nanocomposite. In Cluster 4, represented by the color yellow, the keywords membrane, mixed matrix membrane (MMM), gas separation, hydrogen separation, and separation technique were included. This cluster focuses on applications of non‐crystalline MOF.

**Figure 5:**
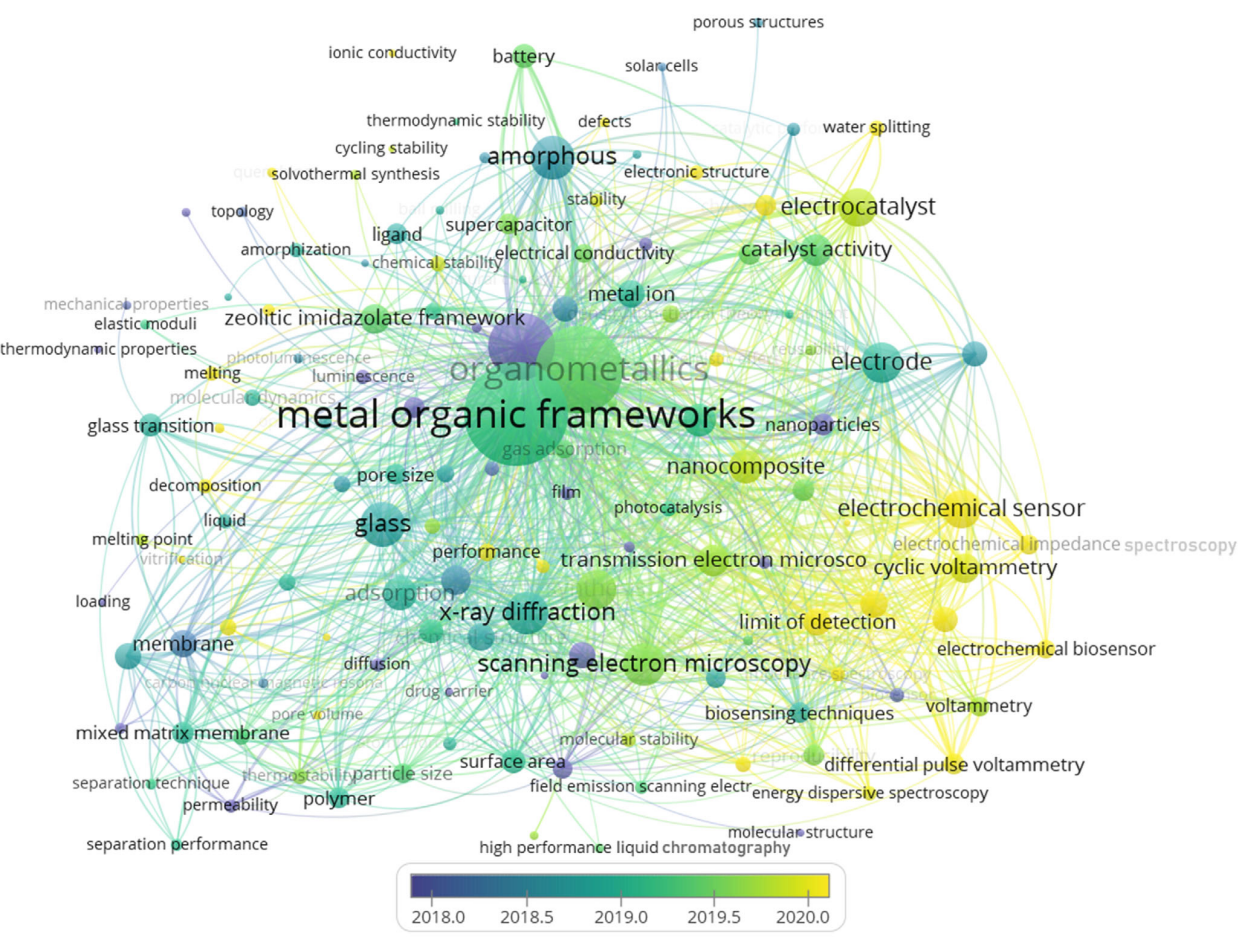
The visualization of excessive keywords in the non‐crystalline MOF field.

Creating a word cloud is one method to visualize the excessive keywords of non‐crystalline MOFs. Word clouds are a basic yet effective method of displaying the prevalence of the most frequently used keywords in a specific field. **Figure** **5** is the visualization of excessive keywords in non‐crystalline MOFs (with the most frequent co‐occurrence). The size of each keyword indicates the frequency with which it appears in publications about non‐crystalline MOFs. In addition, the colors from purple to yellow represent, accordingly, the primary and current focus of the research. The most commonly used keywords in this area are the most prominent words such as MOFs, crystalline materials, amorphous, and glass. Other significant words include membrane, gas separation, and ZIFs, suggesting that these are significant research subjects in the non‐crystalline MOFs field. Smaller words like stability, melting, and liquid indicate related research areas. By visualizing the excessive keywords in this way, it is possible to quickly identify the most current and potential future themes and areas of interest in the field of non‐crystalline MOFs. Based on an analysis of excessive keywords, current research in non‐crystalline MOFs is multidisciplinary, focusing on understanding their synthesis, characterization, and potential applications in a variety of fields. In particular, non‐crystalline MOFs are being studied for their distinctive features and possible applications in gas adsorption and separation, catalysis, energy storage, electrocatalysis, sensing, and drug delivery. Researchers are investigating new synthetic methods for fabricating non‐crystalline MOFs with tailored properties. They employed advanced techniques such as X‐ray diffraction, transmission electron microscopy, and nuclear magnetic resonance spectroscopy to understand their structures better.

## 3 State of Art Development of Non‐Crystalline MOFs

The development of non‐crystalline MOFs is currently in its early stages and necessitates a platform approach to expedite advancements in this field.^[^ ^3^, ^58^ ^]^ A platform approach involves the development of a standardized methodology in several steps for the synthesis, properties evaluation, and characterization of non‐crystalline MOFs. The first step is to establish a standardized method for synthesizing non‐crystalline MOFs. The second step is to develop a set of characterization techniques that can be used to identify and investigate the non‐crystalline MOFs. The third step is to evaluate the properties of non‐crystalline MOFs and compare them with those of their crystalline counterparts. Through the implementation of such platform approach, it is possible to expeditiously develop and evaluate a wide range of non‐crystalline MOFs with different properties and applications. This can help to expedite advancements in this field and open up new opportunities for the development of non‐crystalline MOF materials.

### 3.1 Synthesis of Non‐Crystalline MOFs

**Figure 6:**
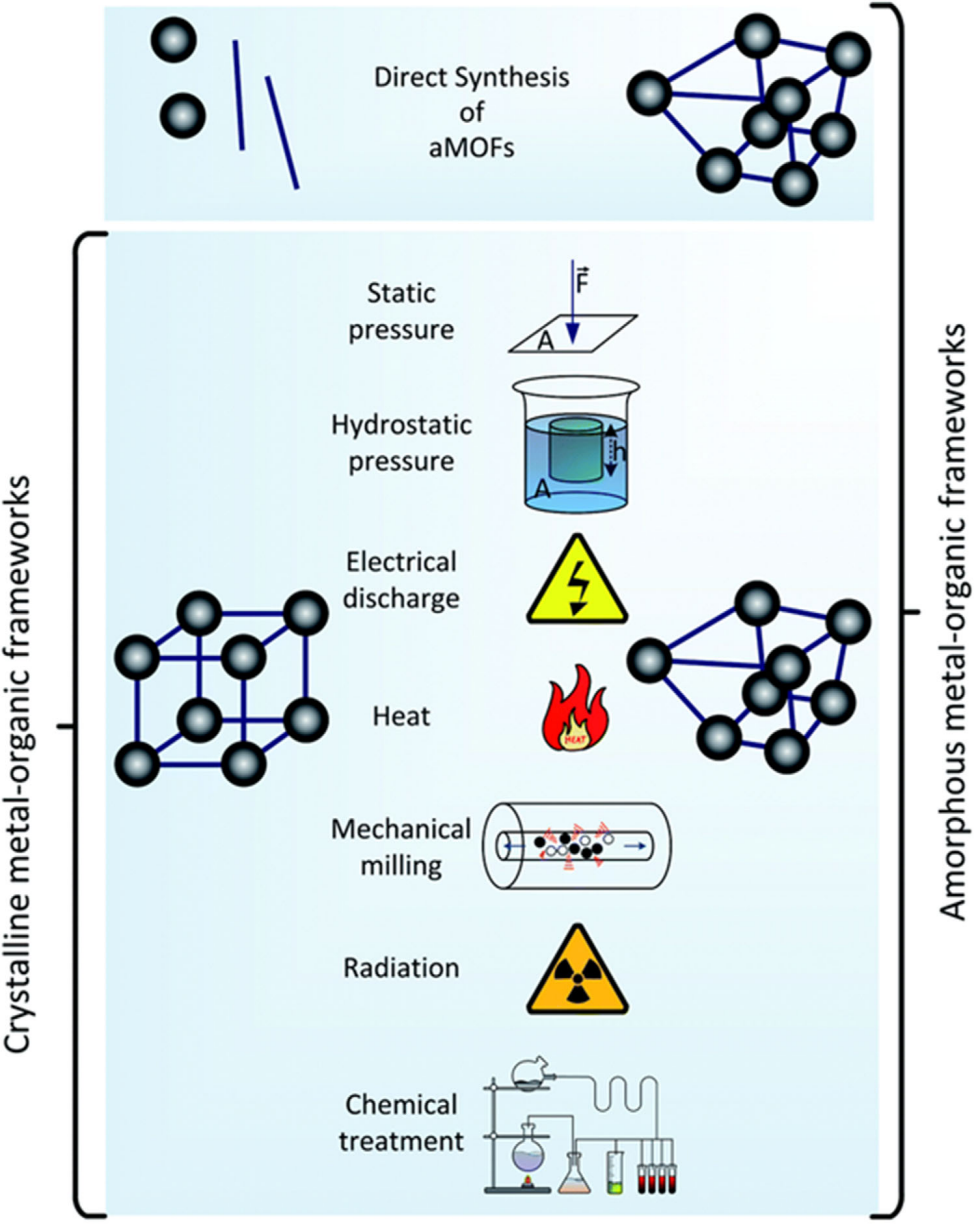
Schematic representation of various a‐MOFs synthesis methods (Reproduced with permission.[20] Copyright 2021, RSC).

**Figure 7:**
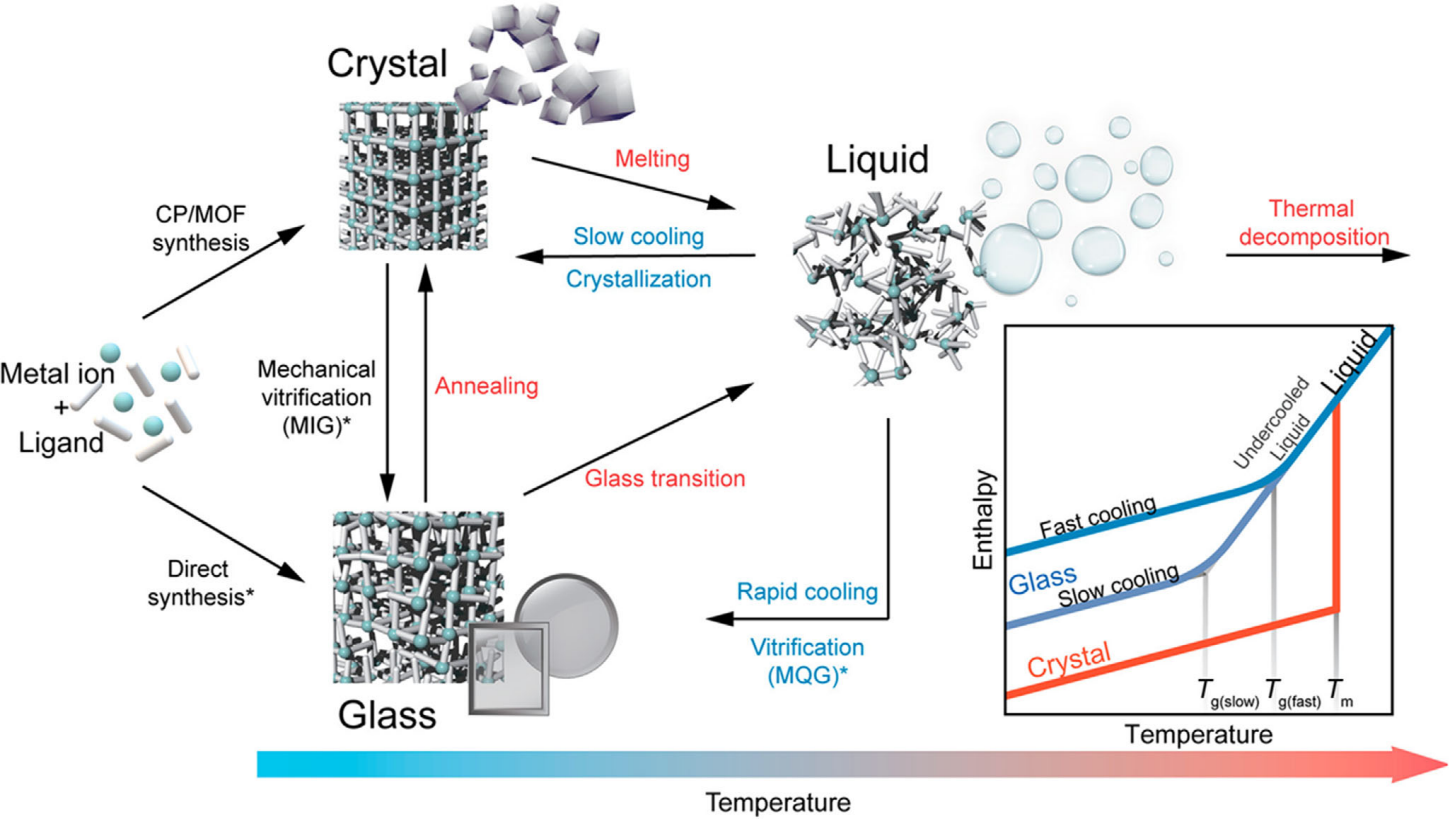
Schematic representation of various l‐MOFs and g‐MOF synthesis methods (Reproduced with permission.^[^ ^27^ ^]^ Copyright 2022, ACS).

The synthesis of non‐crystalline MOFs is an active area of research, with ongoing efforts to develop and optimize new synthesis methods. However, this process is often challenging and requires careful optimization of the synthesis conditions to achieve the desired properties.^[^ ^37^ ^]^ Detailed discussion on the various synthetic methods for a‐MOFs, l‐MOFs, and g‐MOFs is provided in detail below. Additionally, a summary of the key synthesis methods of non‐crystalline MOFs is presented in **Figures** **6** and **7** and Table S1.

#### 3.1.1 a‐MOF Synthesis Methods

##### Pressure‐Induced Amorphization (PIA)

PIA is a phenomenon in which a crystalline material is stressed through the application of static or hydrostatic pressure at temperatures below the *T*_m_ or *T*_g_ range, so it transitions to an amorphous.^[^ ^20^ ^]^ The phenomenon consists of three processes: compaction, where the space is compressed and reduced due to crystal deformation (solidification); compression, where the collapse of partially empty pores occurs; and cataclysmic ductile collapse, where they produce amorphous solids as a result of the continuation of the collapse of the pore structure. PIA has several advantages over other synthesis methods for non‐crystalline MOFs. First, it does not require the use of solvents or other chemical additives, which simplifies the synthesis process and reduces the environmental impact. Second, it can be used to transform existing crystalline MOFs into a‐MOFs, which provides a convenient way to obtain non‐crystalline MOFs with similar chemical compositions and structures to their crystalline counterparts. Finally, PIA can induce unique structural and chemical changes in MOFs that cannot be achieved by other synthesis methods, which can lead to the discovery of new properties and applications for these materials.^[^ ^48^ ^]^ However, the PIA method also has some limitations. The high pressure required for amorphization may induce chemical reactions or structural changes that affect the properties of the resulting non‐crystalline MOF material. Furthermore, the amorphization process may not be complete, and residual crystalline regions may be present in the resulting non‐crystalline MOF material.^[^ ^59^, ^60^, ^61^, ^62^, ^63^, ^64^, ^65^ ^]^

##### Heat‐Induced Amorphization (HIA)

HIA is a sudden change of crystalline material to amorphous due to the *T*_g_. When a crystalline material is given a *T*_g_, it loses the framework pores' solvent. Then the structure collapses, and the more heated, the lower the amorphization temperature. The key factor of this method is the solvent's quick removal, which leads to material collapse for meniscus's liquid‐gas internal forces. Consequently, amorphization temperature will decrease when the heating rate decreases.^[^ ^20^ ^]^

HIA has some advantages over other synthesis methods for non‐crystalline MOFs. Similar to PIA, this method does not require solvents or other chemical additives, simplifying synthesis and decreasing environmental impact. Second, HIA can generate structural and chemical changes in MOFs that standard synthesis methods cannot, revealing new properties and uses. However, the HIA method also has some limitations. The high temperature required for amorphization may induce chemical reactions or structural changes that affect the properties of the resulting non‐crystalline MOF material. Furthermore, the amorphization process may not be complete, and residual crystalline regions may be present in the resulting non‐crystalline MOF material.^[^ ^66^, ^67^ ^]^

##### Mechanical Milling‐Induced Amorphization (MMIA)

Applying milling pressure to the crystalline material beyond its elasticity can change its structure to amorphous due to the destruction of the pores and the occurrence of defects to eliminate its crystallinity. Studies have demonstrated that the milling times for this process typically range from 10 to 30 min for ZIFs, with some experiments extending up to 120 min for other MOFs, depending on the framework and desired amorphization level.^[^ ^68^, ^69^, ^70^ ^]^ Rotational speeds are also crucial, with vibratory mills typically operating at 20–40 Hz and planetary ball mills at ≈300–600 rpm.^[^ ^68^, ^70^, ^71^ ^]^ Mechanical milling phase transformation kinetics is the result of the impact or shearing action of high velocity balls’ energy transforming to the powder. This field has been under many examinations which determined energy transfer is dependent on milling condition including the kind of mill (e.g., vibratory or planetary), the speed of the mill (typically 20–40 Hz for vibratory mills or 300–600 rpm for planetary mills), the ball‐to‐powder weight ratio (often ranging from 4:1 to 10:1), whether the mill is wet or dry, and the length of the milling (variable often between 10–120 min).^[^ ^68^, ^69^, ^70^, ^71^ ^]^ Amorphization of MOFs during mechanical milling is frequently caused by defects. The rise in free energy as well as the number of problems, have not been thoroughly investigated. It should be emphasized that mechanical milling is the most efficient method for producing a broad variety of materials. Its capacity to produce a variety of equilibrium and non‐equilibrium phases that are difficult to produce traditionally makes it a potential technique for solid‐state processing.^[^ ^20^ ^]^

MMIA has several advantages over other synthesis methods for non‐crystalline MOFs. First, it is a simple method that can be easily performed in a ball mill without the need for sophisticated equipment or high‐pressure/temperature conditions. Second, it can be used to transform existing crystalline MOFs into non‐crystalline MOFs, which provides a convenient way to obtain non‐crystalline MOFs with similar chemical compositions and structures to their crystalline counterparts. However, the MMIA method also has some limitations. The high‐energy mechanical milling process may induce chemical reactions or structural changes that affect the properties of the resulting non‐crystalline MOF material. Furthermore, the amorphization process may not be complete, and residual crystalline regions may be present in the resulting non‐crystalline MOF material.^[^ ^69^, ^72^, ^73^, ^74^ ^]^

##### Irradiation‐Induced Amorphization

Irradiation‐induced amorphization is another method for the synthesis of non‐crystalline materials, including MOFs. This method involves exposing crystalline materials to ionizing radiation, such as high‐energy electrons or gamma rays, which can induce disorder and amorphization in the material structure. The mechanism of irradiation‐induced amorphization is not well understood, but it is thought to involve the displacement of atoms from their lattice sites by the high‐energy radiation, which leads to the formation of defects and disorder in the material structure. The degree of amorphization can be controlled by varying the radiation dose and energy, as well as the temperature and pressure conditions during irradiation. The amorphous phase can arise as the crystalline material being subjected to kinetic limitations by irradiation. The rate‐limiting stage of amorphization is the buildup of significant radiation damage in the lattice of the framework. Atomic parameter displacement, a decrease in diffraction intensity and resolution, and site‐specific structural damage are all effects of radiation on crystals. If the framework is not sufficiently robust, local structural damage is accumulated, resulting in long‐range disorder and amorphization.^[^ ^20^, ^75^ ^]^ There are two processes in this phenomenon: the primary process, where the bond is broken due to electron irradiation with cristae; and the secondary process, for emergence of free radicals that can react chemically, which causes damage. One advantage of irradiation‐induced amorphization is that this method can also be used to induce amorphization in a wide range of materials, including those that are difficult to amorphize by other methods. However, there are also some challenges associated with irradiation‐induced amorphization. One major limitation is the potential damage to the material structure and properties caused by the high‐energy radiation. This can result in changes to the chemical and physical properties of the material, which can affect its performance in practical applications. Additionally, the control of the amorphization process, such as the degree of amorphization and the resulting morphology, can be difficult to achieve. Several studies have demonstrated the potential of irradiation‐induced amorphization for the synthesis of non‐crystalline MOFs.^[^ ^76^ ^]^

##### Chemical Treatment‐Induced Amorphization

This phenomenon is the change of crystalline material to amorphous as a result of chemical treatment due to solvents, environmental gases, and free ligands, which attack the free space of the framework. The process of chemical treatment‐induced amorphization can vary depending on the specific chemical agent used and the conditions of the treatment.^[^ ^77^, ^78^ ^]^ In general, the MOF material is dissolved in a suitable solvent or acid to break down the crystal structure and form a disordered solution. The solution is then subjected to a recrystallization process, such as precipitation or spray‐drying, which results in the formation of an a‐MOF material. This occurs in various ways, including coordinated solvent removal ^[^ ^79^ ^]^ water‐induced amorphization in the atmosphere,^[^ ^80^ ^]^ ligand competition, and loading‐induced.^[^ ^81^ ^]^ One advantage of chemical treatment‐induced amorphization is that it can be a relatively simple and inexpensive method that does not require specialized equipment. Additionally, the degree of amorphization can be controlled by varying the concentration of the chemical agent and the conditions of the recrystallization process.^[^ ^82^ ^]^ However, there are also some challenges associated with chemical treatment‐induced amorphization. One limitation is the potential loss of crystalline order and the formation of impurities during the dissolution and recrystallization process. This can affect the chemical and physical properties of the material, which can in turn affect its performance in practical applications.^[^ ^20^ ^]^

##### Direct Synthesis

a‐MOFs can be created directly, meaning without the use of a template or any particular crystalline seeds. This procedure involves dissolving the MOF precursors (i.e., metal ions and organic linkers) in a suitable solvent and mixing them to produce disordered a‐MOF. Caruso et. al comprehensively described the synthetic methods for the amorphous coordination polymers (a‐CP) and MOFs recently.^[^ ^32^ ^]^ Direct synthesis of a‐MOFs is often accomplished by using certain reaction conditions that stop the development of long‐range crystalline order in the final product. Use of a solvent that encourages disorder and inhibits the development of crystalline nuclei is a significant factor. Solvents with high boiling points or solvents that interact aggressively with MOF precursors may be used to accomplish this. The utilization of reaction conditions that encourage quick and uniform nucleation of the MOF precursors is another crucial element. High temperatures, high pressures, or quick mixing of the reactants may accomplish this. a‐MOF synthesis may also be aided by the use of precursors with flexible or sterically hindered structures.^[^ ^32^ ^]^ Due to their conformational flexibility or steric hindrance, which may inhibit the production of crystalline nuclei, these precursors are less likely to form ordered structures.^[^ ^83^, ^84^ ^]^

Numerous methods, including solvothermal synthesis,^[^ ^85^ ^]^ microwave‐assisted synthesis,^[^ ^86^ ^]^ and sonochemical synthesis,^[^ ^87^ ^]^ can be used to carry out this procedure. Direct synthesis of a‐MOFs has many benefits, including better control over the morphology and porosity of the finished product. A‐MOF synthesis may also be more challenging to reproduce since there is no crystalline order, which can lead to differences in characteristics across batches.^[^ ^88^, ^89^ ^]^However, to further improve control in direct synthesis of a‐MOFs and a‐CPs, recently, a basic vapor diffusion approach has been developed which was inspired by the Stöber method. This approach involves the gradual diffusion of triethylamine vapour into a solution of organic ligands and metal salts, which carefully controls the deprotonation process. This approach prevents rapid crystallisation by slowing deprotonation, hence promoting the development of amorphous structures. This careful control of growth kinetics enables the production of amorphous colloids that are monodisperse and also enables the development of complex structures such as core‐shell and shell‐egg. The potential applications of a‐MOFs are expanded by the approach's versatility, which is demonstrated by its ability to function effectively with a wide range of metal ions and ligands.^[^ ^90^ ^]^

#### 3.1.2 l‐MOF Synthesis Methods

##### Direct Melting

l‐MOF is made by the melting of MOFs. Typically, strong coordinative interactions between the metal ions and ligands in a MOF structure require greater energy to break than the covalent bonding within the organic ligands. Accordingly, thermal decomposition of the organic component usually occurs before melting. In the process, the metal ions and ligands must weaken and separate, so the *T*_m_ of MOFs depends on their interaction. MOF structures are comprised of covalently bound ligands and coordinative metal‐ligand bonds. Typically, coordinative bonds are weaker than the covalent bonds and dissociate first under thermal stress. MOFs that can be melted into a liquid need a *T*_m_ higher than the decomposition temperature. However, decomposition before melting is not uncommon, so they decompose before melting. In the review paper reported by Fonseca et. al, a comprehensive description of the synthesis conditions for l‐MOFs by melting prepared MOFs is presented.^[^ ^20^ ^]^ Direct melting removes the need for solvents, which can be costly and environmentally hazardous. Additionally, this removes the need for solvent extraction and later disposal procedures. However, direct melting is only capable of producing l‐MOFs from a select number of MOF families. Due to this limitation, this method may only be suitable for certain applications. In addition, the morphology and structure of l‐MOF produced by direct melting can be less well‐defined. This can result in variable properties and performance characteristics.^[^ ^22^, ^37^, ^91^ ^]^

#### 3.1.3 g‐MOF Synthesis Methods

##### Melt Quenching Amorphization

This method, which is the common method for g‐MOF synthesis, involves melting the crystalline MOF material and then rapidly quenching it to obtain an amorphous solid. This is done to avoid crystallization, maintaining the disordered arrangement and the organic and inorganic building units of l‐MOFs. As a result, MOF glasses protect the integrity of the l‐MOFs' inorganic and organic building blocks.^[^ ^20^ ^]^ The process can be carried out using various methods, such as flame melting, laser melting, or arc melting.^[^ ^84^ ^]^ In this process, a MOF is heated above the *T*_m_, providing a melt state. This melt state is then cooled faster than the rate of crystallization. At *T*_g_, (where *T*_g_ is 2/3 *T*_m_), MOF glasses undergo a supercooled transition. Throughout the decreased supercooled stage, they become progressively viscous, inhibiting crystallization. It is demonstrated that MOFs can behave like glass.^[^ ^20^ ^]^ The advantage of this method is that it is a simple and scalable process, and it can be used to synthesize MOF glasses with high purity.^[^ ^84^ ^]^ However, melt quenching has only been applied to a stable liquid phase of MOF crystals.^[^ ^3^, ^84^ ^]^

Recent studies have proposed innovative synthesis strategies that have greatly advanced the g‐MOFs field. A major development is the incorporation of ionic liquids (ILs) into azolate‐based MOFs to decrease their *T*_m_, thereby facilitating melt processing and following glass production. For example, loading ZIF‐8 with [EMIM][TFSI] reduces its *T*_m_ below its decomposition limit, which facilitates the formation of a molten state that can be rapidly cooled to a glassy state. This technique stabilises bonds during fusion through electrostatic and hydrogen interactions with the IL.^[^ ^92^ ^]^ In addition, co‐melting of azolate‐based MOFs with halide salts is another advance that allows for the continuous modification of the glass structure. The halide salts depolymerize the ZIF structure, adjusting the ratio of bridging to non‐bridging imidazolate linkers. This process is similar to the function of alkali modifiers in traditional glasses, and it enables the modification of *T*_g_. The significance of this development is particularly important for MOFs such as ZIF‐8, which typically undergo decomposition before melting.^[^ ^93^ ^]^ Therefore, these strategies expand the range of MOFs that can be converted into glasses, particularly those that are non‐fusible under conventional conditions. Research into new ligand systems also has expanded the scope of g‐MOFs. Aliphatic carboxylate ligands, such as adipate, connected with metals like magnesium or manganese, can result in the formation of carboxylate g‐MOFs. These ligands facilitate the synthesis of melt‐quenched glasses with very low *T*_m_ (284 °C for magnesium‐based glasses and 238 °C for manganese‐based glasses). The flexibility of aliphatic ligands enhances the entropy of fusion, facilitating glass formation with exceptional functional characteristics.^[^ ^94^ ^]^ In addition to these carboxylate‐based advancements, a novel category of glasses that form networks of aluminium alkoxide has been developed. These glasses are synthesised by connecting aluminum‐oxo clusters with alcohol linkers and utilising a dense monodentate alcohol modulator as both a network plasticiser and pore template. This process results in a high degree of porosity, with a surface area of up to 500 m^2^ g^−1^ after activation.^[^ ^95^ ^]^

##### Mechanically Induced Amorphization

The process of melting is a relatively uncommon occurrence that is limited to a small number of families of MOFs.^[^ ^96^ ^]^ As mentioned before, most MOFs undergo thermal decomposition just as they are heated, where *T*_d_ < *T*_m_, meaning that synthesis through melt quenching is impossible.^[^ ^20^ ^]^ Mechanical stimuli can induce a direct transformation from crystal to glass as an alternative approach for non‐melting MOFs, through compressive, shear, and tensile stresses, using various ways such as ball milling.^[^ ^96^ ^]^ The nonthermal direct phase transition is triggered by the mechanical instability of the framework during the process of pressurization. To comprehend the fundamental basis of mechanically induced amorphization, it is necessary to consider the changes in the elastic tensors. These tensors are indicative of the stress/strain responses in particular directions when pressure is applied. The unpressurized stable crystal exhibits positive eigenvalues in its stiffness matrix, which decrease in specific components when subjected to mechanical stress. Upon surpassing a certain threshold of mechanical load, a phase transition is triggered, whereby the elastic tensor falls below the necessary threshold for Born stability. The process of plastic deformation results in the direct transformation of a crystal into a glass state, which is commonly referred to as solid vitrification.^[^ ^27^ ^]^ Mechanically induced amorphization method offers several benefits, such as a high‐throughput method that enables the rapid synthesis of g‐MOFs without the requirement of solvents or elevated temperatures. The ball milling technique facilitates a fast blending of precursor materials, thereby leading to a synthesis process that is characterized by enhanced efficiency. Furthermore, the aforementioned technique can lead to elevated production rates of g‐MOFs as a result of the close mixing of the precursor materials. The utilization of this method facilitates the creation of g‐MOFs that exhibit diverse compositions and characteristics. While this method presents certain advantages, it is important to acknowledge its limitations. Reproducibility can pose a challenge, as it is dependent upon various factors including milling time, milling intensity, and the characteristics of the precursors. Hence, careful optimization of the procedure is imperative to achieve consistent outcomes. Furthermore, it is noteworthy that the ball milling procedure has the potential to yield a wide‐ranging distribution of particle sizes for the g‐MOF particles, thereby exerting an impact on their characteristics. Several studies have demonstrated the potential of mechanically induced amorphization method for the synthesis of g‐MOFs.^[^ ^20^ ^]^

##### Direct Synthesis

In addition to converting crystal to glass, MOFs can be synthesized directly as amorphous materials exhibiting glassy behaviour, which can be considered equivalent to the sol−gel method in conventional silica‐based glasses.^[^ ^97^, ^98^, ^99^ ^]^ Unlike other methods, the glasses in this class are significantly harder to characterize owing to the lack of an exact composition and single‐crystal structure. Therefore, extensive structural characterizations are required to obtain an exact composition and structure to validate the classification, especially the formation of coordination bond‐based networks.^[^ ^27^ ^]^ The versatility of this method is its primary advantage. The direct synthesis approach facilitates the integration of a wider range of MOFs into the formation of g‐MOFs. The absence of a clearly defined crystal structure poses a significant obstacle to acquiring comprehensive structural insights about them. Limitations in understanding the properties of this class of g‐MOFs and its correlation with its structure may arise from this.^[^ ^100^, ^101^ ^]^ To overcome these challenges, an innovative desolvation strategy has been developed for the synthesis of g‐MOF. The formation of amorphous phases is facilitated by the desolvation of discrete metal‐ligand complexes as precursors that are heated in an inert atmosphere. The suppression of coordinated solvents leads to structural rearrangements, which form metastable metal‐ligand networks exhibiting glassy behavior. Unlike conventional thermal and mechanical vitrification methods, which often fail to produce stable g‐MOFs with tunable properties and enhanced processability due to decomposition of MOF carboxylates, this method provides a new pathway to their production. A significant advancement is the ability to form transparent monoliths without grain boundaries, which thereby expands their applicability. This approach not only overcomes the mentioned structural and stability limitations of direct synthesis, but it also improves the formation of g‐MOFs by allowing for the precise control of their properties.^[^ ^102^ ^]^

### 3.2 Properties Evaluation of Non‐Crystalline MOFs

Non‐crystalline MOFs have distinct properties in comparison to their crystalline counterparts, necessitating a comprehensive analysis of their properties. Therefore, the requirement for properties evaluation of non‐crystalline MOFs is essential to understand their performance and potential applications. However, the evaluation of properties for non‐crystalline MOFs using traditional methods is challenging due to the lack of a well‐defined crystal structure. Changing a material's structure from crystalline to non‐crystalline MOFs causes a disordered rearrangement of its atoms, which in turn affects the material's physical, chemical, and mechanical properties.^[^ ^20^ ^]^ As a result, researchers need to use a range of complementary techniques to understand the properties and behavior of these materials from different aspects such as structural, framework, and functional properties. Moreover, the properties of non‐crystalline MOFs have been presented in Table S2.

#### 3.2.1 Structural Properties of Non‐Crystalline MOF

The primary structural properties distinction between the crystalline and non‐crystalline MOFs lies in the degree of order and arrangement, which subsequently influences the diversity of bonding, crystal structure, and morphology features.^[^ ^20^ ^]^ Crystalline MOFs not only exhibit strong and directional bonding, primarily through coordination bonds formed between metal ions and organic ligands, but additionally, the inorganic nodes and organic ligands are arranged in a well‐defined periodic pattern, resulting in a long‐range order, symmetrical crystal structure, and regular morphology.^[^ ^6^, ^103^, ^104^, ^105^ ^]^ On the other hand, non‐crystalline MOFs still maintain coordination interactions between metal ions and ligands, but the distribution of inorganic nodes and organic ligands lacks organization and a repeating pattern.^[^ ^106^ ^]^ This creates a short‐range order arrangement with a higher degree of local order, as evidenced by an increase in voids, defects, surface functional groups, or other structural irregularities. Therefore, the bonding can be more diverse, including weaker bonds such as ionic bonds, van der Waals forces, hydrogen bonding, and π‐π stacking interactions along with semi‐crystalline or amorphous structure, and irregular morphology.^[^ ^32^, ^107^, ^108^, ^109^ ^]^

The bonding, crystal structure, and morphology features of a‐MOFs, l‐MOFs, and g‐MOFs vary as a result of their distinct compositions and synthesis methods. The bonding characteristics observed in a‐MOFs generally arise from a combination of coordination bonds and weaker interactions. The strength and dynamics of bonds in a‐MOFs are generally lower than those in crystalline MOFs.^[^ ^3^, ^20^, ^38^ ^]^ Furthermore, within a‐MOFs, the local configuration of metal ions and ligands exhibits a resemblance to that of a crystalline MOF, albeit lacking periodicity. Consequently, a‐MOFs are characterized by the absence of a distinct crystalline arrangement and display disorder with a more irregular morphology.^[^ ^20^ ^]^ Moreover, the bonding characteristics of l‐MOFs are primarily defined by relatively weak intermolecular forces, such as van der Waals forces and hydrogen bonding. The presence of these weak forces is responsible for maintaining the liquid phase of the MOF.^[^ ^37^ ^]^ In comparison to crystalline or a‐MOFs, l‐MOFs have weaker and more dynamic bonds between metal centers and organic ligands. Moreover, l‐MOFs lack a stable crystalline structure due to their existence in a liquid phase. Consequently, the constituent atoms and molecules within l‐MOFs exhibit a state of mobility and lack a fixed crystal structure with deformable morphology.^[^ ^5^, ^37^, ^101^ ^]^ On the other hand, g‐MOFs exhibit coordination bonds as their primary bonding and maintain their short‐range order structure upon melting. Consequently, they possess the distorted metal‐ligand arrangement inherited from their precursors, thereby preserving the structural characteristics of the inorganic and organic regions of the l‐MOFs. This preservation of features results in a high level of disorder, randomization, absence of long‐range order, and structural heterogeneity in g‐MOFs. In addition, g‐MOFs lack a well‐defined crystal structure and their atoms are randomly arranged, but they exhibit some local structural order along isotropic morphology with no preferred direction and low or negligible grain boundary.^[^ ^20^ ^]^

#### 3.2.2 Framework Properties of Non‐crystalline MOFs

The framework properties of both crystalline and non‐crystalline MOFs are influenced by their respective structural properties, which encompass factors such as pore size and shape, porosity, flexibility and rigidity, and stimuli responsiveness.^[^ ^3^, ^103^ ^]^ Crystalline MOFs possess notable attributes, including the presence of long‐range order, a symmetrical crystal structure, and a regular morphology. These characteristics contribute to the development of distinct and predictable framework features. Therefore, pore sizes and shapes demonstrate a consistent, systematic, and anticipated structure. This structure exhibits a significant amount of interconnectedness, leading to substantial porosity and a large surface area. Nevertheless, such characteristics establish a rigid framework that constrains their ability to adapt and respond to external stimuli.^[^ ^103^, ^110^ ^]^ On the other hand, non‐crystalline MOFs exhibit a short‐range order arrangement, accompanied by a semi‐crystalline or amorphous structure, and an irregular morphology. As a result, their framework features are not well‐defined and more disordered and variable. Consequently, pore sizes and shapes can display a diverse range, manifesting increased variability and disorder. The complex and diverse pore structures commonly lead to reduced porosity and surface area as a consequence of the existence of small voids and interstitial spaces within the disorganized framework. The management or anticipation of these attributes can pose a significant challenge. However, the greater flexibility and lack of order in the structure enable them to exhibit a heightened level of responsiveness to external stimuli.^[^ ^76^, ^111^, ^112^ ^]^

In terms of pore size, shape, and porosity, a‐MOFs exhibit a high degree of structural heterogeneity, which frequently results in a wide range of pore sizes, irregular pore shapes, and typically lower porosity than their crystalline counterparts.^[^ ^29^, ^69^, ^82^, ^113^, ^114^ ^]^ Research suggests that a‐MOFs typically have surface areas that range from 5 to 1000 m^2^ g^−1^.^[^ ^115^, ^116^ ^]^ This wide range demonstrates the complex and diverse pore structures present in the disordered framework, which often lead to a decrease in porosity compared to their crystalline counterparts. On the other hand, l‐MOFs exhibit a lack of a stable pore structure, resulting in continuous variations in pore size and shape. Nevertheless, a certain degree of porosity is still observed.^[^ ^37^ ^]^ Some studies suggest that l‐MOFs may have pore volumes that are comparable to crystalline MOFs.^[^ ^37^ ^]^ However, the experimental challenges associated with characterizing liquid phases result in the rare reporting of surface area values. In addition, g‐MOFs exhibit a higher degree of structural organization, resulting in a more uniform pore size and shape.^[^ ^117^, ^118^ ^]^ Despite this, it is important to note that g‐MOFs typically have less porosity than a‐MOFs.^[^ ^28^ ^]^ Studies show that g‐MOFs preserve some porosity with a surface area of often less than 5 m^2^ g^−1^.^[^ ^52^, ^119^, ^120^ ^]^ This is predominantly due to their highly organized and constrained structural arrangement, which is the result of grain boundary removal.^[^ ^56^, ^97^, ^118^ ^]^

In terms of flexibility and rigidity, and stimuli responsiveness, a‐MOFs are regarded for their high flexibility resulting from the lack of a well‐defined framework and their disordered structure, which allows for structural rearrangement. However, it is important to note that a‐MOFs still retain certain levels of rigidity due to chemical bonding and local structural constraints. This capacity for adaptability can contribute to an increased sensitivity to stimuli.^[^ ^121^ ^]^ On the other hand, l‐MOFs exhibit a lack of a rigid framework and possess the ability to exhibit significant flexibility. Nevertheless, the potential drawback of this adaptability lies in the compromise of both structural integrity and stability. However, it is fascinating that l‐MOFs exhibit promising characteristics in terms of their ability to respond to external stimuli.^[^ ^37^, ^38^ ^]^ In contrast, g‐MOFs exhibit a higher degree of arrangement and rigidity due to their solid‐state glass nature, leading to reduced flexibility. As a result, these factors contribute to enhanced stability and longevity, while simultaneously mitigating their susceptibility to external stimuli.^[^ ^96^ ^]^

#### 3.2.3 Functional Properties of Non‐Crystalline MOFs

Non‐crystalline MOFs have a wide range of fascinating mechanical, thermal, electrical, optical, and magnetic features that make them useful for a variety of applications.^[^ ^20^ ^]^ Consequently, it is necessary to investigate their mechanical, thermal, electrochemical and dielectric, optical, and magnetic features for various applications. Understanding these features facilitates the production and development of non‐crystalline MOFs with desired characteristics, as well as the improvement of non‐crystalline MOFs' performance for specific applications. In addition, it is essential to comprehend the aforementioned properties of non‐crystalline MOFs in order to ensure their stability and durability in various environmental conditions. Examining these features advances the foundational understanding of non‐crystalline MOFs and their behavior at the atomic and molecular levels. This knowledge could lead to the discovery of novel features and applications for non‐crystalline MOFs.^[^ ^3^, ^30^, ^52^, ^85^ ^]^

##### Mechanical Properties

The mechanical properties encompass elasticity, plasticity, strength, and compressibility, which are governed by Young's modulus, shear modulus, bulk modulus, yield strength, and linear compressibility, and are important for optimizing non‐crystalline MOFs to assure their ability to tolerate external forces or stress, temperature variations, and pressure fluctuations.^[^ ^122^ ^]^ Crystalline MOFs possess a well‐defined arrangement and long‐range order with moderately strong coordination bonds, contributing to great mechanical stability. However, non‐crystalline MOFs have a random bonding arrangement and short‐range order structure often with defect sites, resulting in significant variations in their mechanical properties. Furthermore, the synthesis procedure of non‐crystalline MOFs can significantly influence the mechanical properties. Non‐crystalline MOFs may exhibit distinctive characteristics such as increased density and structural integrity, higher flexibility, and ductility due to the increased packing density and defect density in the material, resulting in lower elastic moduli and greater strain at failure.^[^ ^5^, ^20^, ^84^, ^122^, ^123^ ^]^

The a‐MOFs, l‐MOFs, and g‐MOFs exhibit specific mechanical properties depending on their respective structures and preparation methods. a‐MOFs do not have well‐defined crystal structures, which results in their low mechanical strength; however, they possess high elasticity. For example, the HIA can transform the crystalline MOF by changing its orientation to a‐MOF. Further increases in temperature cause the formation of g‐MOF. Additional rises in temperature beyond this point lead thermal decomposition to occur.^[^ ^124^ ^]^ As a result, the density, Young's modulus, and hardness gradually rise from crystalline MOFs to a‐MOFs and then to g‐MOFs.^[^ ^5^, ^84^, ^124^ ^]^ Alternatively, using the PIA method, the amorphization of anisotropic crystalline MOFs is highly dependent on the direction of applied stresses.^[^ ^125^, ^126^ ^]^ When subjected to uniaxial pressure, the structural changes caused by pressure in a single direction result in a denser arrangement compared to hydrostatic pressure, which applies uniform pressure in all directions. The uniaxial pressure‐driven phases exhibit higher density compared to the hydrostatic pressure produced phases.^[^ ^127^ ^]^

l‐MOFs demonstrate high mobility and fluidity with increased density and decreased porosity. Their properties depend on the ionic chemistry and interaction strength between metal cation and organic anion, with their mechanical properties influenced by temperature, pressure, and composition. They show shear thinning behavior, with viscosity decreasing under stress.^[^ ^122^ ^]^ Indeed, the l‐MOF formation can lead to weakened interaction strength between components, which in turn may cause a decrease in resistance to flow, deformation, and thus viscosity.^[^ ^84^, ^122^, ^128^ ^]^ Due to increased density, most l‐MOFs have low porous structure compared to the glass and crystalline phases. As a result, l‐MOFs have a stable liquid phase and are often used to manufacture melt‐quenched g‐MOFs.^[^ ^122^ ^]^

**Figure 8:**
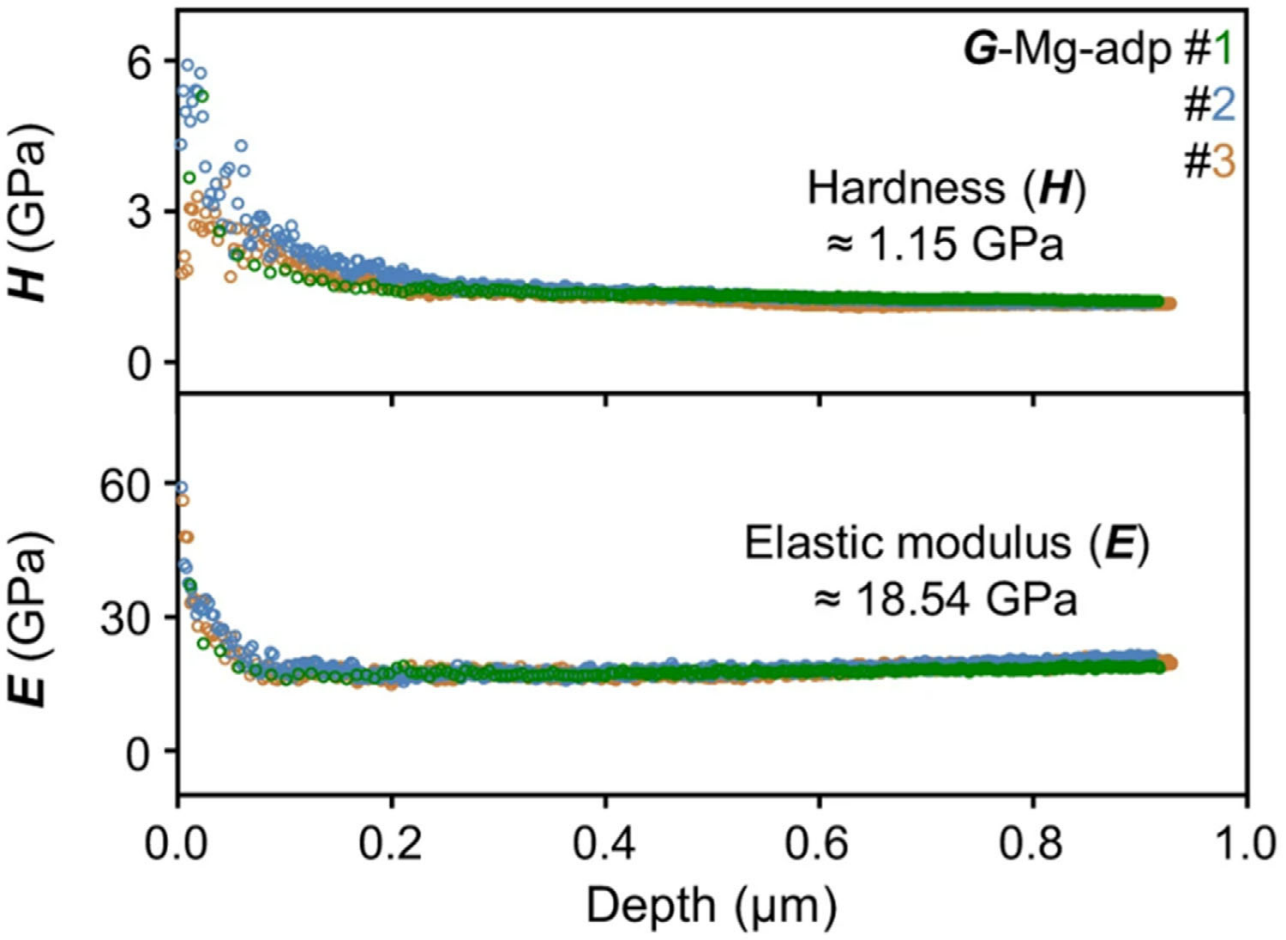
Hardness and elastic modulus of [Mg_4_(adipate)_4_(DMA)(H_2_O)] as a function of nanoindentation depth across three tests (Reproduced under the terms of the CC‐BY Creative Commons Attribution 4.0 International License.^[^ ^94^ ^]^ Copyright 2024, Springer Nature).

The g‐MOFs have a more ordered structure that provides high mechanical strength and low ductility.^[^ ^122^ ^]^ And depending on the structure, composition, defect density, and fluctuations in network rigidity. However, the degree of stiffness varies, making them prone to brittle fracture under stress. For most glass systems, any variations in the crystal chemistry affect the *T*_g_ and mechanical properties of the glasses. Any addition, substitution, or alteration to the MOF structure, especially the use of a bulkier or lengthy linker, alters the packing density and structural flexibility upon cooling and has a direct impact on the *T*_g_ and *T*_m_. For example, in g‐ZIF‐62, higher benzimidazolate (bIm) concentration increased both the *T*_g_ and *T*_m_ due to steric hindrance of bIm while retaining the same ultrahigh *T*_g_/*T*_m_ ratio.^[^ ^20^, ^43^, ^52^, ^84^, ^94^, ^129^, ^130^ ^]^ Moreover, owing to densification of structure, the values of Young's modulus, *E*, and hardness, H, of the g‐MOFs are higher than the crystalline parent phases, and with increasing values of E, the value of H increases.^[^ ^27^, ^131^, ^132^, ^133^ ^]^ Exceptions also exist; for example, g‐ZIF‐62 reveals no ductile fracture, but during the indentation process, it displays abnormal cracking in the vertical direction within the shear band as the weak Zn‐N bonding causes bond breakage. Due to its high Young's modulus (4–6 GPa) and low fracture toughness, K_Ic_, (0.1 MPa·m^[0.5]^), g‐ZIF‐62 has a small fracture surface energy (γ) and its brittle‐to‐ductile transition curve displays anomalous behavior.^[^ ^45^, ^134^, ^135^ ^]^ On the other hand, the mechanical properties of g‐MOFs can be improved. For instance, the nanoindentation studies conducted on carboxylate‐based melt‐quenched g‐MOF, [Mg_4_(adipate)_4_(DMA)(H_2_O)], exhibited higher hardness (*H*), 1.18 GPa, and elastic modulus (*E*), 18.29 GPa (**Figure** **8**),^[^ ^94^ ^]^ higher than any other reported glasses. The strong coordination bonds between carboxylate ligands and metals block the thermal vitrification pathways of these carboxylate‐based MOFs, and the melt quenching process causes structure entanglement and high packing density, resulting in enhancement of their mechanical properties.

On the other hand, the mechanical properties of g‐MOFs can be customised through modifications to their synthesis or use of novel ligands. Cao et al. demonstrated that the co‐melting of ZIFs with heterocycle‐based halide salts, such as benzimidazolium chloride, results in glasses with reduced Vickers hardness (≈0.35 GPa) and fracture initiation resistance (0.12–0.38 N), in contrast to unmodified ZIF glasses (≈0.6 GPa and ≈2 N, respectively). This illustrates that, although densification often enhances mechanical properties, modifications can lead to materials that are more ductile and softer.^[^ ^93^ ^]^ Moreover, recently Kim et al. synthesised G‐Mg‐adp, a carboxylate‐based g‐MOF, from [Mg_4_(adipate)_4_(DMA)(H_2_O). This g‐MOF possesses superior glass‐forming ability (e.g., *T*_g_/*T*_m_ = 0.93 for C‐Mg‐adp with *T*_g_ = 242 °C) and exceptional mechanical properties. Nanoindentation experiments have revealed that the hardness of the g‐MOFs is ≈1.18 ± 0.051 GPa, and the elastic modulus is ≈18.29 ± 0.342 GPa. These values surpass those of previously reported g‐CPs. The high H^[^ ^2^ ^]^/E ratio indicates that the C‐Mg‐adp has enhanced strength and durability as a result of the dense, nonporous structure and strong Mg‐O coordination bonds that are achieved through melt‐quenching. This method significantly improves the mechanical properties by promoting structural entanglement and high packing density.^[^ ^94^ ^]^

##### Thermal Properties

For the purposes of this review, thermal properties include thermal stability, thermal conductivity, thermal diffusivity, thermal expansion, *T*_m_, and *T*_g_ of MOFs. Most thermal properties of non‐metallic materials are the result of phonon transport, which is significantly impeded by structural disorder.^[^ ^136^ ^]^ Crystalline MOFs usually exhibit good thermal stability but have relatively poor thermal conductivity as a result of their porosity.^[^ ^137^, ^138^, ^139^ ^]^ Non‐crystalline materials, due to their structural disorder, tend to exhibit even lower thermal conductivities.^[^ ^140^ ^]^

The three distinct classes of non‐crystalline MOFs (a‐MOF, l‐MOF, g‐MOF) tend to display unique thermal properties. There are reports of a‐MOFs, such as ZIF‐8, that undergo an amorphous phase transition upon ball milling.^[^ ^114^, ^126^, ^135^ ^]^ This ball milling is associated with loss of porosity and an increase in structural disorder and defects. The TGA reveals a steady decrease in thermal stability associated with the time ZIF‐8 is subjected to ball milling. Crystalline ZIF‐8 exhibits a decomposition temperature of 486 °C, which drops to 418 °C after 30 min of ball milling. A further reduction in decomposition temperature to 350 °C is observed after 300 min of ball milling. Indicating the degree of amorphization plays a role in thermal stability.

Amorphization can be carried out with a variety of different processes. Interestingly, Zhou et al. used electrical discharge to induce an amorphous phase change in MOF‐5, Zn_4_O(BDC)_3_ (BDC = 1,4‐benzenedicarboxylate).^[^ ^141^ ^]^ The amorphous phase of MOF‐5 exhibited additional weight loss steps at an earlier stage in the TGA as compared to the crystalline product. These results strongly indicate the amorphous product undergoes a different decomposition mechanism. Notably, the final weight for both crystalline and amorphous products was 39.5% suggesting decomposition products were identical.

l‐MOFs generally lack long‐range order and are comprised of flexible bridging ligands associated with both weak coordination bonds and electrostatic interactions. This allows for their *T*_m_ to be below their decomposition temperature, allowing for fluidity. Cooling a l‐MOF below its *T*_m_ often results in a structural re‐arrangement that results in an amorphous glassy state, commonly known as a g‐MOF.^[^ ^142^ ^]^ Typically, l‐MOFs have high heat capacity, due to the ability of the fluid structure to allow more thermal vibrations and/or rotations. Crystalline ZIF‐4 undergoes a solid‐liquid transition at 856 K and this is associated with a modest increase in heat capacity from 2.2 J g^−1^ K^−1^ for the crystalline phase to 2.8 J g^−1^ K^−1^ for the ZIF liquid.^[^ ^37^ ^]^

**Figure 9:**
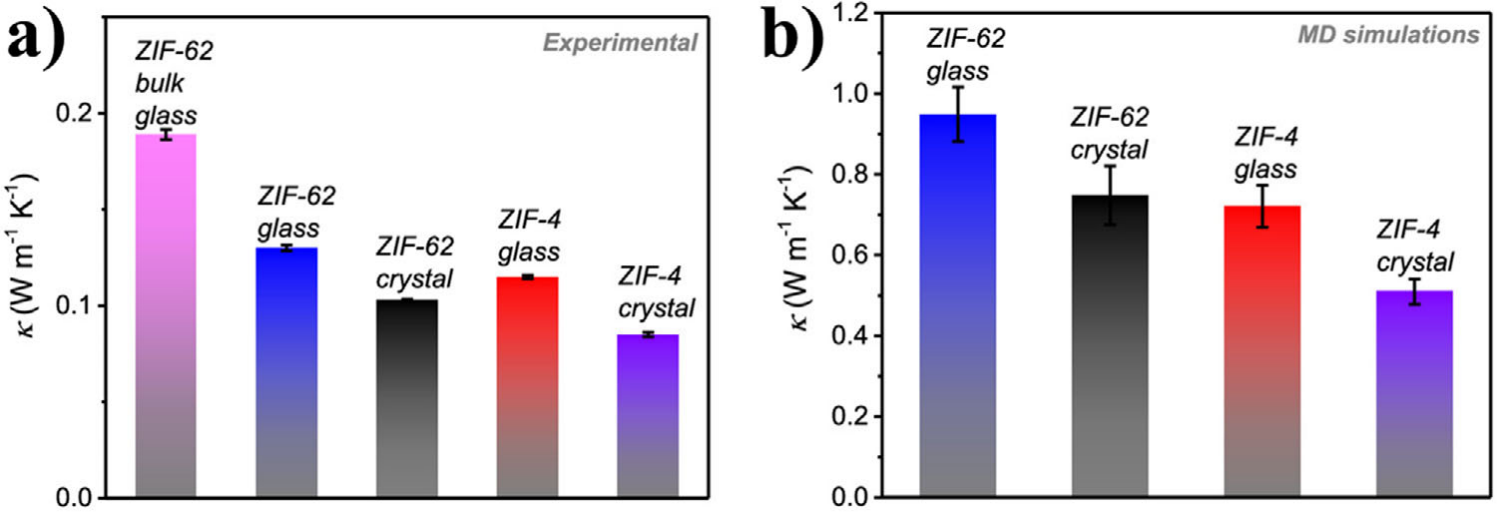
Thermal conductivities of ZIFs, both crystalline and glass, a) measured experimentally, and b) calculated theoretically (Reproduced with permission.^[^ ^145^ ^]^ Copyright 2020, ACS).

The thermal conductivity of glasses is well‐known to be less than that of their corresponding crystalline equivalents. This is due to stronger phonon‐phonon scattering.^[^ ^143^, ^144^ ^]^ In contrast, Sørensen et al. demonstrated that glassy ZIFs such as ZIF‐4, ZIF‐62 exhibit higher thermal conductivity than their crystalline solids (**Figure** **9**a, b).^[^ ^145^ ^]^ With the aid of computational models, they ascribe this unusual behavior to an increased density and reduced flexibility of the organic linkers in the ZIF glasses, a result of the pore collapse and structural distortion upon melting.

##### Electrochemical and Dielectric Properties

The properties of non‐crystalline MOFs discussed in this section of the review include electrical and ionic conductivity as well as dielectric behavior. Crystalline MOFs have generally been considered poor conductors of electrical conductivity, due to a weak interaction between *d‐p* orbitals of metal ions and organic linkers, inhibiting charge‐transport.^[^ ^146^ ^]^ Over the past decade, there have been significant advances with the report of metallic crystalline MOFs.^[^ ^147^ ^]^ Semi‐conducting and conductive MOFs have been developed around several design principles including “through‐bond transport,” “extended conjugation”, “through‐space pathways”, “redox‐hopping”, and “guest‐promoted transport”.^[^ ^148^ ^]^ These design principles can in theory be employed within non‐crystalline MOFs.

**Figure 10:**
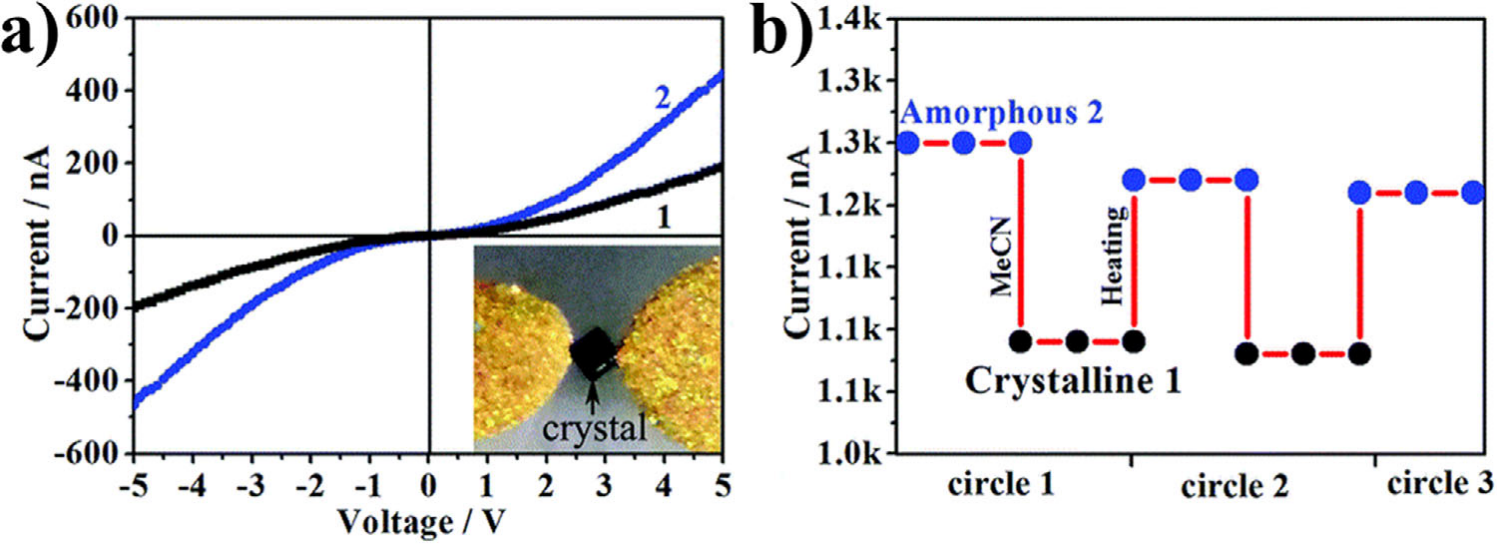
a) *I*–*V* curves of single crystal Cu[Cu(pdt)_2_] (**1)** and amorphous Cu[Cu(pdt)_2_] (**2**), and b) reversible switching of electrical conductivity between (**1**) and (**2**) with stimuli (MeCN & heating) (Reproduced with permission.^[^ ^150^ ^]^ Copyright 2017, RSC).

A common feature of non‐crystalline MOFs is that they classically lack long‐range order, and this is usually associated with the presence of defects and structural disorder. It is well known that such significant disorder or defects in other materials can lead to trapped or localized states.^[^ ^149^ ^]^ In such instances, charge carriers must then thermally hop between localized states, leading to impeded charge transport. There are, however, routes to improve electrical conductivity in non‐crystalline MOFs. One such method is the formation of new through‐bond charge‐transport pathways by the generation of new coordinate bonds. Perhaps, the best understood example of this is with the framework, Cu^I^[Cu^III^(pdt)_2_] (pdt^2−^ = pyrazinedithiolate). Crystalline Cu[Cu(pdt)_2_] undergoes a reversible amorphization (non‐glassy) upon heating to 120 °C for 2 h.^[^ ^150^ ^]^ Surprisingly, the electrical conductivity of amorphous Cu[Cu(pdt)_2_] is 130% higher than crystalline Cu[Cu(pdt)_2_] (**Figure** **10**a,b). Given this process is reversible, this non‐crystalline material has potential for electrical switching behavior. Careful examination by X‐ray photoelectron spectroscopy (XPS) indicated that both the ratio of Cu^I^:Cu^III^ remained identical in both samples and the molecular formula remained unchanged. This strongly indicated a structural change, which was elucidated with Cu K‐edge extended X‐ray absorption fine structure (EXAFS) measurements, which showed the formation of a new Cu─S bond, which can reasonably be expected to facilitate through‐bond charge‐transport.

A very similar example of increasing through‐bond pathways for electrical conductivity in a‐MOFs was reported by Tominaka et al.^[^ ^151^ ^]^ This process started with the crystalline dense insulating MOF, [Cu^I^Cl(ttcH_3_)] (ttcH_3_ = trithiocyanuric acid). Treatment of this crystalline MOF with aqueous ammonia results in a change of chemical composition to Cu^I^_1.8_(ttc)_0.6_(ttcH_3_)_0.4_, associated with a distinct colour change from reddish orange to black. This process is associated with a transition to an a‐MOF‐based material, which has semiconducting behaviour. X‐ray pair distribution function analysis assisted in the assignment of the generation of a new charge‐transport pathway comprised of an inorganic Cu─S─Cu network, which is responsible for the increase in electrical conductivity.

A key aspect for high ion transport is the presence of disordered states, which act as ion carriers. ^[^ ^152^ ^]^ The disordered nature of non‐crystalline MOFs in theory makes them ideal. Horike et al. transformed a proton insulating crystalline MOF, [ImH_2_][Cu(H_2_PO_4_)_2_Cl]·H_2_O (ImH_2_ = protonated imidazole), into a highly proton conductive a‐MOF (10^−2^ S cm^−1^ at 130 °C) in 2014.^[^ ^153^ ^]^ This was achieved by affecting a phase transition with elevated temperature. The phase change was associated with a change in chemical composition associated with the loss of HCl to give [ImH_2_][Cu(H_2_PO_4_)_1.5_(HPO_4_)_0.5_·Cl_0.5_]. This chemical and phase change results in an increase in mobility of the protonated imidazole. This was elucidated with H^2^ NMR solid state spectra at variable temperatures. The crystalline MOF was found to have a broad peak indicative of poor mobility of the deuterated ImH_2_. In contrast the amorphous material under identical conditions exhibited a sharp peak indicative of high mobility, confirming the mechanism for enhanced transport.

Finally, inherent disorder in amorphous materials can lead to a difficulty in dipole alignment, which can give rise to interesting dielectric properties.^[^ ^154^ ^]^ Ultra‐low dielectric constant materials are compelling for future telecommunications, microelectronics, and photonics applications. Several years ago, Wang et al. performed density functional theory (DFT) calculations on a series of a‐ZIFs screening for dielectric properties.^[^ ^155^ ^]^ This study revealed a‐ZIFs with dielectric constants between 1.76 and 2.22. A dielectric constant below 2.5 is considered the threshold for an ultra‐low dielectric material.

##### Optical Properties

The optical properties of both crystalline and non‐crystalline MOFs are of considerable interest. Whilst not a comprehensive list, notable properties include absorption, reflection, diffraction, transmission, scattering, and photon emission. Optical properties are often sensitive and can be influenced by the molecular structure,^[^ ^156^, ^157^ ^]^ chemical composition,^[^ ^94^, ^156^, ^157^, ^158^ ^]^ mode and degree of amorphization,^[^ ^96^, ^156^, ^159^ ^]^ applied pressure and temperature,^[^ ^133^, ^156^ ^]^ as well as the introduction of guest species.^[^ ^129^, ^160^ ^]^

**Figure 11:**
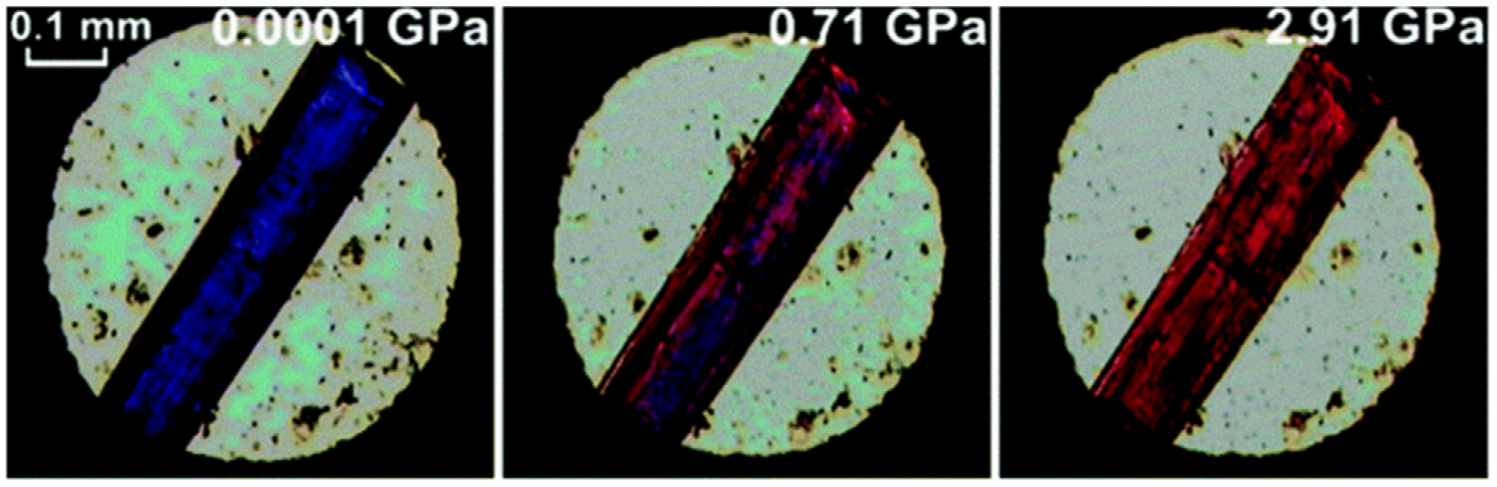
Single crystal of Co_2_(Bdc)_2_Dabco·4DMF·H_2_O undergoing color changing at pressure is increased due to piezochromic behavior (Reproduced with permission.^[^ ^159^ ^]^ Copyright 2017, RSC).

Piezochromism results in a color change upon application of pressure. This effect has been explored for several MOFs,^[^ ^161^, ^162^ ^]^ and is often associated with phase transitions, including those that are pre‐amorphous or amorphous. Andrzejewski et al. examined a piezochromic crystalline MOF, Co_2_(Bdc)_2_Dabco·4DMF·H_2_O (Bdc = 1,4‐benzenedicarboxylate, Dabco = 1,4‐diazabicyclo[2.2.2]octane, DMF = dimethylformamide), at increasing pressure using a range of spectroscopic techniques in addition to X‐ray diffraction.^[^ ^159^ ^]^ At ambient pressures, single crystals of the MOF are vivid blue. At 0.7 GPa, a phase transition occurs in which the MOF becomes purple. This phase transition is associated with a lowering in symmetry. Increasing the pressure further to 1.9 GPa, the crystal undergoes pre‐amorphization associated with a change in color to red (**Figure** **11**). The associated reduction in volume is consistent with twisting and bending of the organic Bdc linker, altering the crystal field of the metal centers. Notably, this process is fully reversible, which could allow these types of materials to act as pressure sensors.

The ease of melting and shaping g‐MOFs lends them to a wide range of potential purposes, including photonics and lighting. Qiao et al. in 2019 reported the production of a transparent g‐MOF using a hot‐press technique with ZIF‐62 without the inclusion of bubble‐like defects.^[^ ^157^ ^]^ Notably, the prepared glass had 90% transparency in both visible and near‐infrared wavelengths, similar to many oxide‐based glasses.

Small changes in chemistry, such as the use of a ligand with differing functional groups, alternate metal species, or stoichiometric changes, can have profound effects on the electronic structure of the system.^[^ ^163^ ^]^ An example of this was reported by Ali et al. who showed glass‐ZIF‐62(Zn) does not exhibit a saturable absorption at 1030 nm, due to its high optical transparency in the near‐infrared region.^[^ ^164^ ^]^ Modifying the composition of ZIF‐62, however, to include Cobalt, to give the non‐crystalline g‐MOF, glass‐ZIF‐62(Zn, Co), results in a material which exhibits a strong non‐linear optical response at 1100 nm. This characteristic suggests glass‐ZIF‐62(Zn, Co) has potential as a near‐infrared optical modulator.

Based on these examples of optical tunability and transparency, later Nozari et al. demonstrated that a glass formed from ZIF‐8 with IL incorporation, a_g_(IL@ZIF‐8‐HT), has unique optical properties. This glass is typically optically transparent. However, it absorbs light in the blue‐to‐green region of the visible spectrum, which results in a brownish‐red colouration. The lack of light scattering and the decrease in absorbance as the UV region approaches indicate a homogeneous glass structure without large‐scale phase separation. This combination of selective absorption and transparency implies that a_g_(IL@ZIF‐8‐HT) may be advantageous for optical applications that need accurate visual qualities or light filtering capability.^[^ ^92^ ^]^

In addition, Zhang et al. recently demonstrated that aluminum alkoxide network‐forming glasses, such as Al‐BHET and Al‐MTBT, possess exceptional optical transparency and fluorescence. Al‐BHET is highly transparent since it is colorless and does not exhibit any absorption in the visible range (400–750 nm). On the other hand, Al‐MTBT is primarily transparent, but it emits blue fluorescence under UV light and exhibits minor absorption near 400 nm. These optical characteristics combined with high porosity (e.g., Al‐MTBT with a BET surface area of 363 m^2^ g^−1^ and Al‐BHET‐TPTO up to 500 m^2^ g^−1^), make these glasses promising candidates for photonics, illumination, and gas separation, where both optical clarity and porosity are critical.^[^ ^95^ ^]^

##### Magnetic Properties

The magnetic behavior of non‐crystalline MOFs remains an area of fascinating exploration. Non‐crystalline MOFs lack a regular repeating atomic structure, which can lead to unique magnetic characteristics. Amorphous phases can disrupt conventional magnetic ordering, resulting in more uncommon behaviors such as spin‐glasses and superparamagnetism.^[^ ^165^ ^]^ The exact magnetic properties of non‐crystalline MOFs are influenced by choice of metal ions, ligands, the coordination environment, and the degree of metal‐metal interactions within the structure.^[^ ^166^, ^167^ ^]^ The transition from a crystalline material to an amorphous counterpart can thus significantly alter the magnetic behaviour. The exact magnetic change is, however, often unpredictable due to structural changes upon amorphization.

**Figure 12:**
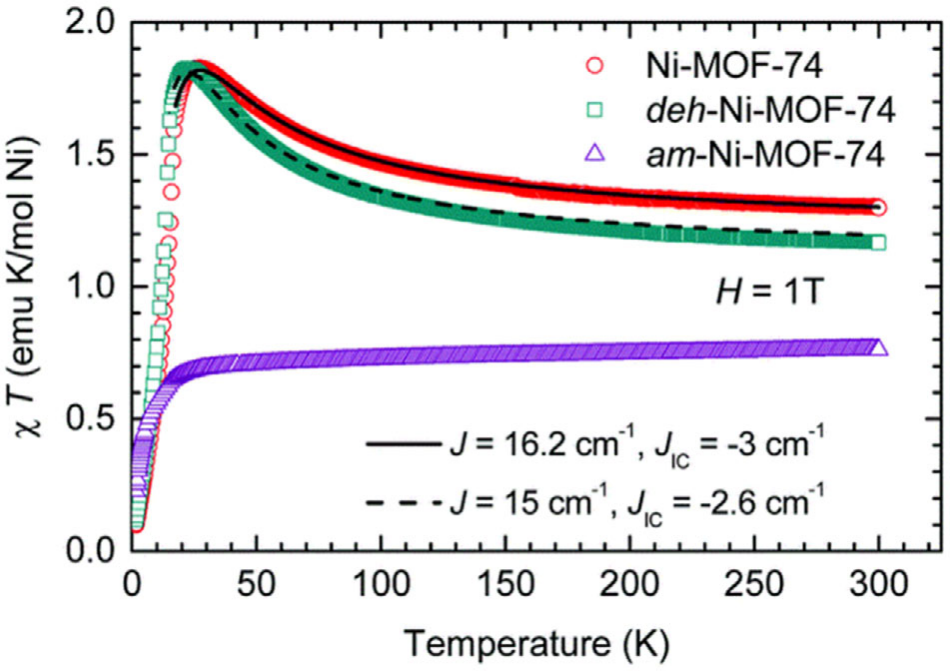
Temperature dependence of χ·T for crystalline (hydrated and dehydrated) and amorphous Ni‐MOF‐74 (Reproduced with permission.^[^ ^168^ ^]^ Copyright 2020, RSC).

Muratović et al. reported an extensive magnetic study on both crystalline Ni‐MOF‐74 and a‐Ni‐MOF‐74 in 2020.^[^ ^168^ ^]^ Briefly, they showed the effective magnetic moment of crystalline Ni‐MOF‐74 to be consistent with Ni(II) in a high‐spin state, S = 1. The magnetic properties were dominated by ferromagnetic exchange (*J* ≈ 16 cm^−1^) between Ni centers along the nickel‐oxo 1D chains. Also present are weaker antiferromagnetic interactions (*J* ≈ −3 cm^−1^) between the adjacent nickel‐oxo 1D chains. Mechanical amorphization of crystalline Ni‐MOF‐74 to a‐Ni‐MOF‐74 results in a significant decrease in bulk magnetization (**Figure** **12**). This can be explained by spectroscopic measurements that indicate a direct change to the coordination sphere. Which results in a spin‐crossover of the Ni(II) from magnetic high spin, S = 1, to non‐magnetic low spin, S = 0. Notably, not all Ni(II) centers transition from high to low spin; only about half. This highlights that structural changes during amorphization processes can have profound effects on magnetic properties.

Transitions from crystalline MOFs to their amorphous counterparts have been capable of increasing spin‐frustration.^[^ ^78^ ^]^ The crystalline MOF, (Me_2_NH_2_)[Co_3_(Me_2_NH)_3_(OH)(SDBA)_3_] (H_2_SDBA = 4,4′‐sulfonyldibenzoic acid), hereafter referred to as Co(II)‐MOF, is comprised of secondary building units that consist of three Co(II) centers arranged in a triangle with *C*_3_ symmetry. Thermal treatment of Co(II)‐MOF under vacuum conditions results in the removal of protonated dimethylamine as well as hydroxide, generating an amorphous neutral MOF. This process is associated with coordination changes to the Co(II) coordination sphere, which alters the magnetic exchange pathway. This results in stronger spin‐frustration than that observed in crystalline Co(II)‐MOF.

### 3.3 Characterization of Non‐Crystalline MOFs

**Figure 13:**
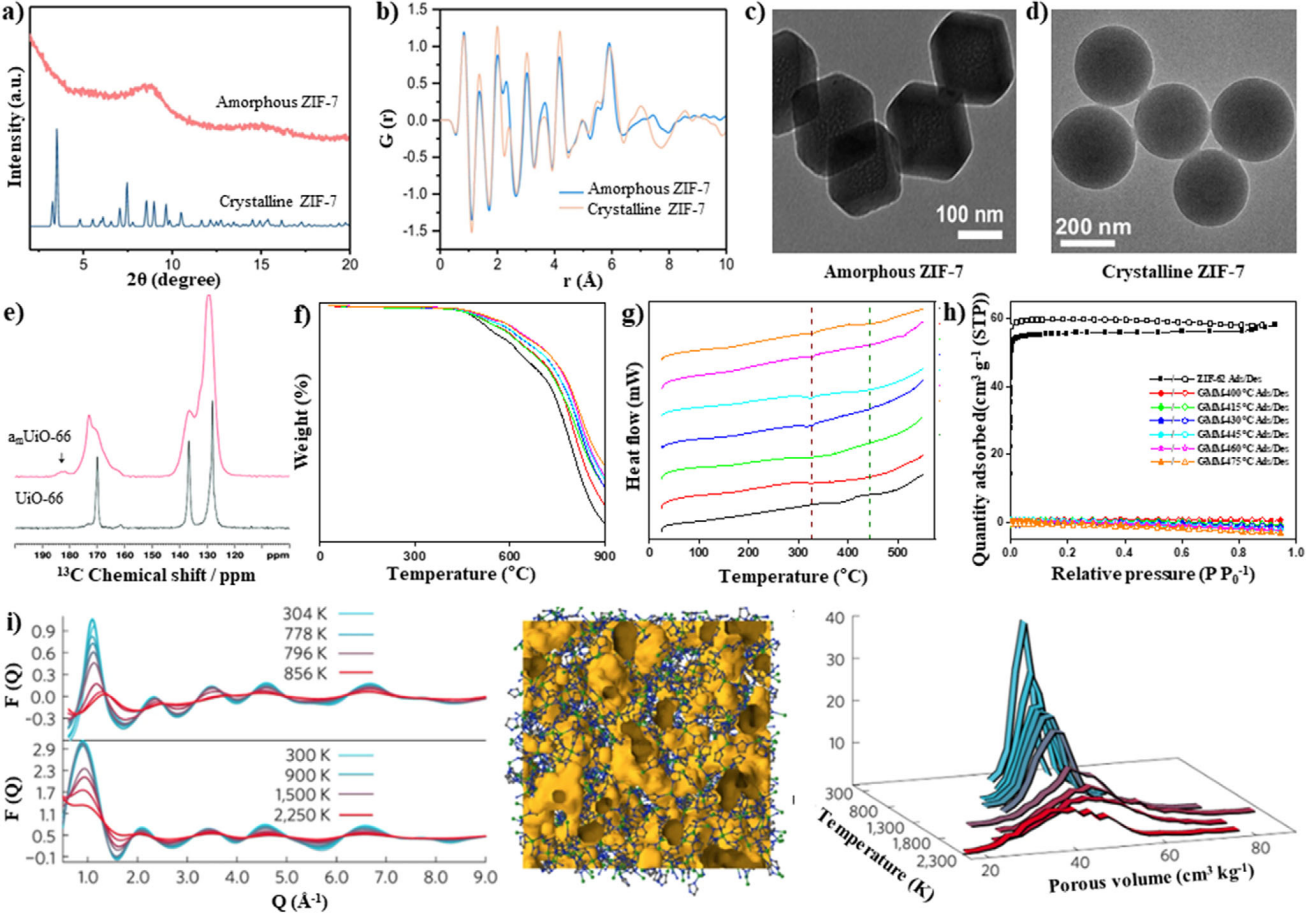
a) PXRD pattern of amorphous and crystalline ZIF‐7; b) PDF analysis of amorphous and crystalline of ZIF‐7;^[^ ^90^ ^]^ TEM images of c) crystalline ZIF‐7,^[^ ^172^ ^]^ and d) amorphous ZIF‐7;^[^ ^90^ ^]^ e)^[^ ^13^ ^]^C NMR of amorphous and crystalline UiO‐66;^[^ ^68^ ^]^ glass and crystalline ZIF‐62 f) TGA, g) DSC, and h) N_2_ gas sorption isotherm;^[^ ^120^ ^]^ i) RMC simulations of ZIF‐4 liquid structure.^[^ ^37^ ^]^ (Image for a‐b): Adapted under terms of CC‐BY Creative Commons Attribution 4.0 International License.^[^ ^90^ ^]^ Copyright 2024, Nature; Image for c): Adapted with permission.^[^ ^172^ ^]^ Copyright 2022, RSC; Image for d): Adapted under terms of CC‐BY Creative Commons Attribution 4.0 International License.^[^ ^90^ ^]^ Copyright 2024, Nature; Image for e): Adapted under terms of the CC‐BY Creative Commons Attribution 3.0 Unported License.^[^ ^68^ ^]^ Copyright 2016, RSC; Image for f‐h): Adapted with permission.^[^ ^120^ ^]^ Copyright 2025, ACS; Image for i): Adapted with permission.^[^ ^37^ ^]^ Copyright 2017, Nature.

Characterizing non‐crystalline MOFs can be challenging due to their absence of the long‐range order exhibited by their crystalline counterparts.^[^ ^169^ ^]^ As presented in Table S3, different techniques can be employed to analyze the structure and configuration of non‐crystalline MOFs, including but not limited to X‐ray scattering, TEM, solid‐state Nuclear magnetic resonance (ssNMR) spectroscopy, and thermal analysis. X‐ray scattering techniques such as XRD pattern, X‐ray absorption fine structure (XAFS), small‐angle X‐ray scattering (SAXS), and wide‐angle X‐ray scattering (WAXS) are viable means to procure insights regarding the structure and morphology of a‐MOFs. XRD patterns of non‐crystalline MOFs typically exhibit broad, diffuse peaks compared to the sharp peaks of crystalline materials, indicating the degree of structural disorder. XAFS, which includes XANES and EXAFS, provides detailed information about the local environment around metal atoms, such as oxidation state and coordination number. SAXS is particularly advantageous for determining the size and shape of nanoparticles or pores within the MOF structure, and WAXS probes atomic‐scale structures, revealing broad features characteristic of amorphous materials.^[^ ^170^ ^]^ The techniques above can furnish data associated with the size and shape of the particles, along with the distribution of the metal and ligand constituents. However, such techniques are often limited to the first or second coordination sphere and cannot give information about the extended structure as highlighted by Bennet et. al.^[^ ^35^, ^97^, ^171^ ^]^ For example, the XRD pattern of a‐ZIF‐7 (**Figure** **13**a) demonstrates broad, diffuse peaks, which confirms the absence of long‐range order, a property that is unique to amorphous materials. In contrast, the XRD pattern of crystalline ZIF‐7 exhibits sharp and strong peaks, which suggest the existence of long‐range order.^[^ ^90^ ^]^

It is noteworthy that the pair distribution function (PDF) analysis is a method that can yield structural insights from disordered materials through the utilization of the entire powder XRD pattern.^[^ ^27^ ^]^ This method allows for the identification of short‐range order and local structural motifs. For instance, the PDF analysis of a‐ZIF‐7 (Figure 13b) displays similar short‐range order to their crystalline counterparts, suggesting the presence of local structural motifs despite the overall disorder.^[^ ^90^ ^]^ The TEM technique has the potential to provide a nanoscale visualization of the morphology of non‐crystalline MOFs. TEM images can provide valuable insights regarding the size, shape, and structure of particles. Additionally, advanced techniques such as scanning TEM (STEM) or high‐resolution TEM (HR‐TEM) can provide atomic‐scale imaging, which can reveal local ordering or defects. As shown in Figures 13c,d, the TEM images of a‐ZIF‐7 demonstrate a monodisperse spherical shape with a size of ≈350 nm.^[^ ^90^ ^]^ Whereas, the TEM images of crystalline ZIF‐7 show hexahedral morphology, with a particle size of ≈200 nm.^[^ ^172^ ^]^ Solid‐state NMR spectroscopy enables the acquisition of insights regarding the local structure of a‐MOFs. The technique, as mentioned earlier, can present insights into the bonding interactions between metal ions and ligands. It can also detect potential defects or structural irregularities by probing specific nuclei such as^[^ ^13^ ^]^C or^[^ ^1^ ^]^H to reveal coordination environments and dynamics.^[^ ^27^ ^]^ As shown in Figure 13e, the highly ordered structure of crystalline UiO‐66 is reflected in the sharp NMR peaks. However, the loss of long‐range order results in a significant peak broadening of all signals after amorphization. Despite this, the primary spectral features continue to exist, which suggests that the local chemical environment remains largely intact.^[^ ^68^ ^]^

The utilization of TGA and differential scanning calorimetry (DSC) as thermal analysis techniques can provide valuable information on the thermal stability and decomposition features of non‐crystalline MOFs. These techniques can give data related to the thermal characteristics of the material, including the initiation of thermal decomposition and the heat of decomposition. TGA measures weight loss to indicate decomposition temperatures and the presence of volatile components, while DSC detects *T*_g_ and *T*_m_ characteristic of amorphous materials. For instance, the TGA data (Figure 13f) demonstrate that the g‐MOFs maintain thermal stability at temperatures ≈400 °C with minimal weight loss. In contrast, the crystalline ZIF‐62 experiences a more significant and sharp weight loss. The enhanced thermal stability of g‐MOFs can be seen by the gradual mass reduction, which is caused by its amorphous nature in contrast to the ordered crystalline framework.^[^ ^120^ ^]^ Moreover, the DSC curve (Figure 13g) of crystalline ZIF‐62 demonstrates that the *T*_g_ and *T*_m_ emerge at 327 and 443 °C, respectively. Therefore, the g‐MOF could not be yielded at temperatures below 443 °C, and the gradual decomposition of ZIF‐62 was observed as T reached 550 °C. As a result, it is expected that g‐MOF fabricated at 445 °C near the *T*_m_ exhibits the most glass‐like properties. This is due to its homogeneous morphology, without exceeding the *T*_m_, which could result in the gradual formation of mesopores and bubbles.^[^ ^120^ ^]^ Gas adsorption studies are performed to characterize porosity and surface area, which are essential for MOF applications. As illustrated in Figure 13h, the N_2_ gas sorption isotherm of crystalline ZIF‐62 exhibits a type I profile with a BET surface area of 58.8 m^2^ g^−1,^ which is typical of microporous materials. However, the g‐MOF membranes do not exhibit N_2_ adsorption due to formation of a dense, non‐porous structure. This contrast is the result of the microporous framework collapsing during thermal treatment of g‐MOF formation.^[^ ^120^ ^]^

Additionally, the reverse Monte Carlo (RMC) simulation technique can be employed to iteratively improve a simulated material model until it accurately corresponds with experimental data. This provides atomistic insights into the disordered structure. Subsequently, the model obtained can be employed for analyzing the structure and configuration of the material under investigation.^[^ ^27^, ^97^ ^]^ The comprehensive understanding of the structure and configuration of non‐crystalline MOFs necessitates the utilization of a combination of various techniques. The utilization of these techniques may offer important data for the purpose of designing and enhancing the efficiency of MOFs across diverse applications. Figure 13i illustrates the use of RMC modelling to X‐ray total scattering data obtained at 856 K to connect the predicted liquid structure of ZIF‐4 with the experimental results. The analysis indicates that the liquid phase maintains significant porosity, exhibiting an internal surface area of 16.2% calculated using a 2.4 Å probe diameter. This persistent porosity comes from connected voids maintained during the melting transition.^[^ ^37^ ^]^

## 4 Non‐Crystalline MOF Composites

**Figure 14:**
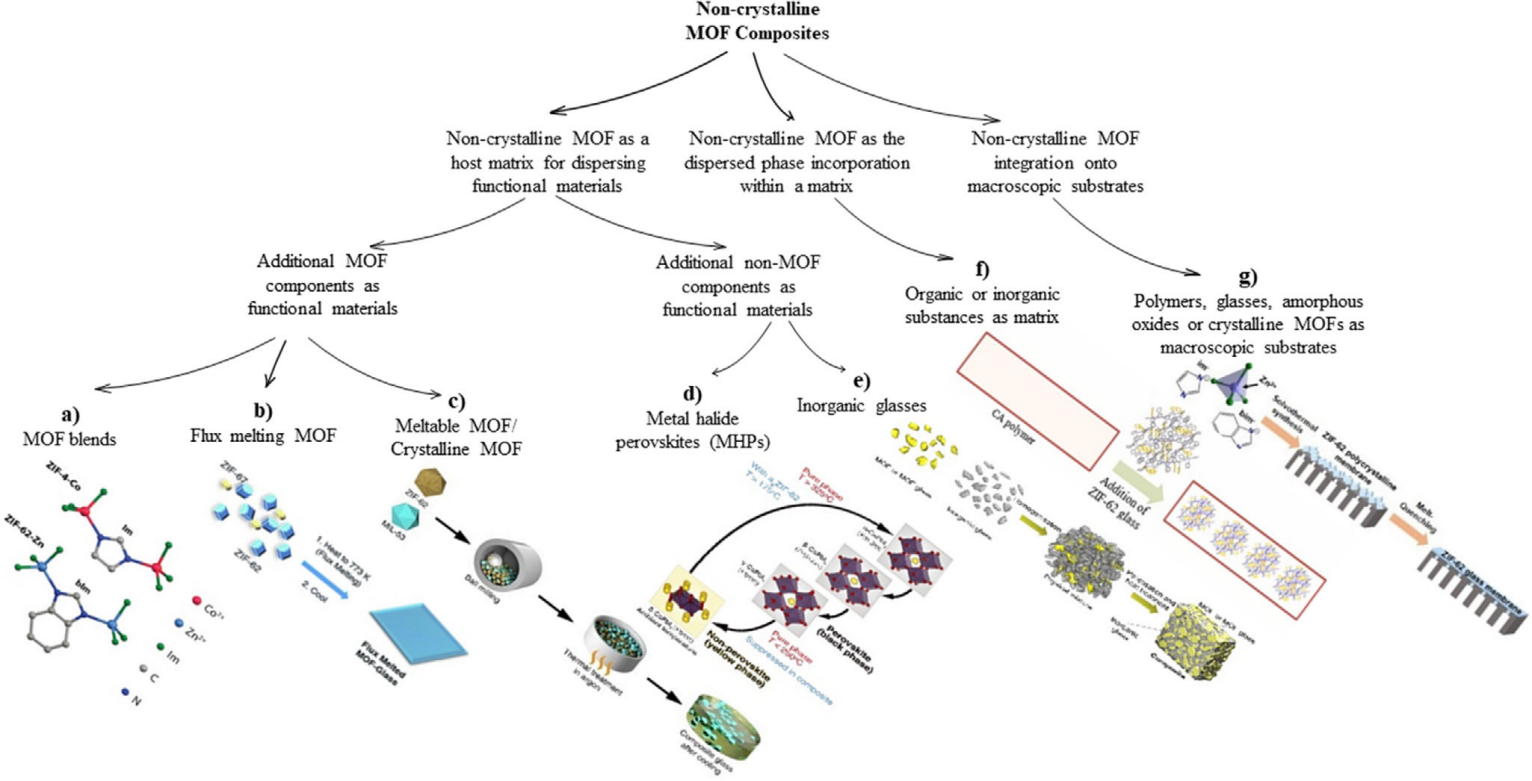
A summary of the major non‐crystalline MOF composites (Image for a): Reproduced with permission,^[^ ^175^ ^]^ Copyright 2018, ACS, Image for b): Reproduced under terms of the CC‐BY Creative Commons Attribution 3.0 Unported License.^[^ ^55^ ^]^ Copyright 2019, RSC, Image for c): Reproduced under terms of the CC‐BY Creative Commons Attribution 4.0 International License.^[^ ^54^ ^]^ Copyright 2019, Springer Nature, Image for d): Reproduced with permission.^[^ ^178^ ^]^ Copyright 2021, The American Association for the Advancement of Science, Image for e): Reproduced under terms of the CC‐BY Creative Commons Attribution 4.0 International License.^[^ ^180^ ^]^ Copyright 2022, John Wiley and Sons, Image for f): Reproduced with permission.^[^ ^184^ ^]^ Copyright 2021, Elsevier, Image for g): Reproduced with permission.^[^ ^56^ ^]^ Copyright 2020, John Wiley and Sons).

Composite materials have attracted considerable interest due to their capacity to integrate various components with unique characteristics, enabling a diverse array of practical applications.^[^ ^173^ ^]^ The significance of non‐crystalline MOF composites lies in their capacity to provide tailored properties that surpass those of their separate components.^[^ ^20^, ^174^ ^]^ As shown in **Figure** **14**, the existing non‐crystalline MOF composites can be categorized into three distinct combinations: the utilization of non‐crystalline MOF as a host matrix for dispersing functional materials, the incorporation of non‐crystalline MOF as the dispersed phase within a matrix, and the integration of non‐crystalline MOF with macroscopic substrates.^[^ ^101^ ^]^

Non‐crystalline MOFs have the potential to act as an appropriate matrix for incorporating dispersed functional materials, such as additional MOF components or non‐MOF components. Many synthetic pathways can be used to accomplish the formation of non‐crystalline MOF matrices that include secondary MOF components. For example, MOF blends are synthesized by combining two glass‐forming crystalline MOFs. Subsequently, the matrix is subjected to elevated temperatures beyond the individual *T*_m_ of both constituents, creating the l‐MOF blend. Following this, the mixture can be quenched to get the g‐MOF blend.^[^ ^53^, ^175^ ^]^ However, melting crystalline MOF is limited because a majority of crystalline MOFs experience thermal decomposition before they can reach their theoretical *T*_m_. Hence, an innovative method called flux melting MOF has been developed. This method entails the incorporation of a meltable MOF to reduce the *T*_m_ and enable the conversion of a non‐melting MOF into a molten state. This method involves subjecting a meltable MOF to elevated temperatures above its *T*_m_ in the presence of a non‐melting MOF. Throughout this method, the melted MOF assumes a pivotal function in stabilizing the dissociated intermediates, thus resulting in a decrease in the *T*_m_ of the non‐melting MOFs. Ensuring that the thermal *T*_d_ of non‐melting MOFs surpasses the *T*_m_ of the meltable MOF component is a crucial factor to consider in the successful fabrication of flux‐melted l‐MOF composites. After undergoing the flux‐melting process, the resultant composite material can be subjected to quenching, producing a composite material consisting of a flux‐melted g‐MOF.^[^ ^55^, ^176^ ^]^ Another distinct category of composites is formed by incorporating a dispersed crystalline MOF as the filler phase and a meltable MOF as the matrix. When the MOF is melted, it transforms into l‐MOF, while the dispersed crystalline MOF retains its original crystal structure, forming crystalline MOF/l‐MOF composites. To achieve this, have a higher *T*_d_ than the *T*_m_ of the meltable MOF. Additionally, the meltable MOF matrix must also exhibit stability across a wide temperature range while preventing flux melting between the two components to ensure the preservation of crystallinity. Furthermore, if the resulting composite material can be rapidly cooled, it will yield a composite material consisting of crystalline MOF/g‐MOF composites.^[^ ^54^, ^177^ ^]^

On the other hand, numerous methods can be employed to create non‐crystalline MOF matrices that incorporate secondary non‐MOF components such as metal halide perovskites (MHPs) and inorganic glasses.^[^ ^101^ ^]^ For instance, when the utilization of non‐crystalline MOFs offers a dual advantage in terms of preserving fillers against various external stressors, including organic solvents, water, air, light, and heat, as well as efficiently mitigating interfacial defects through interfacial bonding, the incorporation of MHPs as fillers becomes a viable option. The fabrication of MHP/l‐MOF composites requires liquid‐phase sintering of the two components, which is a feasible and straightforward method for interface engineering. The interfacial connection between two separate phases leads to stabilising the metastable perovskite phase, which exhibits optoelectronic activity within the composite material. The stability of the embedded perovskites significantly enhances the optoelectronic efficiency by several orders of magnitude. Moreover, the quenching method can accomplish the conversion of l‐MOFs into g‐MOFs and, consequently, the formation of MHP/g‐MOF composites.^[^ ^178^ ^]^ Combining non‐crystalline MOFs with inorganic glasses is possible due to their processability, hence facilitating the combination of the chemical diversity of MOFs with the inherent thermal, chemical, mechanical, and electrical capabilities of inorganic glasses. One need for producing a composite material consisting of inorganic glass and a meltable MOF is that the glass's *T*_g_ should be near the *T*_m_ of the meltable MOF. This proximity enables the production of interfacial bonding inside the inorganic glass/l‐MOF composites. In cases where l‐MOF undergoes a transition to g‐MOF by quenching and subsequently forms inorganic glass/g‐MOF composites, it has been shown that these composites exhibit both glassy domains and the glasses observed to bond at their interfaces. However, there is a restricted intersection among the glassy regions.^[^ ^179^, ^180^ ^]^

The use of non‐crystalline MOFs as a dispersed phase can potentially improve the performance of many materials, such as organic or inorganic substances, as a matrix. The non‐crystalline composite of MOF/organic or inorganic substances is captivating because of the mechanical strength and scalability that polymers provide to non‐crystalline MOFs, along with the enhancements in separation and ionic conduction capabilities due to the possibility of non‐crystalline MOFs’ porosity.^[^ ^181^, ^182^, ^183^, ^184^ ^]^ The effective dispersion of non‐crystalline MOFs inside matrices can be facilitated by the probable absence of inherent distinctions between a‐MOFs or g‐MOFs when combined with organic or inorganic substances. However, suppose there are inherent distinctions between a‐MOFs or g‐MOFs when integrated with organic or inorganic substances. In that case, it can potentially result in defects at the interface between these classes of non‐crystalline MOFs and polymers.^[^ ^185^ ^]^ This has the potential to negatively impact the characteristics of the composite material. On the other hand, the inherent features of a l‐MOF, such as its viscosity, flow behaviour, and reactivity, provide a promising avenue for the precise control and adjustment of interfacial properties inside composite materials.^[^ ^54^, ^181^ ^]^

The integration of non‐crystalline MOFs onto macroscopic substrates like polymers, glasses, amorphous oxides or crystalline MOFs has been explored to develop coatings that can enhance a wide range of functionalities and characteristics. This methodology tackles the processability obstacles associated with crystalline MOFs. It represents an effective strategy for fabricating non‐crystalline MOF devices that exhibit improved capabilities in separation, electronics, and sensing. The substrates play a crucial part in imparting mechanical stability to facilitate the formation of durable composites, which also require strong interfacial interactions and favorable wetting characteristics with the non‐crystalline MOFs. Various methods can be used to generate non‐crystalline MOF coatings on substrates. The methods used include direct growth, deposition, mechanochemical syntheses, electrochemical syntheses, and layer‐by‐layer, spin, or dip coating onto substrates to create a‐MOFs/substrate composites. It is also possible to use another approach that involves in situ solvothermal synthesis to generate glass‐forming crystalline MOFs, which are then melted to form l‐MOFs/substrate composites. The resulting composite structures are then quenched to make g‐MOFs/substrate composites. Various methods can be used to generate non‐crystalline MOF coatings on substrates. The methods used include direct growth, deposition, mechanochemical syntheses, electrochemical syntheses, and layer‐by‐layer, spin, or dip coating onto substrates to create a‐MOFs/substrate composites.^[^ ^101^ ^]^ It is also possible to use another approach that involves in situ solvothermal synthesis to generate glass‐forming crystalline MOFs, which are then melted to form l‐MOFs/substrate composites. The resulting composite structures are then quenched to make g‐MOFs/substrate composites. It is important to note that in this particular situation, the substrates must demonstrate thermal stability to mitigate fractures and defects in the resultant g‐MOF film coating. The melt‐quenching method is highly effective in eliminating defects at the inter‐grain boundaries, resulting in a significant enhancement of the separation capacities within the composite structure.^[^ ^56^ ^]^

The non‐crystalline MOF composites have significant potential due to their exhibited characteristics. The potential of these composites is to increase the properties and performance of a wide range of materials, including mechanical and thermal stability, durability,^[^ ^46^, ^82^ ^]^ electrical conductivity,^[^ ^186^ ^]^ charge storage capacity,^[^ ^187^ ^]^ selectivity,^[^ ^188^ ^]^ and reactivity.^[^ ^189^ ^]^ Therefore, these advancements can significantly impact numerous applications, including energy storage, catalysis, and gas separation. The current state of non‐crystalline MOF composites' research is in its early stages, but it has generated significant interest in understanding its synthesis methods and their applications. Table S4 presents a summary of the major non‐crystalline MOF composites.

## 5 Practical Applications and Opportunities of Non‐Crystalline MOFs

**Figure 15:**
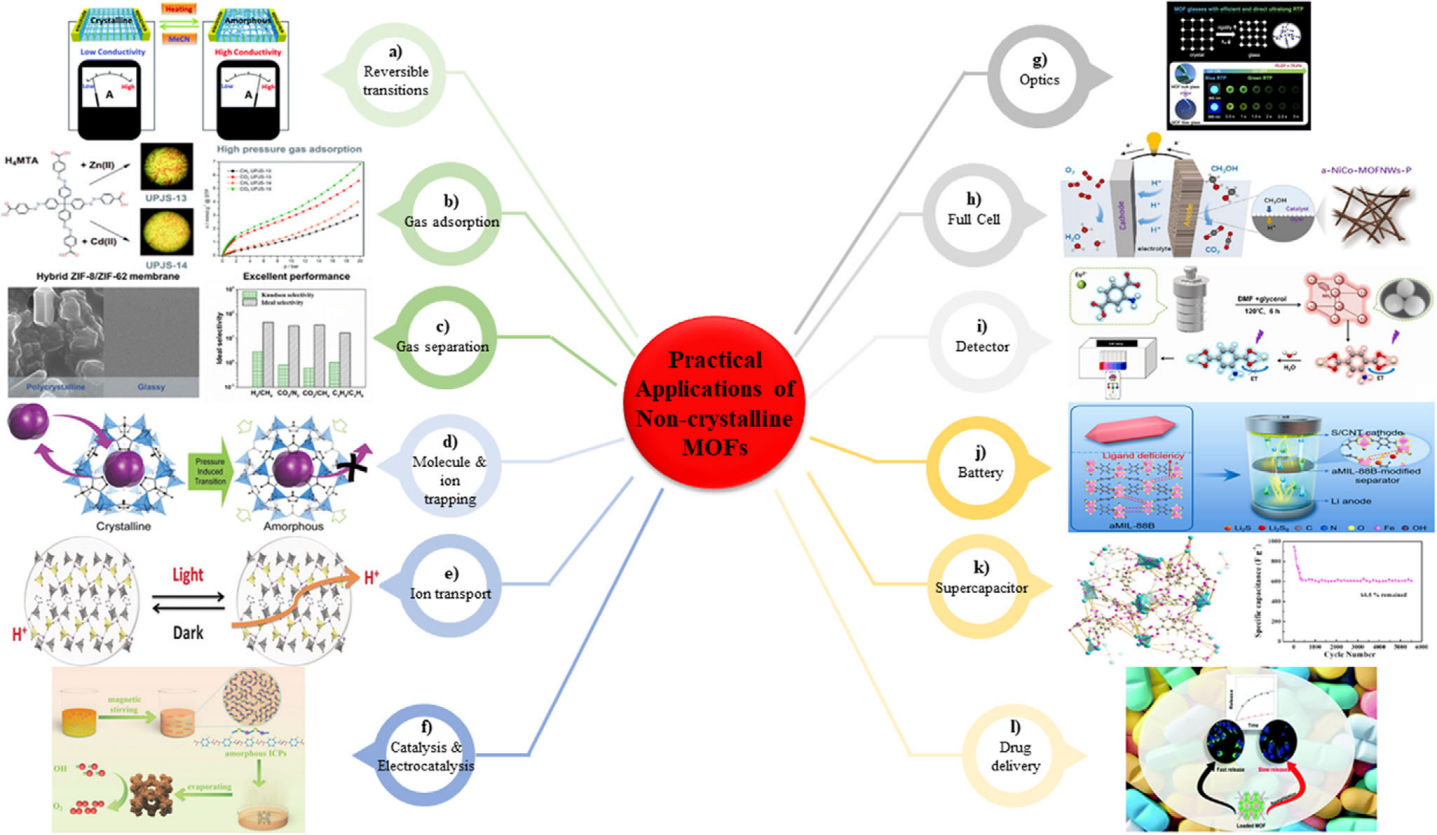
A summary of the major practical applications of non‐crystalline MOFs (Image for a): Reproduced with permission.^[^ ^150^ ^]^ Copyright 2017, RSC; Image for b): Reproduced under CC‐BY Creative Commons Attribution 3.0 Unported License.^[^ ^198^ ^]^ Copyright 2021, RSC; Image for c): Reproduced with permission.^[^ ^31^ ^]^ Copyright 2022, RSC; Image for d): Reproduced with permission.^[^ ^30^ ^]^Copyright 2011, ACS; Image for e): Reproduced with permission.^[^ ^199^ ^]^ Copyright 2017, John Wiley and Sons; Image for f): Reproduced with permission.^[^ ^194^ ^]^ Copyright 2019, RSC; Image for g): Reproduced with permission.^[^ ^200^ ^]^ Copyright 2022, Wiley; for h): Reproduced permission.^[^ ^196^ ^]^ Copyright 2023, Elsevier; Image for i): Image for Reproduced with permission.^[^ ^197^ ^]^ Copyright 2022, Elsevier; j): Reproduced with permission.^[^ ^186^ ^]^ Copyright 2021, Elsevier; Image for k): Reproduced with permission.^[^ ^85^ ^]^ Copyright 2018, Elsevier; Image for l): Reproduced with permission.^[^ ^29^ ^]^ Copyright 2015, RSC).

Non‐crystalline MOFs provide numerous opportunities for practical implementations in a variety of applications, as presented in **Figure** **15**. This opens up an intriguing field of study that leverages their lack of crystallinity to develop novel products.^[^ ^56^ ^]^ Non‐crystalline MOFs demonstrate significant advantages in the areas of gas adsorption and separation. The ability to selectively adsorb and separate certain gases from mixtures presents opportunities for improved gas adsorption and separation.^[^ ^20^, ^56^, ^120^ ^]^ Non‐crystalline MOFs can potentially trap specific molecules and ions, enabling a range of chemical reactions, separation techniques, and the selective capture of desired species.^[^ ^190^ ^]^ These materials have the potential to enhance ion transport rate and reliability, hence creating a significant impact on energy storage devices such as supercapacitors and batteries. The tunable characteristics of these materials, their semiconducting capabilities, and appropriate surface area can potentially improve energy density, stability, and safety. This might lead to developing more efficient and dependable energy storage devices.^[^ ^56^, ^85^, ^191^ ^]^ The capacity of non‐crystalline MOFs to undergo reversible transitions between distinct structural states provides adaptability to dynamic situations or demands, a characteristic of crucial significance for responsive materials.^[^ ^150^, ^192^ ^]^ Moreover, these materials have shown their significance in catalysis and electrocatalysis by offering tailored active sites and reactive surfaces for a wide range of chemical and electrochemical reactions.^[^ ^20^, ^193^, ^194^ ^]^ The utilization of non‐crystalline MOFs in drug delivery exhibits considerable promise owing to their distinctive surface area and adaptability combination. They have the potential to significantly increase the capacity for drug loading and enable precise control of release rates, hence enhancing the effectiveness and efficiency of treatments.^[^ ^29^, ^56^, ^195^ ^]^ Non‐crystalline MOFs in optics possess distinct properties such as isotropic homogeneity, processability, and transparency. These particular characteristics enable the creation of functional materials that possess extraordinary features, such as luminescence or the ability to switch functions, when paired with sensitive dopants. Furthermore, these materials have potential in the field of nonlinear optics, rendering them well‐suited for various photonics applications such as sensing and optical modulation, particularly within the near‐infrared spectral range.^[^ ^20^, ^56^ ^]^ Moreover, non‐crystalline MOFs have strong potential for use in fuel cells because they exhibit excellent proton conductivity, long‐lasting durability, effective charge transport pathways, many active sites, faster electron transport capabilities, and improved reaction kinetics. These features collectively enhance the overall performance and sustainability of fuel cell devices.^[^ ^97^, ^196^ ^]^ Non‐crystalline MOFs also have significant potential for use in detectors because of their adjustable characteristics, quick response times, stability, and adaptability to various detecting techniques. Due to their capacity to enhance sensitivity, selectivity, and reliability, they are highly suitable for developing detection technologies in many industrial, environmental, and biological areas.^[^ ^89^, ^197^ ^]^ Despite the promising potential of non‐crystalline MOFs, significant opportunities remain for their further development to fully realize their capabilities. Researchers are continuously exploring innovative synthesis methods and properties modification techniques to improve the performance of non‐crystalline MOFs for specific applications. As material science continues to advance, new applications for non‐crystalline MOFs are expected to emerge, making this an intriguing and active area of research. Table S5 summarizes the major practical applications of non‐crystalline MOFs.

## 6 Challenges and Prospects in Non‐Crystalline MOF Research

Despite the potential benefits of non‐crystalline MOFs, there are still several major research gaps that necessitate attention in order to fully harness their capabilities, such as the study of novel synthesis methods, characterization techniques, structure‐property relationships, multifunctional materials, stability and durability, scalability, and application studies.^[^ ^20^ ^]^ Non‐crystalline MOFs are typically produced by non‐traditional methods as well and the synthesis of these materials is still not well understood. Therefore, there is a great need for the establishment and development of standardized procedures in the synthesis of non‐crystalline MOFs. This will guarantee reproducibility, commercial viability through scalability and cost‐effectiveness, and facilitate the customization of the desired characteristics, thereby unlocking substantial potential for innovative applications.^[^ ^3^ ^]^ The production of non‐crystalline MOFs on a large scale needs the optimization of synthesis conditions and the development of cost‐effective precursors. It is also crucial to establish efficient purification and activation methods to ensure consistent quality on a large scale. Furthermore, production methods that are efficient are essential for large‐scale applications, and economies of scale can considerably reduce costs, making non‐crystalline MOFs more accessible for industrial use. Finally, their scalability for wide implementation could be further enhanced by advancements in automation and process engineering, which could accelerate production.^[^ ^20^, ^201^ ^]^

Moreover, the properties of non‐crystalline MOFs have been elucidated through various characterization techniques; however, there remains a significant knowledge gap in this area. Indeed, non‐crystalline MOFs cannot to characterized using traditional techniques, and new methods with standardized procedures are needed to fully understand their structure and properties.^[^ ^202^ ^]^ The establishment of standard procedures for the characterization of non‐crystalline MOFs is crucial in order to guarantee consistency and comparability among different research studies. The development of novel characterization techniques and tools specifically tailored for non‐crystalline MOFs has the potential to unveil unexplored avenues and facilitate groundbreaking findings within this class of materials. Indeed, the process of scaling up the synthesis of non‐crystalline MOFs presents significant challenges, primarily owing to their complex synthesis and characterization procedures.^[^ ^28^, ^203^, ^204^ ^]^

The structures of non‐crystalline MOFs exhibit disorder, posing challenges in establishing a definitive correlation between their structure and properties.^[^ ^3^, ^205^ ^]^ The structural disorder in non‐crystalline MOFs leads to heterogeneity, which could result in inconsistent properties across various batches. These variations present challenges to the reproducibility of materials. To address these challenges, it is essential to use standardized synthesis protocols and advanced characterization techniques such as PDF analysis. These methods improve repeatability by maintaining uniform disorder levels and providing detailed insights into local structures.^[^ ^205^ ^]^ Inconsistencies in material properties due to structural heterogeneity can prevent the development of standardized products and disturb quality control, making it difficult to ensure consistent performance across various batches. Overcoming these issues requires innovative strategies to monitor and control structural variations during synthesis. To effectively design and optimize non‐crystalline MOFs for specific applications, it is vital to acquire a deeper understanding of the structure‐property relationships. Nowadays, there is a burgeoning interest in the advancement of multifunctional materials capable of executing multiple tasks simultaneously. Therefore, the investigation of non‐crystalline MOFs in multifunctional materials has the potential to yield novel prospects and employment in various applications.^[^ ^20^ ^]^ A significant constraint associated with non‐crystalline MOFs is related to their limited stability and durability. The possible effects of stability and durability issues include the risk of structural collapse or hindered functionality in specific conditions.^[^ ^22^, ^35^ ^]^

Strategies for improving stability include the selection of metal ions and ligands that form strong bonds, the modification of functional groups to enhance thermal resistance, and the control of synthesis conditions to reduce defects. In addition, post‐synthetic treatments, such as heating, can enhance stability by removing unstable components. Materials that can endure severe environmental conditions are needed for applications in a variety of environments, and durability is the primary factor in ensuring long‐term reliability and performance. Furthermore, their resistance to degradation over time could be further enhanced by the incorporation of composite structures or protective coatings. The a‐MOFs can be made more durable for industrial applications by leveraging their inherent mechanical robustness.^[^ ^206^, ^207^ ^]^ Exploring strategies to enhance the stability and durability of these materials has the potential to facilitate wider acceptance and novel application. Although non‐crystalline MOFs hold promise for diverse applications such as gas sorption, catalysis, and energy storage owing to their unique properties, there is still a need to fully investigate and understand their complete range of capabilities.^[^ ^20^ ^]^ Further investigation is required in order to cultivate novel and inventive applications for these materials.

For example, the non‐crystalline nature of these MOFs offers benefits such as improved processability and flexibility, making them well‐suited for the development of uniform thin films that are essential for applications such as electronic devices or gas separation membranes.^[^ ^208^ ^]^ However, their development is still in its early stages. Specialized techniques are necessary to ensure uniformity and prevent unintended crystallization when synthesizing non‐crystalline MOF thin films. Furthermore, it is necessary to preserve their stability under different environmental conditions to promote their practical application.^[^ ^209^, ^210^ ^]^ Traditional techniques, such as XRD, are ineffective for these materials, which present additional challenges in the context of characterization. Therefore, to investigate their structure and properties, it is necessary to employ advanced techniques such as PDF analysis and spectroscopy. Another limitation is scalability, as the current laboratory‐scale synthesis methods are not readily adapted to industrial production. The key to unleashing the widespread adoption of these materials will be the development of cost‐effective and scalable methods, such as solution‐based deposition or vapor‐phase methods. Therefore, to effectively address all these research gaps, it will be necessary to promote innovation across multiple academic disciplines, encompassing materials science, chemistry, and engineering.

## 7 Conclusion and Outlook

Non‐crystalline MOFs represent a promising research avenue for the development of exciting new materials with significant promise and potential across diverse applications. Over the past few years, the concept of non‐crystalline MOFs has garnered considerable attention owing to their distinctive properties, prompting researchers to conduct thorough investigations into their design, synthesis, characterization, properties evaluation, and applications. Addressing the challenges and limitations affecting their effectiveness and performance through practical considerations is essential. Therefore, future research on non‐crystalline MOFs is expected to adopt a variety of approaches. As a means toward improving the yield, properties, reproducibility, and scalability of non‐crystalline MOFs, novel synthesis methods can be developed. Computational techniques, such as molecular dynamics models and machine learning algorithms, offer valuable tools for characterizing the structure and properties as well as guiding the synthesis and optimization of non‐crystalline MOFs. While non‐crystalline MOFs have shown promise in applications like catalysis, gas storage, and separation, many potential uses remain unexplored. Therefore, another approach could focus on identifying and investigating new applications for non‐crystalline MOFs.

## Conflict of Interest

The authors declare no conflict of interest.

## Supporting information



Supporting Information
