# Supplementary material for: Practical Considerations in the Design and Use of Non‐Crystalline Metal–Organic Frameworks
Source: Adv Mater. 2025 Jul 28;37(41):e05579. doi: 10.1002/adma.202505579 (PMC12531752; doi:10.1002/adma.202505579)
Supplement: Supplementary file 1 — Supporting Information [file ADMA-37-e05579-s001.docx]

**Supplementary information**

**Practical Considerations in the Design and Use of** **non-****Crystalline Metal-Organic Frameworks**

*Hamidreza Mahdavi^a,b*^, Farnaz Zadehahmadi^b,c^, Mehran Arzani^d^, Leena Melag^e^, Ashley L. Sutton^b^, Muhammad M. Sadiq^b^, Zongli Xie^b*^, Matthew R. Hill^c*^, Benny D. Freeman^a,f*^*

^a^ Department of Chemical and Biological Engineering, Monash University, Clayton, VIC 3800, Australia

^b^ CSIRO Manufacturing, Private Bag 10, Clayton South, VIC 3169, Australia

^c^ Department of Materials Science and Engineering, Monash University, Clayton, VIC 3800, Australia

^d^ Department of Chemical Engineering, University of Illinois at Chicago, 929 W Taylor St, Chicago, Illinois 60607, United States

^e^ CSIRO Mineral Resources, Private Bag 10, Clayton South, VIC 3169, Australia

^f^ John J. McKetta Jr. Department of Chemical Engineering, The University of Texas at Austin, 200 E. Dean Keeton Street, Austin, TX, 78712, USA

E-mail: hamidreza.mahdavi1@monash.edu, zongli.xie@csiro.au, matthew.hill@monash.edu, benny.freeman@utexas.edu, benny.freeman@monash.edu

**Table S1** A summary of the major synthesis methods of non-crystalline MOFs.

| **Non-crystalline MOFs** | **MOF** | **Synthesis method** | **Highlights** | **Ref.** |
| --- | --- | --- | --- | --- |
| a-MOF | ZIF-8 | Pressure-induced amorphization | High compressibility, undergoing irreversible pressure-induced amorphization at remarkably low pressures (1 GPa pa)  Easily scalable and industrially relevant | [1] |
|  | MOF-5 |  | Achievable irreversibly at room temperature and low compression pressure  Inducing irreversible amorphization through the selective destruction of carboxylate groups under pressure | [2] |
|  | ZIF-8, ZIF-mnIm |  | The same topology, ZIF-8 and ZIF-mnIm  Similar sorption abilities  Significant difference in their guest-retention behavior upon heating  Enhanced retention of guest species during amorphization of ZIF-mnIm  I_2_ loss in ZIF-mnIm, up to 200 °C | [3] |
|  | ZIF-8 |  | Hinders functional properties of metal−organic frameworks  The use of trace amounts of spin probes (stable nitroxide radicals)  Efficient monitoring and control of pressure-induced processes  Recognizing the vital role of solvent molecule diffusion within the MOF as a key factor in mitigating amorphization  70% damage in ZIF-8 cavities under1.15 GPa pressure (absence of solvent)  Significant mitigation of amorphization due to impregnation with solvents like toluene or 2-propanol because of its easy penetration through MOF windows  To reach most effective mitigation of pressure-induced amorphization, solvent molecules are present both in the MOF cavities (stabilization of framework) and between MOF particles (pressure equilibration) | [4] |
|  | ZIF-8 |  | Crystal-to-amorphous phase transition under approximately 3 GPa  Retained Zn-N coordination during amorphization | [5] |
|  | MIL-101 |  | An irreversible amorphization at high pressures, but the onset pressure depends on the PTM  Lower amorphization presser by using NaCl (0.4 GPa) than Nujol and silicone oil (7 Gpa)  A slight lattice expansion and alters the hydroxyl vibration frequency and shape due to the insertion of Fluid PTM into the pores of MIL-101 at low pressures  Significant pressure-induced changes due to OH stretching vibrations  Efficient monitoring and control of pressure-induced processes | [6] |
|  | MIL-53(Al) NH2-MIL-53(Al) |  | High pressure resistance and the highest negative linear compressibility  MIL-53(Al) and NH_2_-MIL-53(Al) of SPCs family  The pressure between 5 and 16 GPa for amorphization  The negative linear compressibility behavior | [7] |
|  | calcein @ Zr-based UiO-66 |  | Loaded calcine into UiO-66 using a suspension method  calcein@UiO-66 sample with retained UiO-66 structure  Demonstrated controlled release of calcine over an extended period  Loaded and released hydrophilic model drug calcein into Zr-based MOF UiO-66 | [8] |
|  | ZIF-62 (glass) | Mechanical milling-induced amorphization | low-crystallinity ZIF-62  lots of open metal sites | [9] |
|  | Zr-based family of MOFs |  | Reasonable aqueous stability  low toxicity (Zirconium); the lethal dose (LD50) of zirconyl acetate in rats is less than 4.1 mg mL⁻¹  Modification of adsorption properties by replacement of the original BDC linker with functional groups (e.g., bromo, amino, nitro) | [10] |
|  | UiO-66, MIL-140B, and MIL-140C |  | Retain inorganic-organic bonding motifs similar to crystalline phases  While inorganic Zr_6_O_4_(OH)_4_ clusters of UiO-66 remain intact upon collapse, ZrO backbone of MIL-140 frameworks undergoes substantial distortion. | [11] |
|  | A serious of M-BTC  (M: Fe^3+^, Co^3+^, Co^2+^, Ni^2+^, and Cu^2+^) | Irradiation-induced amorphization | The topology of a metal cluster influences formation rate (1 to 30 min) and chemistry of resulting phases based on analysis of M-BTC series (Fe3+, Co^3+^, Co^2+^, Ni^2+^, Cu^2+^)  Comparison of M-BTC series (Fe^3+^, Co^3+^, Co^2+^, Ni^2+^, and Cu^2+^) reveals insights into the transformation dynamics | [12] |
|  | ZIF-L |  | High purity synthesis  Synthesized as large, uniform flat sheet-like particles, a few μm in length and 100−300 nm in thickness | [13] |
|  | ZIF-4, ZIF-62, and ZIF-zni |  | Complete amorphization on timescales ranging from minutes to hours  Non-isokinetic process in all three cases, with varying transformation rates as the process proceeds  Inhomogeneous nucleation, evidenced by increasing local Avrami exponent over time  Amorphization rate accelerates with increasing temperature, even below usual thermal stability limits of crystalline phases | [14] |
|  | Co-URJC-5 | Chemical treatment-induced amorphization | Amorphization process initiated by unconventional SO_2_ uptake at pyridine sites and, 298 K temperature, and 1 bar pressure  Plausible amorphization mechanism proposed considering affinity of SO_2_ for pyridine molecule and consecutive complete removal of this ligand | [15] |
|  | (Me_2_NH_2_)[Co_3_(Me_2_-NH)_3_(OH)(SDBA)_3_, H_2_SBDA 14 4,40-sulfonyldibenzoic acid |  | Amorphization via removal of coordinated solvent in a Co^II^-MOF  Coordination geometry of Co^II^ and magnetic exchange pathway modified during transition  Crystalline MOF demonstrated spin-canting, spin-flop, and easy plane magnetic anisotropy behaviour  Amorphous sample does not revert to initial crystalline state when immersed in mother solution, but can recover to crystalline after heating in DMF/CH_3_OH solution (1:1) at 120 or 160 °C for 24 hours | [16] |
|  | PCMOF-17 |  | Atmospheric water-induced amorphization  the sulfonated PCMOF-17 framework, underwent amorphization when exposed to relative humidity levels of 60% or greater. | [17] |
|  | UiO-66 |  | Undamaged UiO-66 structure of 2 wt.% Ag catalyst (2Ag-U)  Framework collapse of UiO-66 to due to Ag loading increase to 10 wt.% (10Ag-U)  With further increase in Ag loading, the framework of UiO-66 collapsed, resulting in the formation of an amorphous structure | [18] |
|  | Fe-BTC aerogel (Fe_3_ (III)O(C_6_H_3_(COO)_3_)_2_NO_3_ | Direct synthesis | Permanent micro- and microporosity  Sol–gel approach followed by supercritical CO_2_ drying | [19] |
|  | Zn(ICA)-2 MOF (a-Zn(ICA)-2) |  | Adding triethylamine (TEA) to a dimethylformamide (DMF) solution containing imidazole-2-carboxyaldehyde (ICA) and Zn(NO_3_)_2_6H_2_O at room temperature  The amorphous phase formation was attributed to the direct addition of TEA, which led to the immediate reaction of reagents (Zn^2+^ and ICA) forming numerous coordination polymers (CPs) | [20] |
|  | Two-dimensional (2D) bimetallic Fe_1_Ni_2_(BDC-NH_2_) MOF |  | A mild room temperature (RT) solution phase method  The amorphous Fe_1_Ni_2_(BDC-NH_2_) MOF was physically deposited onto Ni foam, resulting in Fe_1_-Ni_2_(BDC-NH_2_)/NF | [21] |
|  | a-MOF based biomimetic (CA-Cu) nanozyme |  |  |  |
|  | a-MOF  a- CPs |  | Stöber-inspired synthesis adapted for amorphous MOFs and CPs  Provides a modular and scalable platform for designing multifunctional colloids with tunable complexity  Synthesized over 100 core-shell composites | [23] |
|  | a-NiCo-MOFNWs | Two-step pyrolysis- Heat-induced interconnection and Heat-induced phosphorization | Amorphous NiCo MOF nanowires with P doping (a-NiCo-MOFNWs-P)  Formation of directly interconnected NiCo-MOF nanowire networks  Synergistic effect between amorphous MOFs and dispersed phosphorus species | [24] |
|  | CA-Cu nanozyme MOF | Solvothermal | A straightforward solvothermal synthesis involving cyanuric acid and Cu²⁺  Enhanced N-Cu coordination enabling improved laccase- and catecholase-like activity  Mimic the N-Cu coordinated environment between the imidazole and Cu^2+^ in the active site of the enzyme  Exhibited both laccase- and catecholase-like activity | [22] |
|  | NEU-2 = Fe(BPDI)(Py)_2_, BPDI = N,N'bis (glycinyl) pyromellitic diimide; Py = pyridine) |  | Solvothermal MOF synthesis using mixed solvents (pyridine and 2-MeTHF) under autogenous pressure | [25] |
| l-MOF | ZIF-4 | Direct melting synthesis | The MOF liquid retains the chemical configuration, coordinative bonding, and porosity of the parent crystalline framework  Based on mean square displacement plots, diffusive behaviour above 1,200 K, indicating the liquid phase  Analysis of linker rotations indicated a regime of free rotation of imidazolate linkers at intermediate temperatures before melting | [26] |
| g-MOF | cobalt-ZIF-62 | Melt quenching | (Co(imidazole)_1.75_(benzimidazole)_0.25_) onto silicon nanoparticles was followed by melt-quenching to create a Si@ZIF-glass composite (SiZGC) | [27] |
|  | ZIF62 |  | High crystal-glass network density deficiency  No crystallization in supercooled region  Low fragility  Extremely high Poisson’s ratio  Highest T_g_/T_m_ ratio  An increase in T_m_ and T_g_ with benzimidazolate (bIm) concentration  T_g_/T_m_ ratio maintains the same  Very low enthalpy and entropy | [28] |
|  | CdTz | Mechanically induced amorphization | Comprised of discrete Zn^2+^ complexes instead of a networked structure  The glassy states of CP crystals are influenced by various structural parameters | [29] |
|  | ZIF-62 | Melt quenching | Lowering Tm by adding varying quantities of larger organic ligands  Mechanism involves partial linker decoordination; melting temperature partially dependent upon metal-ligand bond strength  Increasing size of organic ligand predicts effect on elastic moduli of glasses formed, with decrease in elastic moduli upon increasing size of organic component | [30] |
|  | (MIL-53)x(agZIF-62)_1–x_ | Mechanically induced amorphization | MOF-CGC series, denoted as (MIL-53)_x_(agZIF-62)_1−x_, where x = 0.25 and also 0.3 ≤ x ≤ 0.9 in 0.1 increments, were synthesized  MIL-53-np phase obtained by heating MIL-53-as to 330 °C for 72 hours | [31] |
|  | g-CP  (diethylmethylammonium )_0.35_[Zn(H_2_PO_4_)_2.35_(H_3_PO_4_)_0.65_] | Direct synthesis of glasses | Zn^2+^ and protic ionic liquid (dema)(H_2_PO_4_) react to form a Zn^2+^– H_2_PO_4_/H_3_PO_4_ network structure, with dema cations surrounded by this CP network | [32] |
|  | g-ZIF8 | Melting | [EMIM][TFSI]) as ionic liquid (IL) incorporation in ZIF-8 lowers melting point below decomposition temperature  IL interactions stabilize ZIF-8 linkers during thermal dissociation  Temperature and time critically influence IL@ZIF-8 glass and crystal–glass composite formation | [33] |
|  | g-ZIF8 | Melting modified with heterocycle-based halide salts | Co-melting ZIFs with heterocycle-based halide salts enables MOF glass formation, without requiring phase-pure crystalline precursors  Salts act as chemical modifiers, promoting ZIF-8 melting by preventing decomposition  Enables structural and property control  Co-melting with H_2_bImCl enables glass formation of ZIF-4, ZIF-8, and ZIF-62 at 300 °C, lower than typical ZIF melting points | [34] |
|  | MOF glasses from carboxylate-based frameworks | melt-quenching | Melt-quenched MOF glasses from carboxylate-based frameworks (Mg^2+^/Mn^2+^)  Low melting temperatures: 284 °C (Mg) and 238 °C (Mn) | [35] |
|  | g-Aluminum alkoxide | Bottom-up solvent evaporation approach | Aluminum alkoxide glasses formed by linking Al-oxo clusters with alcohol linkers  Bulky alcohol modulator acts as network plasticizer and pore template | [36] |
|  | g-MOF | Desolvation | This technique enables formation of g-MOFs under inert conditions  Suppressing coordinated solvents drives structural rearrangement into metastable, glassy networks  Offers improved processability and structural control for g-MOF applications | [37] |

**Table S2** Properties evaluation of non-crystalline MOFs.

| **Properties** | | **Non-crystalline MOFs** | **Components** | **Highlights** | **Ref.** |
| --- | --- | --- | --- | --- | --- |
| Structural | Bonding, crystal structure, and morphology features | a-MOF | MOF-5 | Before amorphization, the sample pressure in typical cubic crystal structure (the structure changed with low pressure in this method)  Reduction in intensity enhances with increasing pressure  Nearly identical positions for the five strong Raman bands for both amorphous MOF-5 and the crystal one with different intensities | [2] |
|  |  | a-MOF | ZIF-8 | Pressure release just before amorphization leads to a swing effect causing an isostructural crystal-to-crystal phase transition, consistent with experiments  No phase change, but potential system fracture suggested above 10 GPa  No perceived phase transition up to 2.75 GPa in tensile regime  Structural failure at 2.75 GPa  Crystal-amorphous phase transitions detected at around 4 GPa under uniaxial compression  Amorphous structures formed under uniaxial stress are about 20% denser than those formed under hydrostatic pressure | [5] |
|  |  | a-MOF | MIL-53(Al) NH_2_-MIL-53(Al) | The pressurization medium (such as water, ethanol, or argon) influenced the degree of NLC and the phase transition of the MOFs | [7] |
|  |  | a-MOF | Zr-based family of MOFs | PBS-treated MOFs showed presence of phosphate groups around 1000 cm⁻¹  An exchange of linkers for phosphate groups occurred, indicating coordination between Zr and PO_4_^3⁻^  Intensity of phosphate group peaks increased over time, while carboxylate group peaks decreased | [10] |
|  |  | a-MOF | UiO-66, MIL-140B, and MIL-140C | Emergence of a band centered at approximately 1700 cm⁻¹ upon amorphization of MIL-140 samples  Assigned to uncoordinated carbonyl stretching frequency  Increase in intensity of this band noted upon collapse of UiO-66, where uncoordinated bdc ligands within pores lead to a small feature at 1700 cm⁻¹ in crystalline sample  UiO-66:  Experimental spectrum reveals three peaks  Resonances at 128 ppm and 137 ppm attributed to two types of carbon on bdc aromatic ring  Signal at 170 ppm assigned to carboxylate-binding group.  MIL-140B:  Experimental spectrum shows increased complexity compared to UiO-66  Multiple signals in 120-140 ppm region, with two distinct resonances at 173.5 ppm and 175 ppm  MIL-140 ℃:  Spectrum exhibits further complexity compared to MIL-140B  Contains three resonances in region 170-175 ppm | [11] |
|  |  | a-MOF | ZIF-L | At doses above 100 e/Å^2^, a second stage of electron-beam-induced modifications occurs, where the internal structure of 2-mIm linker molecules begins to break down  Bond breaking within the organic linker was identified through core-loss EELS C and N K-edge analysis | [13] |
|  |  | a-MOF | ZIF-4, ZIF-62, and ZIF-zni | No significant amorphization with 29 keV beam exposure, contrary to complete amorphization observed with 20 keV X-rays  Remarkable difference attributed to radiation dose, increased due to larger cross section of materials at lower photon energies and differences in photon flux between facilities  No straight lines, Avrami plots of amorphization reaction for ZIF-4, ZIF-62, and ZIF-zni, indicating deviations from ideal behavior  Departures from ideal Avrami behavior may stem from spatially non-homogeneous nucleation, anisotropic growth, and time-dependent nucleation coupled with size-dependent growth  Linear evolution of n with x for ZIF-4, but non-linear for ZIF-62, despite structural similarity  Synchrotron X-rays triggered mechanism leading to breakdown of both porous and dense crystalline MOFs to amorphous phases | [14] |
|  |  | a-MOF | Co-URJC-5 | Coordination bond breakage between pyridine and cobalt after SO_2_ exposure  Crystal structure collapses when exposed to SO_2_ or heated to 398 K  No crystalline rearrangement was achieved in any case, suggesting the absence of Open Metal Sites (OMS) in the new Co(II) coordination sphere  The recovery of the crystalline phase by exposuring of CoMOF to dimethylamine (DMA) vapor | [15] |
|  |  | a-MOF | (Me_2_NH_2_)[Co_3_(Me_2_-NH)_3_(OH)(SDBA)_3_, H_2_SBDA 14 4,40-sulfonyldibenzoic acid | Achievement of conversion into neutral framework 2 without structural disruption  Simultaneous removal of coordinated DMA, DMA^+^ ions, and OH^-^ ions  Deionization leading to changes in coordination geometry, magnetic exchange pathway, color, magnetism, and dielectric constant  weight loss up to 342 °C, corresponding to loss of three-coordinated dimethylamine (DMA) molecules and two ionic groups (DMA^+^ and OH^-^) | [16] |
|  |  | a-MOF | PCMOF-17 | The MOF incorporates hydrogen-bonded dimethylammonium cations and water molecules  The MOF becomes amorphous in >60% relative humidity | [17] |
|  |  | a-MOF | CA-Cu nanozyme | CA:Cu ratio  1:3: Smooth area, uniform heteromorphic hexagonal prism  1:2: Fuzzy and rough with defects  1:1: Main structure collapse, high defects  1:0.5: Complete morphological transformation into a wool ball-like structure  Crystallinity loss with reduced Cu²⁺, forming fully amorphous CA-Cu (1:0.5) | [22] |
|  |  | a-MOF | M[Ni(CN)_4_]:MNi; M = Mn, Fe, and Co | Upon hydration, the coordination geometry of these metal centers changed from tetrahedral to octahedral, resulting in significant reorganization of the MOF local structure  Ni[Ni(CN)_4_] (NiNi) containing square-planar Ni^2+^ centers did not undergo significant structural transformation and therefore abruptly adsorbed H_2_O in the low-pressure region  How changes in the bond lengths and coordination geometry are related to the adsorption properties of amorphous MOF systems | [38] |
|  |  | a-MOF | UIO-66-NH_2_ | Amorphous material with a carbonized structure  Contains abundant Zr^4+^ active sites | [39] |
|  |  | a-MOF | Cu-BTC | The sample undergoes expansion, causing the relatively compliant axial Cu-O bond (Cu-O_2_) to increase in length  At 3.9 GPa, despite more solvent entering the pore, the unit cell volume and axial Cu-O bond contract  High pressure forces the solvent out of the pores, accommodated by the extension of the compliant (axial) Cu-O_2_ bond  The transition from pore filling to pore emptying mechanism is associated with the sudden compressibility of the stiffer equatorial Cu-O bonds  Equatorial Cu-O bonds exhibit resilience to compression up to 3.9 GPa  These bonds only contract when pressure is increased to 5.0 GPa | [40] |
|  |  | g-MOF | ZIF-62/PEI MMMs | The proportion of zinc acetate increases from 10% to 40%  An initial increase in the intensity of the diffraction peaks in ZIF-62-L, subsequently declines, indicating a fluctuation in the crystallinity of the produced materials  Maximum peak intensity of ZIF-62-L peaks at a 30% proportion of zinc acetate  The presence of a diffraction peak associated with ZnO when the proportions of zinc acetate are 10%, 20%, and 40%, suggesting the existence of ZnO impurity in the respective samples  Irregular particle shapes due to incomplete ZnO conversion at 10% and 20% zinc acetate  At 30% zinc acetate, ZIF-62-L particles exhibit clear outline, distinct shape, and no observable impurities  At 40% zinc acetate, presence of large regular particles and small particles (possibly ZnO impurity)  Optimal zinc acetate ratio determined as 30% for suitable particle size and absence of ZnO impurity in liquid-assisted mechanochemical synthesis  Consistent ratios of HIm and HBIm linkers observed in both ZIF-62 and ZIF-62-L  A lower binding energy for the Zn 2p peak in ZIF-62-L, indicating the presence of more open metal sites compared to ZIF-62 | [9] |
|  |  | g-MOF | g-CP (diethylmethylammonium)_0.35_[Zn(H_2_PO_4_)_2.35_(H_3_PO_4_)_0.65_] | Extended networks prohibit movement of both dema cations and H_2_PO_4_ anions, preventing hydrogen bond formation | [32] |
|  |  | g-MOF | ZIF-8 | a_g_(IL@ZIF-8-HT) shows glassy structure with diffuse scattering, while pure ZIF-8 remains crystalline under identical HT treatment | [33] |
| Framework | Pore size, shape, and porosity | a-MOF | ZIF-8 | Retains its nanoporosity while retaining some structural order  A degree of homogenization in pore and window/aperture dimensions  Far-reaching changes in pore dimensions | [1] |
|  |  | a-MOF | ZIF-8 | A small percentage of damaged pores (20%) under optimum conditions (1.15 GPa) | [3] |
|  |  | a-MOF | ZIF-L | At lower electron doses (<100 e/Å^2^), ZIF-L with collapsed pores retains the chemistry of the parent ZIF, as confirmed by core-level EELS analysis. | [13] |
|  |  | a-MOF | PCMOF-17 | While much of MOF chemistry focuses on designing pores in solids, the pore structure in PCMOF-17 does not provide an efficient proton transfer pathway and instead restricts proton conduction. | [17] |
|  |  | a-MOF | UiO-66 | Specific surface area of UiO-66 decreased from 473 m^2^ g^-1^ to 13.6 m^2^ g^-1^ as weight percentage of Ag increased. | [18] |
|  |  | a-MOF | Fe-BTC aerogel (Fe_3_ (III)O(C_6_H_3_(COO)_3_)_2_NO_3_ | Specific surface area and total pore volume of the aerogel: 1618 m² g⁻¹ and 5.62 cm³ g⁻¹, respectively.  The air-dried powdered sample (xerogel) exhibited a specific surface area and total pore volume of 1183 m² g⁻¹ and 0.71 cm³ g⁻¹, respectively. | [19] |
|  |  | a-MOF | Zn(ICA)-2 MOF (a-Zn(ICA)-2) | These CPs formed irregular nanospheres with diameters around 20 nm, building a superstructure, and mesopores were randomly generated between the nanoparticles.  The average mesopore diameter was calculated to be about 6–9 nm, and the BET surface area of a-Zn(ICA)-2 was reported as 251 m² g⁻¹. | [20] |
|  |  | a-MOF | a-NiCo-MOFNWs | Increased porosity for mass transfer  Durable self-supporting porous structure. | [24] |
|  |  | a-MOF | NEU-2 | Exhibits a hierarchical pore distribution with micropores, mesopores, and macropores. | [25] |
|  |  | a-MOF | MOF-5 | Further supported by the collapse of pores  A significant decrease in the surface area (S_BET_ = 3450 m^2^/g to 6 m^2^/g) due to an increase in pressure of compressing treatments | [41] |
|  |  | a-MOF | ZIF-69, ZIF-mnIm | Retained their previous pore spaces up to 200 ℃ | [42] |
|  |  | a-MOF | MIL-100(Fe)  with reactants: MF (presence of HF), MNF1 (absence of HF and lower water content), MNF_2_ (absence of HF) | The pore structure of the carrier and the route of synthesis have a significant effect on the loading and release of DOX from MIL-100(Fe) carriers | [43] |
|  |  | a-MOF | UPJS-13  UPJS-14 | Freeze-dried materials activated at 80°C showed the largest surface areas: 830 m^2^ g^−1^ for UPJS-13 (FD) and 1057 m^2^ g^−1^ for UPJS-14 (FD) | [44] |
|  |  | a-MOF | MIL-53(Al) NH_2_-MIL-53(Al) | A wine-rack structure allows MIL-53(Al) to change their pore size in response to different stimuli, such as adsorption, temperature, or pressure | [45] |
|  |  | l-MOF | ZIF-4 | Porosity evolution, from 300 K to higher temperatures showed maintenance of overall porosity in the liquid phase, with a slight deviation in average pore volumes  A large fraction of void space in the ZIF liquid as accessible porosity (74% at 300 K to 95% at 1,500 K) | [26] |
|  |  | g-MOF | g-Aluminum alkoxide | Modulator removal yields gas-accessible pores with surface areas up to 500 m^2^/g | [36] |
|  | Flexibility and rigidity, and stimuli responsiveness | g-MOF | CdTz | Demonstrates great potential of glassy state of CP crystals in enhancing physical functions (conductivity, dielectric constant) and optical properties dependent on transparency and material flexibility | [29] |
|  |  | g-MOF | ZIF-62 | Increase in rigidity expected due to similar density  Suggests better structural equilibration of the glass formed from a higher temperature melt, despite identical cooling rates | [30] |
|  |  | a-MOF | ZIF-4 | Despite ZnN_4_ tetrahedra being less rigid than SiO_4_ tetrahedra, the primary modes of flexibility involve the movement of the zinc atom out of the plane of the imidazole ring or the twisting of the imidazole ring out of its original plane  ZnN_4_ tetrahedra in any of the ZIF structures are less rigid compared to the corresponding tetrahedra in amorphous silica | [46] |
|  |  | a-MOF | Bio-MOF-100 | Flexible building blocks that possess a high Q factor of approximately 10^4^ | [47] |
|  |  | a-MOF | MOF-5 | Frustrated flexibility" in MOFs, which emerges due to the incongruity between intra-framework dispersion forces and the geometric limitations of the inorganic building blocks  MOF-5, when chemically functionalized with alkoxy groups, shows remarkable structural flexibility initiated by the formation of Dispersive Energy Donors (DEDs)  Manipulating steric bulk in DEDs allows systematic adjustment of distortion mode and extent in the guest-free phase, ranging from correlated and crystalline distortions (volume contraction up to 3%) to random and non-crystalline distortions (volume contraction up to 17%)  This phenomenon grants exceptional physical characteristics to MOFs with high flexibility, leading to continuous transitions from non-crystalline to crystalline states primarily driven by entropy rather than enthalpy | [48] |
|  |  | a-MOF | NEU-3, NEU-4 | Smart guest-responsive properties attributed to their π Lewis acidic pore surface and presumed framework flexibility | [49] |
|  |  | g-MOF | MOF glasses from carboxylate-based frameworks | Low T_m_ attributed to ligand flexibility, low symmetry, and absence of crystal field stabilization  Paves the way for broader integration of carboxylate-based MOFs into MOF glass chemistry | [35] |
| Functional | Mechanical | a-MOF | ZIF-8 | Average Young’s modulus estimated to be around 5.6 GPa  Poisson’s ratio estimated to be approximately 0.4  Tensile strength of ZIF-8 found to be about 50% greater than its compressive strength | [5] |
|  |  | a-MOF | a-MOF based nanozyme (CA-Cu) | The active site of CA-Cu nanozyme was identified as the coordination between N and copper (N-Cu), with hydroxyl-copper coordination contributing to structural stability rather than catalytic activity. Notably, N-Cu enhances laccase- and catecholase-like activities more effectively than other coordination types | [22] |
|  |  | g-MOF | ZIF-62 | Sample with lowest T_m_, ZIF-62, yielded least dense glass, with curious density increase observed when quenching liquid from 845 K  ZIF-62 sample heated to 845 K without framework decomposition, vitrified upon cooling, exhibited significantly larger elastic modulus compared to sample cooled from just above T_m_ | [30] |
|  |  | a-MOF | ZIF-4 | For the first time, in situ far-infrared spectroscopy was employed to investigate the thermal behavior of a-MOFs  It has been demonstrated that vibrational spectroscopy improves comprehension of how thermal stimuli affect the stability of framework materials  Vibrational spectroscopy was employed to establish a correlation between the susceptibility to thermal-induced amorphization in ZIF-4 and the flexibility of Zn-N tetrahedral moieties  Observed changes in spectral features, which are indicative of amorphization under heat, and identified distinct vibrational changes | [50] |
|  |  | g-MOF | ZIF-8 | Mean hardness and modulus values: H = 0.730 ± 0.136 GPa; E = 5.42 ± 1.10 GPa.  Mechanical properties fall within the range reported for crystalline ZIF-8 and are comparable to other ZIF glasses | [33] |
|  |  | g-MOF | ZIF-8 | Vickers hardness of ZIF-4-bImCl and ZIF-62-bImCl (R=1.0) reduced to ~0.35 GPa vs. ~0.6 GPa in unmodified ZIF glasses  Crack initiation resistance significantly lower: 0.12 N (ZIF-4-bImCl) and 0.38 N (ZIF-62-bImCl) vs. ~2 N in unmodified ZIF-62 | [34] |
|  | Thermal | a-MOF | MOF-5 | Faster weight loss process in amorphous MOF-5 than the crystal counterpart in the temperature range of 400–530 °C  Less weight loss from amorphous MOF-5 (39%) than that from crystal one  Amorphization alters the thermal stability | [2] |
|  |  | g-MOF | ZIF-8 | Clear glass transition for ag(IL@ZIF-8-HT) at ~322–328 °C confirms its glassy nature.  High Tg/Tm ratio (~0.91) indicates exceptional glass-forming ability, surpassing ZIF-62  Minor shift in T_g_ due to higher heating rate and IL–framework interactions  TGA confirms high thermal stability of pristine ZIF-8 with <1% mass loss under LT/HT treatment  IL@ZIF-8 composite exhibits moderated IL decomposition: 20.7% (LT) and 34.4% (HT) | [33] |
|  |  | g-MOF | ZIF-8 | Modified ZIF glasses exhibit T_g_ values of 130–140 °C, ~200 °C lower than unmodified counterparts  Fast and conventional DSC confirm clear glass transitions and successful vitrification.  Higher ΔC_p_ values observed in modified glasses, indicating enhanced configurational change at T_g_.  No decomposition or phase separation during heating confirms thermal stability of modified systems | [34] |
|  | Electrical | a-MOF | PCMOF-17 | Impedance analysis of pelletized powders revealed a proton conduction value exceeding 10^−3^ S cm^−1^ at 25 °C and 40% RH, indicating very high proton conduction for low humidity and moderate temperature  Triaxial impedance analyses on a single crystal confirmed bulk proton conductivity over 10^−3^ S cm^−1^ along two axes, corroborating the data from the pellet  The primary conduction pathways in PCMOF-17 are through the continuous hydrogen bonded pathways in the ab crystallographic plane | [17] |
|  |  | a-MOF | ZIF-8 | Both phases (crystal and amorphous) exhibit similar features in the Density of States (DOS) curves  Energy bands between 18 and 10 eV mainly consist of 1s electronic state of H and 2s electronic states of N and C atoms  Hybridization occurs in the upper portion of the valence band (VB) from 7.5 eV to 2.5 eV  Both ZIF-8 and a_p_ZIF-8 are good insulators despite the crystal-amorphous phase transition  Crystal-amorphous phase transition in ZIF-8 does not significantly alter electronic properties  Despite changes in charge distributions, bond lengths, and bond angles, the electronic structure remains dominated by insulating 2-methylimidazole ligands | [51] |
|  |  | g-MOF | CdTz | Vitrification leads to significant enhancements in proton conductivity and dielectric constant due to disorder and enhanced ligand mobility in glassy structure | [29] |
|  |  | g-MOF | (diethylmethylammonium)_0.35_[Zn(H_2_PO_4_)_2.35_(H_3_PO_4_)_0.65_] | High proton conductivity (13.3 mS cm^-1^) at 120 °C, confirming exclusive proton conduction  A high transport number of the proton (0.94) | [32] |
|  | Optical | a-MOF | ZIF-L | Continuous electron-beam exposure leads to further breakdown of 2-mIm linker molecules in disordered ZIF-L, causing changes in the dielectric function of the material and shifting energies for optical transitions important for device applications | [13] |
|  |  | a-MOF | MIL-100 | Pelleting pressure, temperature, frequency, density, degree of amorphization affect the dielectric properties of the framework  The dielectric properties of MIL-100 changed with different levels of pressure  MOF framework dielectric, optical, and electrical characteristics affected by pore occupancy strongly affects | [52] |
|  |  | g-MOF | CdTz | Augmented mobility of ligands and increased disorder within the glass structure likely contribute to the observed enhancements in conductivity and dielectric constant | [29] |
|  | Magnetic | a-MOF | (Me_2_NH_2_)[Co_3_(Me_2_-NH)_3_(OH)(SDBA)_3_, H_2_SBDA 14 4,40-sulfonyldibenzoic acid | Structural modification through deionization shown as successful route to improve magnetic properties of ionic MOFs  Magnetic susceptibility measurements reveal reversible tuning of magnetic properties during deionization and reionization process | [16] |

**Table S3** Characterization techniques for non-crystalline MOFs.

| **Properties** | **Techniques** | **Information obtained** | **Ref.** |
| --- | --- | --- | --- |
| Chemical environment within | Terahertz/far-infrared spectroscopy (THz/far-IR) | Quasi-localised features | [6, 52-55] |
|  |  | Collective framework dynamic properties |  |
|  |  | Reaction/bonding formation during melting |  |
|  | Solid-state nuclear magnetic resonance spectroscopy (NMR) | Structural characterization | [11] |
|  |  | Element-specific chemical environment within (both bulk and interfacial regions) | [53, 54, 56, 57] |
|  |  | Interactions of linker-linker and host-guest |  |
|  |  | Short-range disorder of the coordination environment of metal in MOFs (upon melting) | [58] |
|  | X-Ray absorption spectroscopies (XAS) | Electronic structure (XANES): valence states/average oxidation state | [53, 59, 60] |
|  |  | Coordination environments (EXAFS): metal’s bond distances of neighbour atoms, chemical bonding information, coordination number |  |
|  | Fourier-transform infrared spectroscopy (FTIR) | Analysing the chemical bonding and coordination environment | [15, 61] |
|  |  | Determination of the functional groupings present |  |
|  |  | Monitoring structural changes and phase transition |  |
|  |  | Study the degradation of the crystal structure |  |
| Nanoscale, microscale, and atomic-scale structure | Small-angle X-ray scattering (SAXS) and wide-angle X-ray scattering (WAXS) | Local density difference (SAXS) | [62] |
|  |  | Confirm the loss/emergence of long-range order (WAXS) | [56, 62, 63] |
|  |  | Crystal distortion, lattice parameters, and preferential orientation (WAXS by refinement) |  |
|  |  | Volume-weighted fraction of the particles (SAXS) | [56, 63] |
|  |  | (In)homogeneity and internal surface roughness (SAXS) | [63] |
|  |  | Particle size/particle coarsening (SAXS) | [54, 63] |
|  | Powder X-ray diffraction (PXRD)/High-pressure PXRD/ Variable-temperature PXRD | Macroscopic phase transition | [6, 10, 31, 54, 64-66] |
|  |  | Change in crystal structure |  |
|  |  | Standard behaviour as a function of pressure, temperature, and lattice compression |  |
|  |  | Crystal structure distortion, lattice parameters, average crystalline domain size and quantities ratios (PXRD pattern refinement of the mixed crystal composites) |  |
|  | X-Ray total scattering and pair distribution functions (PDF) | Short- and mid-range structure for amorphous materials | [31, 67] |
|  | Energy dispersive X-ray analysis | Confirmation of the integrity of the ligand and coordination bond breaking | [12] |
| Microscopic structure | Scanning transmission electron microscopy (STEM) | Phase distribution information for composites (nanometer-level, ADF) | [31, 54, 64, 68] |
|  |  | Elemental distribution (EDS) |  |
|  |  | 3D structural information (reconstruct a tomography surface-rendered ADF/EDS) |  |
|  |  | Spatial-resolute chemical bonding information (bulk and interface, EELS) | [64] |
|  |  | Map the crystalline and amorphous region (SED) | [31, 64] |
|  |  | Split the different amorphous domains (map the changes in the short-range order, ePDF) | [68] |
|  | FIB-SEM | Phase distribution (3D surface-render views) | [69] |
|  |  | Defect volume |  |
|  | In situ high-resolution transmission electron microscopy (HRTEM) | Investigate the path by which a single crystal MOF is destabilized and transformed into MOF-based amorphous nanomaterials | [70] |
|  | Positron Annihilation Lifetime Spectroscopy (PALS) | Presence of porosity | [53, 71, 72] |
|  |  | Pore size distribution |  |
|  | Gas sorption analyser | Langmuir and Brunauer-Emmett-Teller (BET) Surface area | [2] |
|  |  | Pore size distribution |  |
|  |  | Pore volume |  |
|  |  | Gas storage capacity |  |
| Mechanical properties | Constant strain-rate nanoindentation | Young’s modulus and hardness | [73] |
|  | Constant load and hold indentation creep tests and strain-rate jump experiments | Creep resistance | [73] |
|  | Strain rate-dependent uniaxial micropillar compression tests | Plasticity | [73] |
|  | Single-edge pre-cracked beam method | Fracture toughness | [74] |
|  | Micro-indentation and nanoindentation paired with laser scanning microscopy and atomic force microscopy (AFM) | Fracture and deformation behaviours | [75, 76] |
| Thermal behaviours | Thermogravimetric analysis (TGA) | Thermal stability Study, Desolvation, Decomposition | [2, 52, 62, 63] |
|  | Differential scanning calorimetry (DSC) | Melting processes | [28, 77, 78] |
|  |  | Melting temperature (T_m_) |  |
|  |  | Glass transition temperature (T_g_) |  |
|  |  | The amorphization transition confirmation | [66] |
|  |  | Domain distribution | [63, 79] |
| Other properties | Gas sorption and permeation analyser | Gas sorption and permeation capabilities | [69, 80] |
|  | Electron paramagnetic resonance (EPR) measurement | Determination of paramagnetic and diamagnetic | [7] |
|  | Confocal Raman microscopy | Investigate the structural modification of MOF caused by electron-beam irradiation | [13] |
|  | Photoluminescence (PL) spectroscopy |  |  |
|  | Electron diffraction and electron energy-loss spectroscopy (EELS) |  |  |
|  | Becke line method | Refractive index | [61] |
|  | LCR Meter | Measure the Hz to MHz Range | [52] |
|  | Fluorescence spectrophotometer | PL and time-resolved fluorescence emission spectra | [54] |

**Table S4** A summary of the major non-crystalline MOF composites.

| **Composite** | **Non-crystalline MOFs** | **Composition** | | **Synthesis Method** | **Highlights** | **Ref.** |
| --- | --- | --- | --- | --- | --- | --- |
|  |  | **MOF** | **Compositing material** |  |  |  |
| 2D Cd^2+^ CP crystal | a-MOF | CdTz | CdTz [Cd(H_2_PO_4_)_2_(HTr)_2_] (Tr 14 1,2,4-triazole) | Solvent-free ball milling | Unchanged local structural configuration around Cd^2+^ centres  Improved proton conductivity and dielectric constant with the presence of a distorted glass structure | [29] |
| Bimetallic Co-Fe MOF glass | g-MOF | ZIF-62(Co) | ZIF-62(Co) with adsorbed Fe(acac)_3_ | Mixing + Melt quenching | Improved oxygen evolution reaction performance  Enhanced charge transfer efficiency and catalytic activity | [53] |
| MOF crystal-glass composites | g-MOF (ZIF-62) | MIL-53 (crystalline MOF) | Dispersing crystalline MOFs within a MOF-glass matrix  (crystalline-MOF)_x_(gZIF-62)_y_ | ball milling + Thermal treatment (under Ar gas) + Cooling | The glass matrix stabilises the phase transition of flexible MIL-53, maintaining its open pore structure  Significant improvement in gas adsorption at room temperature | [64] |
| Porous composite | g-MOF (ZIF-62) | UiO-66 |  |  | Partial decomposition observed at 450 °C after extended heating  Poorer retention of structure compared to MIL-53 |  |
| MOF/polymer composite | g-MOF | ZIF-62 | 6FDA-DAM polyimide-based composite membrane | Melt quenching  polymer (suspension)  membrane casting | Dynamically under-coordinated metal nodes and organic ligands in liquid phase conformations bond with polymeric matrices, rigidifying polymer chains  Enhanced Mechanical rigidity  Membrane molecular separation performances show in situ melting membrane selectivity  ZIF glass allows for shaping into macroscopic structures in a molten liquid state | [69] |
| Inorganic–MOF glass  composites | Phosphate glass – ZIF composite | ZIF-62 | ZIF-62 + Inorganic glass (phosphate glass) | Ball milling both ZIF-62 and inorganic glass | Induces greater mechanical flexibility in the composite relative to that of the inorganic glass | [79] |
| MOF glass membrane | g-MOF | ZIF-62 | ZIF-62 + alumina support | Melt-quenching treatment of an in situ solvothermal synthesized polycrystalline MOF membrane on a porous ceramic alumina support | High separation performance and selectivity for various gas mixtures, including H_2_/CH_4_, CO_2_/N_2_, and CO_2_/CH_4_  superior long-term stability | [80] |
| a-MOF-NC | a-MOF | MIL-88B | MIL-88B  25 mM 2-methylimidazole solution  25 mM Co(NO_3_)_2_ | Mixing f MIL-88B and 25 mM 2-methylimidazole solution (10 min) => aMIL-88B was generated  aMIL-88B+ 3 mL of 25 mM Co(NO_3_)_2_ solution + without stirring (under air atmosphere) => Collection, washing, drying | The formation of a core-shell structure highlighted a significant efficacy in oxygen evolution reaction, with a minimal overpotential of 249 mV at 10.0 mA cm^-2^ and Tafel slope of 39.5 mV dec^-1^ | [81] |
| Ultra-thin 2D MOF membranes | Zn_2_(bim)_4_ | Zn_2_(bim)_4_ | Zn_2_(bim)_4_ on a-alumina | Procedure for MOF: soft-physical process-wet ball- milled at very low speed followed  by exfoliation in a volatile solvent with the aid of ultrasonication | A Zn_2_(bim)_4_ MSN membrane demonstrated excellent stability after more than 400 hours of testing, including temperature cycles and exposure to an equimolar H_2_/CO_2_ feed with ~4 mol % steam at 150 °C | [82] |
| g MOF based quasi-solid-state electrolyte | g-MOF | ZIF-4 | ZIF-4 combined with lithium bis(trifluoromethane sulfonyl)imide (LiTFSI) + PTFE as quasi-solid-state electrolytes | Grind mixture + Roll into film | Higher ion conductivity compared to crystalline ZIF-4-based QSSEs (LCZ)  High Li contents and large plasticizer amounts  Grain boundary-free and isotropic properties of glassy ZIF-4 facilitate ion conduction, leading to homogeneous ion flux and suppression of Li dendrites | [83] |
| MOF/polymer composite | g-MOF | ZIF-62 | ZIF-62 MOF glass and cellulose acetate | Ball milling (g-MOF) + Solvent casting technique | Significantly improve CO_2_ permeability and CO_2_/CH_4_ ideal selectivity  High CO_2_ plasticization pressure (26 bar) | [84] |
| aMIL-53(Al)/Co-LDH heterojunction | a-MOF | cMIL-53(Al) | aMIL-53(Al)/Co-LDH heterojunction | Prepare cMIL-53(Al) methanol suspension + add Cobalt Nitrate (Co(NO_3_)_2_) + Reaction and product formation + Collection + Washing + Drying | A high specific capacitance of 773.2 F g^-1^ at 1 A g^-1^ is achieved, significantly higher than original Co-LDH and aMIL-53(Al)  The asymmetric supercapacitor (ASC) exhibits a maximum power density of 7978.8 W kg^-1^ at a current density of 9.8 Wh kg^-1^ with good durability.  The aMIL-53(Al)/Co-LDH heterojunction demonstrates lower overpotential and a smaller Tafel slope in oxygen evolution reaction (OER), indicating improved electrochemical activity. | [85] |
| Mesoporous composites | a-MOF | UiO-66 | MIL-53 | Solvent-thermal synthesis, coating UiO-66 onto MIL-53 surface | The amorphous shell layer enhances catalyst adsorption.  MIL-53@UiO-66 composites with equalizing Fermi level facilitate photon absorption and electron transfer | [86] |
| MOF crystal–glass composites | g-MOF | ZIF-62 | MIL-118(Al)  (or UL-MOF-1) | Ball-milling + Pressing + Heating (under Ar atmosphere) + Colling | Composite performance is improved by enhancing matrix porosity or overcoming percolation threshold  gZIF-62 matrix prevents diffusion of molecules larger than C_3_H_6_ | [87] |
| MOF-glass | g-MOF | ZIF-67, ZIF-62,  ZIF-8 | ZIF-8/ZIF-62 and ZIF-67/ZIF-62 | Flux melting + Quenching | Enhancement of accessible porosity to guest molecules  Flux melted glass exhibits short-range ordering akin to crystalline ZIF-62 and a continuous random network similar to amorphous SiO_2_, with accessible porosity | [88] |
| Hybrid ZIF-8/ZIF-62 glass membrane | g-MOF | ZIF-8 | Alumina support | In situ solvothermal + Melt-quenching | Enhanced both the adsorption capacity and the ideal C_3_H_6_/C_3_H_8_ selectivity | [89] |
| TIF-4 MOF glass membrane | g-MOF | TIF-4 | Alumina support | In situ solvothermal + Melt-quenching | A lowered melting point facilitates the fabrication of MOF glass-based hybrid materials  Long-term stability up to 10 months  High CO_2_ selective with CO_2_/N_2_ and CO_2_/CH_4_ separation | [90] |
| Metal inorganic–organic complex glass and fiber | agM-P-dmbIm  MIOC | Zn-azolate; Cd-azolate; ZnCl2(HbIm)_2_ | Inorganic phosphors (yellow, red, blue, green); organic dyes | Mixing in epoxy resin/mixing + Melt quenching | The glass minimizes the occurrence of non-radiative transitions of triplet excitons. | [91, 92] |
| MOF-photonic glasses |  |  |  |  | Direct ultralong room-temperature phosphorescence output with a notable PL quantum yield of up to 75% |  |
| Advanced nanophotonics | a-MOF | Metalcarboxylateazolate (Cd or Zn based) | MOF is converted into core–shell composites with metal oxide core and organic shell | Ultrafast melting | The structural elements of MOF glass can be reoriented and reassembled into new structures | [93] |
| Solid state electrolyte | a-ZIF-62 | ZIF-62 | Na_3_Zr_2_Si_2_PO_12_ structured NASICON pellets + ZIF-62 | Spin coating + Melt quenching | Interfacial Na+ transport inhomogeneity and heterogeneous Na stripping/plating processes  Improves interfacial compatibility with Na-anode and homogenizes e^−^/Na^+^ transport kinetics, leading to spatially even Na nucleation and transition of Na deposition behaviour from dendrites to lateral flat-shape growth tendency | [94] |
| CP mixed glasses | Homogeneous mixed glasses | ZnPIm + ZnPBIm | ZnPIm + ZnPBIm | Ball milling + Melt quenching | Homogeneously mixed glass  Reversible solid-to liquid phase transition | [95] |
| Proton-conductive CP glass | CP Glasses | Zn-phosphate; Zn-phosphateazolate | Electrode (carbon fibre; Pt/C carbon paper) | Melt quenching | Acts as anhydrous solid-state electrolyte for battery and fuel cell | [96, 97] |
| CP glasses | CP Glasses |  |  |  | No discontinuities or flaws at MOF glass/electrode interface |  |
| MOF-glass anode | (Co(Im)_1.75_(bIm)_0.25_) | Co-ZIF-62 | Carbon black + Polymer binder | Mixing + Melt quenching | Additional sites for Li^+^ diffusion because of distortion and local breakage of the Co-N coordination bonds | [98] |
| MOF nanocrystal coatings | a-MOF | Co-ZIF-4; Co-ZIF-62 | MOFs + Carbon cloth substrate | Ball milling + Coating + Melt quenching | Catalytic active sites  Good interfacial contact with carbon cloth electrode due to access to the liquid state | [99] |

**Table S5** A summary of the major practical applications of non-crystalline MOFs.

| **Application** | **Non-crystalline MOF** | **Composition** | **Synthesis Method** | **Highlights** | **Ref.** |
| --- | --- | --- | --- | --- | --- |
| Gas adsorption | a-MOF | ZIF-8 | Pressure-induced amorphization | Systematic decreases in total uptake.  Increases in uptake at low pressure (P/P_0_ < 10^-4^) with rising treatment pressure impacting broader sorption properties | [1] |
|  | a-MOF | NEU-2 | Dissolution of Iron (II) acetate anhydrous + Preparation of H_2_BPDI solution + Solvothermal treatment | The NEU-2 material contains meso- and macro-pores impregnated with polyethyleneimine (PEI), while the micropores remain unobstructed to facilitate CO_2_ diffusion  The structured crystalline form of MOFs is not mandatory for gas absorption. The composite demonstrates using amorphous MOFs with hierarchical porosity as a viable option for CO_2_-capture purposes  CO_2_ access to amine sites increases with rising amine loadings, avoiding pore blockage and diffusion resistance  CO_2_ uptake capacity significantly decreases as temperature increases from 25 °C to 75 °C.  Practicality demonstrated for using low-temperature sorption-supported amine systems for CO_2_ capture | [25] |
|  | a-MOF | UIO-66-NH_2_ | Disperse amorphous ACU particles in PVA solution + Uniform coating + Drying | High IAST CH_4_/N_2_ selectivity  Good alkaline stability  Satisfactory polymer compatibility | [39] |
|  | a-MOF | Cu-BTC | Pressure-induced amorphization | Cu-BTC has a high porosity and a large surface area, making it suitable for gas adsorption | [40] |
|  | a-MOF | UPJS-13  UPJS-14 | Solvent mixture + Heating and reaction (crystal formation) + Isolation of crystals + Drying | Composites were evaluated for high-pressure adsorption of CO_2_ and CH_4_. The compounds were tested at 30°C and 20 bar, UPJS-13 (FD) exhibited maximal adsorption capacities of 30.01 wt. % CO_2_ and 4.84 wt. % CH4.UPJS-14 (FD) demonstrated maximal adsorption capacities of 24.56 wt. % CO_2_ and 6.38 wt. % CH_4_ under the same conditions | [44] |
|  | a-MOF | NEU-4 | Liquid-liquid interface synthesis  method | Showed promising characteristics that can be further investigated for separations the petrochemical industry. For example, ultrahigh benzene adsorption, recognition capability, selectivity for benzene over analogues, and high stability and regenerability | [49] |
|  | g-MOF | (MIL-53)_x_(agZIF-62)_1–x_ | Mechanically induced amorphization | Greater CO_2_ adsorption capability of MOF-CGC than that of pure MIL-53-np | [31] |
|  | g-MOF | Crystalline: MIL-53  Non-Crystalline: ZIF-62 glass matrix | Dispersing crystalline MOFs within a MOF-glass matrix | A significant enhancement of CO_2_ adsorption capacity  The stabilization of MIL-53's structure at room temperature results in a significant improvement in gas adsorption | [64] |
| Gas separation | a-MOF | NEU-2 | Dissolving + mixing the solutions + Teflon-lined Parr reactor (100 °C, 24 h) | Mesopores and macropores are impregnated with PEI (polyethylenimine) to enhance amine-based CO_2_ adsorption, while micropores remain open for CO_2_ diffusion.  Amorphous NEU-2 outperforms crystalline NEU-1c in CO_2_ capture, proving that crystallinity is not essential for gas adsorption in MOFs | [25] |
|  | a-MOF | ZIF-69, ZIF-mnIm | Pressure-induced amorphization | ZIF-69 and ZIF-mnIm retained their previous pore spaces (and, as such, held I_2_) up to 200 ℃, presenting a possible method to trap gas pollutants irreversibly | [42] |
|  | a-MOF | UiO-66-NH_2_ | Dispersing amorphous SU-1 nanoparticles + Preparing coating solution + Stirring coating solution + Allowing the coating solution to stand + Coating+ Drying process | Small-sized aAMOF particles have enhanced alkaline stability, higher hydrophilicity, and superior CO_2_/N_2_ adsorption selectivity compared to crystal forms.  Polyethyleneimine (PEI) is used as an inducer to create small-sized UiO-66-NH_2_ particles (SU-1), improving membrane-forming properties  The resulting MMMs exhibit optimal CO_2_ separation performance at an ASU-1 loading of 28.5 wt%, with a CO_2_/N_2_ selectivity of 71.33 at 1.5 bar and a CO_2_ permeance of 721 GPU | [100] |
|  | g-MOF | ZIF-62 | 50 wt% ZIF-62-L/PEI/MPSf | The abundance of open metal sites in ZIF-62-L improves CH_4_/N_2_ adsorption selectivity, enhancing interaction with CH_4_  50 wt% ZIF-62-L/PEI membrane shows 505% higher CH_4_ permeance and 230% higher CH_4_/N_2_ selectivity than pure PEI/MPSf composite membrane  This study highlights the potential of low-crystallinity MOFs in developing high-performance MMMs and underscores the role of mechanochemical synthesis in obtaining ideal filler materials | [9] |
|  | g-MOF | ZIF-62 | Melt-quenching treatment | The molten ZIF-62 phase infiltrated the nanopores of the support, eliminating the formation of intracrystalline defects in the resulting glass membrane  The polycrystalline film completely transforms into an isotropic glass film without grain boundaries through a melting and quenching process  Upon cooling to ambient temperature, the ZIF-62 liquid transitions into a glassy state, forming a ZIF-62 glass film  The MOF glass membrane exhibited remarkable separation factors for H_2_/CH_4_, CO_2_/N_2_, and CO_2_/CH_4_ mixtures, measuring 50.7, 34.5, and 36.6, respectively | [80] |
|  | g-MOF | Zn-ZIF-62; Zn-TIF-4; hybridZn-ZIF-8/ZIF-62 // Alumina membrane substrate | Solvothermal synthesis + Melt quenching | Enhanced Performance (H_2_/CH_4_, CO_2_/N_2_, and CO_2_/CH_4_, C_3_H_6_/C_3_H_8_) | [89, 90] |
|  | g-MOF | Zn-ZIF-62; M-phosphateazolate (M = Zn, Cd, Cu and Mn)/Polymer membrane matrix (6FDA-DAM; PTFE) | Stirrer mixing + Casting + melt quenching + Hot-pressing | Improved gas selectivity (CO_2_/N_2_)  No significant change permeability | [69, 91] |
|  | g-MOF | ZIF-62 | Self-supporting glass MOF membrane | High permeability and selectivity for different gases:  H_2_ permeability: 509.23 barrer  CO_2_ permeability: 146.07 barrer  H_2_/CH_4_ selectivity: 223.47  CO_2_/N_2_ selectivity: 42.37  CO_2_/CH_4_ selectivity: 64.10  H_2_/N_2_ selectivity: 147.71  H_2_/CO_2_ selectivity: 3.49 | [101] |
| Molecule and ion trapping | a-MOF | aMOC-1 and aMOC-2 | Preparation of reaction mixture+ Reaction+ Isolation of product+ Drying | Presence of large cavities  High positive charges  Rapid kinetics in carcinogenic chromate (CrO_4_^2–^ and Cr_2_O_7_ ^2–^) and ReO_4_^–^ (a surrogate anion of radioactive isotope TcO_4_^–^) adsorption | [102] |
|  | a-MOF | ZIF-8 | Pressure-induced amorphization | The local structure of trapped I_2_ remained unchanged after the amorphization of the framework, occurring under the same conditions for both vacant and guest-loaded frameworks | [103] |
| Ion transport | a-MOF | [H_2_Im][Cu(H_2_PO_4_)_2_Cl] | Ball milling | For the 1D network, heating disrupts hydrogen bonding and leads to amorphization  The increase in the number of proton carriers means that the anhydrous proton conductivity of amorphous [H_2_Im][Cu(H_2_PO_4_)2Cl] is >10^-2^ S cm^-1^ at 130 °C | [104] |
|  | g-MOF | [Cd(H_2_PO_4_)_2_(HTr)_2_] | Melt doping | Strong acid doping in CP results in minimal structural changes but enhances anhydrous proton conductivity by increasing the number of charge carriers and creating local defects to improve proton transport  Photo acid doping in CP enables external control over the number and mobility of protons, with conductivity being switched reversibly by light irradiation | [105] |
| Supercapacitor | a-MOF | UiO-66 | Direct synthesis | The specific capacitance of a-UiO-66 reached 920 F g⁻¹ at 10 mV s⁻¹, significantly higher than the 452 F g⁻¹ observed for crystalline UiO-66  The specific capacitance of a-UiO-66 remained at 610 F g⁻¹ even after 5000 cycles, indicating excellent cycling stability | [106] |
| Battery | a-MOF | MIL-88B | Chemical treatment-induced amorphization | aMIL-88B exhibits increased conductivity, greater exposure to active sites, and higher adsorptive/catalytic activity toward polysulfides  Utilizing aMIL-88B as a separator modifier effectively hinders polysulfide shuttling and accelerates reaction kinetics in Lithium-Sulfur batteries  Significant enhancement was observed in the electrochemical efficacy of lithium-sulfur batteries  Li-S cells based on aMIL-88B-modified separator demonstrate remarkable cycling performance, with a capacity of 740 mAh g^-1^ after 500 cycles and great rate capability up to 5 C | [107] |
|  | a-MOF | NPO-OH-T microspheres | Preparation of Ni(OH)_2_ microspheres by hydrolyzing the Ni-MOF precursors.  The Ni(OH)_2_ template and KH_2_PO_4_ are utilized as phosphorous sources.  NPO-OH-T microspheres are synthesised through a hydrothermal method | The spherical morphology of the MOF template remains intact during treatment, resulting in nickel phosphates with rich porosity, ultrahigh-specific surface areas, and a robust structure  The rich porosity enhances the migration of electrolyte ions, while the high specific surface area provides numerous active sites | [108] |
|  | l-MOF | Zn-ZIF-62/Solid-state electrolyte (NASICON) | Spin coating + melt quenching | Enhanced cycling stability in Na metal battery due to homogeneous Na^+^/e^-^ transport kinetics | [94] |
|  | g-MOF | ZIF-4 | Glassy ZIF-4 combined with lithium bis(trifluoromethanesulfonyl)imide (LiTFSI) | A Remarkable ion conductivity despite low Li and solvent content.  The grain boundary-free and isotropic properties of glassy ZIF-4 enable homogeneous ion flux, suppressing Li dendrites | [83] |
|  | g-MOF | ZnPIm + ZnPBIm/Mixed composite of two MOF glasses | Ball milling + melt quenching | Ion conductivity is controlled via a change in ratio of glass components | [95] |
|  | g-MOF | Zn-phosphate; Zn-phosphateazolate/ Electrode (carbon fibre; Pt/C carbon paper) | Melt quenching | Enhanced charge transfer kinetics at electrode-electrolyte interface | [96, 97] |
|  | g-MOF | Co-ZIF-62/ Carbon black + polymer binder | Mixing + melt quenching | Higher storage capacity  More cycling stability  High-rate performance | [98] |
| Reversible transitions | a-MOF | Gold(I)-thiophenolate | Thermally-induced solid-state amorphous-to-crystalline isomerization | A solvent-free amorphous-to-crystalline transformation upon heating | [109] |
|  | a-MOF | Cu[Cu(pdt)_2_] (pdt^2-^ = 2,3-pyrazinedithiolate) | Heating | Anomalously higher conductivity than the crystalline phase  Higher conductivity attributed to the generation of new Cu–S bonds upon structural collapse | [110] |
| Catalysis and electrocatalyst | a-MOF | 2D bimetallic Fe_1_Ni_2_(BDC-NH_2_) MOF | Direct synthesis | The electrocatalyst exhibited a low overpotential of 228 mV at a current density of 10 mA cm^-^² and a Tafel slope of 30.3 mV dec^-^¹  Good operational stability in continuous electrolysis | [21] |
|  | a-MOF | CA-Cu nanozyme | Solvothermal | High catalytic activity, stability, and compatibility  CA–Cu nanozyme enables efficient phenol degradation and dopamine detection | [22] |
|  | g-MOF | Co-ZIF-4; Co-ZIF-62 // Fe containing components, deposited on carbon cloth substrate | Ball milling (coating) + melt quenching | High catalytic efficiency  Overpotential for oxygen evolution reaction  Reduced charge transfer resistance at the electrode interface | [53] |
| Drug delivery | a-MOF | UiO-66 | Ball milling | Achieve prolonged controlled release via MOF dissolution and drug diffusion in the porous media  Low toxicity, and high stability  The delivery time of a model anti-cancer drug increased significantly  The delivery time increased from 2 to 30 days compared to the crystallized MOF | [8] |
|  | a-MOF | MIL-100(Fe) | Hydrothermal synthesis | In disordered and amorphous MNF2, interactions are lower, allowing for easy drug release  Due to the weaker interactions between the drug and the carrier, the loaded DOX was easily released from amorphous MNF2  Among the three MIL-100(Fe) carriers, MNF2 exhibits the highest release percentage  Note:  MF (presence of HF)  MNF1 (absence of HF and lower water content)  MNF2 (absence of HF) | [43] |
|  | a-MOF | CAU-7 | Mechanical and thermal amorphization processes | Composite utilized as a drug delivery system (DDS) for two cancer drugs: sodium dichloroacetate (DCA) and α-cyano-4-hydroxycinnamic acid (α-CHC)  Mechanical and thermal amorphization processes attempted to delay drug delivery, resulting in an outstanding 32% slower release of α-CHC from thermally treated CAU-7  A gradual drug release process was achieved, with a release time of 17 days for DCA and 31 days for α-CHC  In vitro studies, endocytosis inhibitors, confocal microscopy, and fluorescence-activated cell sorting demonstrated successful internalization of CAU-7 by cancer cells, partially avoiding lysosome degradation  CAU-7 loaded with DCA or α-CHC exhibited higher therapeutic efficiency than the free drug approach, suggesting its potential as a promising option for biomedical applications.  The thermal amorphization process of CAU-7 resulted in a 32% slower release of α-CHC, showcasing controllable drug release kinetics | [111] |
|  | g-MOF | Zr-based family of MOFs | Mechanical milling-induced amorphization | Biocompatibility within evaluated concentration range  Since PO_4_^3⁻^ is an electrolyte in the body, MOFs are expected to biodegrade without accumulating in cells  Improved water stabilities were observed in growth media, possibly due to the adsorption of proteins forming a 'protein corona' on MOF particles  No significant difference in cell viability was observed when treated with empty and loaded Zr–L1 to Zr–L5  Zr–L6 shows a significant difference in viability at 1 mg mL⁻¹ concentration, suggesting different internalization mechanisms for each MOF  The therapeutic effect of a-CHC loaded in each Zr-based MOF was assessed  Enhanced effect of drug observed when loaded into Zr–L6  Note:  Zr–L1 to Zr–L4 were synthesized using terephthalic acid (BDC), 2-bromoterephthalic acid (Br-BDC), 2-nitroterephthalic acid (NO_2_-BDC), and 2-aminoterephthalic acid (NH_2_-BDC), respectively, while Zr–L5 and Zr–L6 were prepared using naphthalene-2,6-dicarboxylic acid (NDC) and 4,4′-biphenyldicarboxylic acid (BPDC, 352 mg, 1.63 mmol), with all ligands dissolved alongside benzoic acid and HCl in DMF for MOF formation. | [10] |
| Optics | a-MOF | A serious of M-BTC  (M: Fe^3+^, Co^3+^, Co^2+^, Ni^2+^, and Cu^2+^) | Irradiation-induced amorphization | Relationship between initial and resulting structures, and stability of obtained phase and its photoluminescence, aid in designing new amorphous MOF-based optical nanomaterials | [12] |
|  | a-MOF | Bio-MOF-100 | Solvothermal | The topological distortion network allows for high-quality single-mode lasing oscillations | [47] |
|  | a-MOF | Metalcarboxylateazolate (Cd or Zn based)/ MOF is converted into core-shell composites with metal oxide core and organic shell | Ultrafast melting | Improved nonlinear optical properties | [93] |
|  | g-MOF | Zn-ZIF-62/CsPbI_3_ perovskites | Ball milling + melt quenching | MOF glass serves as a matrix for LHPs, effectively stabilizing nonequilibrium perovskite phases through interfacial interactions  Interactions also passivate LHP surface defects, yielding bright, narrow-band PL for creating white light-emitting diodes (LEDs)  High stability against immersion in water and organic solvents, as well as exposure to heat, light, air, and ambient humidity | [54] |
|  | g-MOF | Zn-azolate; Cd-azolate; ZnCl_2_(HbIm)_2_/ Inorganic phosphors (yellow, red, blue, green); organic dyes | Mixing in epoxy resin/mixing + melt quenching | Development of an all-photonic memory system based on 1D MOF glass fibre | [112] |
|  | g-MOF | ZIF-62 | Melt quenching | The strong nonlinear-optical (NLO) response of ZIF-62 is promising for future implementations in the photonics fields for sensing and optical modulation in the near-infrared (NIR) spectral region. | [113] |
| Full Cell | a-MOF | a-NiCo-MOFNWs | Two-step pyrolysis - Heat-Induced Interconnection (HII) and Heat-Induced Phosphorization (HIP) | Long-term durability was demonstrated by over 1000 cyclic voltammetry (CV) measurements  Possessing charge transport path  Abundant exposed active sites  Accelerated electron transport  Boosts methanol oxidation reactionreaction kinetics | [24] |
|  | g-MOF | diethylmethylammonium 0.35[Zn(H_2_PO_4_)_2.35_(H_3_PO_4_)_0.65_] | Direct synthesis of glasses | Proton-conducting and viscoelastic properties demonstrate superior performance in an H_2_/O_2_ fuel cell (0.15 W cm^-2^) | [32] |
| Detector | a-MOF | a-MOF based nanozyme (CA-Cu) | Direct synthesis | Due to its high catalytic activity, stability, and broad substrate universality, the CA-Cu nanozyme demonstrated successful degradation of chlorophenol and diphenol and effective detection of dopamine  The CA-Cu nanozyme exhibits superior catalytic activity relative to natural enzymes Demonstrated excellent stability and recyclability under diverse conditions, including different pH levels (6 to 10), temperatures, long-term storage, and high salt concentrations | [22] |
|  | a-MOF | amEu-NH_2_BDC | Solvothermal process | Composite is utilized as a fluorescence probe for determining water content in ethanol  Exhibited dual-wavelength fluorescence (FL) with ligand (NH2BDC)-centered blue luminescence at 430 nm and Eu-centered red luminescence at 615 nm  With increasing water content in ethanol, the emission intensity at 430 nm significantly increases, while that at 615 nm decreases under a single excitation at 270 nm  The ratio of emission intensity at 430 nm and 615 nm (I430/I615) allows for a wide concentration range detection of water in ethanol, spanning from 1% to 99.5%, with a low detection limit of 0.024% (S/N = 3)  In the presence of water in ethanol, the fluorescence color of amEu-NH2BDC changes from red to blue under 365 nm UV light, enabling visual detection of water  Rapid response, excellent stability, high sensitivity, and selectivity allow the water content in white spirit samples to be successfully measured and monitored | [114] |

**References**

1. K. W. Chapman, G. J. Halder, P. J. Chupas, Pressure-induced amorphization and porosity modification in a metal− organic framework, *J. Am. Chem. Soc.*, **2009**, 131, 17546-17547.

2. Y. H. Hu, L. Zhang, Amorphization of metal-organic framework MOF-5 at unusually low applied pressure, *Phys. Rev. B.*, **2010**, 81, 174103.

3. A. S. Poryvaev, D. M. Polyukhov, M. V. Fedin, Mitigation of Pressure-Induced Amorphization in Metal-Organic Framework ZIF-8 upon EPR control, *ACS Appl. Mater. Interfaces*, **2020**, 12, 16655–16661.

4. A. U. Ortiz, A. Boutin, A. H. Fuchs, F.-X. Coudert, Investigating the Pressure-Induced Amorphization of Zeolitic Imidazolate Framework ZIF-8: Mechanical Instability Due to Shear Mode Softening, *J. Phys. Chem. Lett.*, **2013**, 4, 1861-1865.

5. M. Erkartal, M. Durandurdu, Pressure-induced amorphization, mechanical and electronic properties of zeolitic imidazolate framework (ZIF-8), *Mater. Chem. Phys.*, **2020**, 240, 122222.

6. A. Celeste, A. Paolone, J.-P. Itié, F. Borondics, B. Joseph, O. Grad, G. Blanita, C. Zlotea, F. Capitani, Mesoporous Metal–Organic Framework MIL-101 at High Pressure, *J. Am. Chem. Soc.*, **2020**, 142, 15012-15019.

7. P. Serra-Crespo, A. Dikhtiarenko, E. Stavitski, J. Juan-Alcañiz, F. Kapteijn, F.-X. Coudert, J. Gascon, Experimental evidence of negative linear compressibility in the MIL-53 metal–organic framework family, *CrystEngComm.*, **2015**, 17, 276-280.

8. C. Orellana-Tavra, E. F. Baxter, T. Tian, T. D. Bennett, N. K. Slater, A. K. Cheetham, D. Fairen-Jimenez, Amorphous metal–organic frameworks for drug delivery, *Chem. Commun.*, **2015**, 51, 13878-13881.

9. C. Ma, Z. Yang, X. Guo, Z. Qiao, C. Zhong, Size-reduced low-crystallinity ZIF-62 for the preparation of mixed-matrix membranes for CH_4_/N_2_ separation, *J. Membr. Sci.*, **2022**, 663, 121069.

10. C. Orellana-Tavra, R. J. Marshall, E. F. Baxter, I. A. Lázaro, A. Tao, A. K. Cheetham, R. S. Forgan, D. Fairen-Jimenez, Drug delivery and controlled release from biocompatible metal–organic frameworks using mechanical amorphization, *J. Mater. Chem. B.*, **2016**, 4, 7697-7707.

11. T. D. Bennett, T. K. Todorova, E. F. Baxter, D. G. Reid, C. Gervais, B. Bueken, B. Van de Voorde, D. De Vos, D. A. Keen, C. Mellot-Draznieks, Connecting defects and amorphization in UiO-66 and MIL-140 metal-organic frameworks: a combined experimental and computational study. *Phys. Chem. Chem. Phys.,* **2016**, 18, 2192-2201.

12. Y. A. Mezenov, S. Bruyere, A. Krasilin, E. Khrapova, S. V. Bachinin, P. V. Alekseevskiy, S. Shipiloskikh, P. Boulet, S. Hupont, A. Nomine, B. Vigolo, A. S. Novikov, T. Belmonte, V. A. Milichko, Insights into Solid-To-Solid Transformation of MOF Amorphous Phases, *Inorg. Chem.*, **2022**, 61, 13992-14003.

13. S. Ghosh, H. Yun, P. Kumar, S. Conrad, M. Tsapatsis, K. A. Mkhoyan, Two distinct stages of structural modification of ZIF-L MOF under electron-beam irradiation, *Chem. Mater.*, **2021**, 33, 5681-5689.

14. R. N. Widmer, G. I. Lampronti, N. Casati, S. Farsang, T. D. Bennett, S. A. T. Redfern, X-ray radiation-induced amorphization of metal–organic frameworks, *Phys. Chem. Chem. Phys.*, **2019**, 21, 12389-12395.

15. A. López‐Olvera, H. Montes‐Andrés, E. Martínez‐Ahumada, V. B. López‐Cervantes, R. D. Martínez‐Serrano, E. González‐Zamora, A. Martínez, P. Leo, C. Martos, I. A. Ibarra, Understanding the Mechanism of Amorphization for Co‐URJC‐5, *Eur. J. Inorg. Chem.*, **2021**, 2021, 4458-4462.

16. C.-B. Tian, Y.-H. Han, Z.-Z. He, S.-W. Du, Magnetic Tuning of an Anionic CoII-MOF through Deionization of the Framework: Spin-Canting, Spin-Flop, and Easy-Plane Magnetic Anisotropy, *Chem. Eur. J.*, **2017**, 23, 767-772.

17. B. Joarder, J.-B. Lin, Z. Romero, G. K. Shimizu, Single crystal proton conduction study of a metal organic framework of modest water stability, *J. Am. Chem. Soc.*, **2017**, 139, 7176-7179.

18. X. Zhang, L. Song, F. Bi, D. Zhang, Y. Wang, L. Cui, Catalytic oxidation of toluene using a facile synthesized Ag nanoparticle supported on UiO-66 derivative, *J. Colloid Interface Sci.*, **2020**, 571, 38-47.

19. M. R. Lohe, M. Rose, S. Kaskel, Metal–organic framework (MOF) aerogels with high micro-and macroporosity, *Chem. Commun*., **2009**, 40, 6056-6058.

20. Z. Xin, X. Chen, Q. Wang, Q. Chen, Q. Zhang, Nanopolyhedrons and mesoporous supra-structures of Zeolitic Imidazolate framework with high adsorption performance, *Microporous Mesoporous Mater.*, **2013**, 169, 218-221.

21. J. Ma, X. Bai, W. He, S. Wang, L. Li, H. Chen, T. Wang, X. Zhang, Y. Li, L. Zhang, Amorphous FeNi-bimetallic infinite coordination polymers as advanced electrocatalysts for the oxygen evolution reaction, *Chem. Commun.*, **2019**, 55, 12567-12570..

22. J. Wang, R. Huang, W. Qi, R. Su, Z. He, Preparation of amorphous MOF based biomimetic nanozyme with high laccase-and catecholase-like activity for the degradation and detection of phenolic compounds, *Chem. Eng J.*, **2022**, 434, 134677.

23. W. Zhang, Y. Liu, H. S. Jeppesen, N. Pinna, Stöber method to amorphous metal-organic frameworks and coordination polymers. *Nat. Commun.,* **2024**, 15, 5463.

24. M. Han, Z. Zhao, X. Zhang, P. Wang, L. Xing, D. Jia, L. Wang, X. Chen, H. Gao, G. Wang, Phosphorus-Doped directly interconnected networks of amorphous Metal-Organic framework nanowires for efficient methanol oxidation, *J. Colloid Interface Sci.*, **2023**, 641, 675-684.

25. J. Fonseca, S. Choi, Synthesis of a novel amorphous metal organic framework with hierarchical porosity for adsorptive gas separation. *Microporous Mesoporous Mater.,* **2021**, 310, 110600.

26. R. Gaillac, P. Pullumbi, K. A. Beyer, K. W. Chapman, D. A. Keen, T. D. Bennett, F.-X. Coudert, Liquid metal–organic frameworks, *Nat. Mater.*, **2017**, 16, 1149-1154.

27. J. Yan, C. Gao, S. Qi, Z. Jiang, L. R. Jensen, H. Zhan, Y. Zhang, Y. Yue, Encapsulation of nano-Si into MOF glass to enhance lithium-ion battery anode performances, *Nano Energy.*, **2022**, 103, 107779.

28. A. Qiao, T. D. Bennett, H. Tao, A. Krajnc, G. Mali, C. M. Doherty, A. W. Thornton, J. C. Mauro, G. N. Greaves, Y. Yue, A metal-organic framework with ultrahigh glass-forming ability, *Sci. Adv.*, **2018**, 4, eaao6827.

29. W. Chen, S. Horike, D. Umeyama, N. Ogiwara, T. Itakura, C. Tassel, Y. Goto, H. Kageyama, S. Kitagawa, Glass Formation of a Coordination Polymer Crystal for Enhanced Proton Conductivity and Material Flexibility, *Angew. Chem. Int. Ed.*, **2016**, 55, 5195-5200.

30. T. D. Bennett, Y. Yue, P. Li, A. Qiao, H. Tao, N. G. Greaves, T. Richards, G. I. Lampronti, S. A. Redfern, F. d. r. Blanc, Melt-quenched glasses of metal–organic frameworks, *J. Am. Chem. Soc.*, **2016**, 138, 3484-3492.

31. C. W. Ashling, D. N. Johnstone, R. N. Widmer, J. Hou, S. M. Collins, A. F. Sapnik, A. M. Bumstead, P. A. Midgley, P. A. Chater, D. A. Keen, T. D. Bennett, Synthesis and Properties of a Compositional Series of MIL-53(Al) Metal–Organic Framework Crystal-Glass Composites, *J. Am. Chem. Soc.*, **2019**, 141, 15641-15648.

32. T. Ogawa, K. Takahashi, S. S. Nagarkar, K. Ohara, Y.-l. Hong, Y. Nishiyama, S. Horike, Coordination polymer glass from a protic ionic liquid: proton conductivity and mechanical properties as an electrolyte, *Chem. Sci.*, **2020**, 11, 5175-5181.

33. V. Nozari, C. Calahoo, J. M. Tuffnell, D. A. Keen, T. D. Bennett, L. Wondraczek, Ionic liquid facilitated melting of the metal-organic framework ZIF-8. *Nat. Commun.,* **2021**, 12, 5703.

34. F. Cao, S. S. Sørensen, A. K. Christensen, S. Mollick, X. Ge, D. Sun, A. B. Nielsen, N. C. Nielsen, N. Lock, R. Lu, R*.* Klemmt, Continuous structure modification of metal-organic framework glasses via halide salts, **2025**. DOI: 10.26434/chemrxiv-2024-bgf8b-v2.

35. M. Kim, H.-S. Lee, D.-H. Seo, S. J. Cho, E.-c. Jeon, H. R. Moon, Melt-quenched carboxylate metal-organic framework glasses, *Nat. Commun.*, **2024**, 15, 1174.

36. Z. Zhang, Y. Zhao, Transparent and high-porosity aluminum alkoxide network-forming glasses. *Nat. Commun.* **2024**, 15, 7339.

37. Y.-S. Wei, Z. Fan, C. Luo, S. Horike, Desolvation of metal complexes to construct metal–organic framework glasses. *Nat. Synth.,* **2024**, 3, 214-223.

38. H. Yoshino, K. Yamagami, H. Wadati, H. Yamagishi, H. Setoyama, S. Shimoda, A. Mishima, B. Le Ouay, R. Ohtani, M. Ohba, Coordination Geometry Changes in Amorphous Cyanide-Bridged Metal–Organic Frameworks upon Water Adsorption, *Inorg. Chem.*, **2021**, 60, 3338-3344.

39. Z. Yu, Z. Gu, J. Lei, G. Zheng, Vacuum treated amorphous MOF mixed matrix membrane for methane/nitrogen separation, *J. Solid State Chem.*, **2023**, 320, 123852.

40. A. J. Graham, J.-C. Tan, D. R. Allan, S. A. Moggach, The effect of pressure on Cu-btc: framework compression vs. guest inclusion, *Chem. Commun*., **2012**, 48, 1535-1537.

41. B. Yeskendir, J.-P. Dacquin, Y. Lorgouilloux, C. Courtois, S. Royer, J. Dhainaut, From metal–organic framework powders to shaped solids: recent developments and challenges, *Mater. Adv.*, **2021**, 2, 7139-7186.

42. T. D. Bennett, P. J. Saines, D. A. Keen, J. C. Tan, A. K. Cheetham, Ball‐milling‐induced amorphization of zeolitic imidazolate frameworks (ZIFs) for the irreversible trapping of iodine, *Chem. Eur. J.*, **2013**, 19, 7049-7055.

43. A. Bhattacharjee, M. K. Purkait, S. Gumma, Doxorubicin loading capacity of MIL-100 (Fe): effect of synthesis conditions, *J. Inorg. Organomet. Polym. Mater.*, **2020**, 30, 2366-2375.

44. M. Almáši, N. Király, V. Zeleňák, M. Vilková, S. Bourrelly, Zinc(ii) and cadmium(ii) amorphous metal-organic frameworks (aMOFs): study of activation process and high-pressure adsorption of greenhouse gases, *RSC Adv.*, **2021**, 11, 20137-20150.

45. J. Liu, B. Li, V. Martins, Y. Huang, Y. Song, Enhancing CO_2_ Adsorption in MIL-53(Al) through Pressure–Temperature Modulation: Insights from Guest–Host Interactions, *J. Phys. Chem. C.*, **2024**, 128, 8007-8015.

46. E. Beake, M. Dove, A. Phillips, D. Keen, M. Tucker, A. Goodwin, T. Bennett, A. Cheetham, Flexibility of zeolitic imidazolate framework structures studied by neutron total scattering and the reverse Monte Carlo method, *J. Phys.: Condens. Matter.*, **2013**, 25, 395403.

47. Z. Gao, B. Xu, Y. Fan, T. Zhang, S. Chen, S. Yang, W. Zhang, X. Sun, Y. Wei, Z. Wang, Topological‐Distortion‐Driven Amorphous Spherical Metal‐Organic Frameworks for High‐Quality Single‐Mode Microlasers, *Angew. Chem. Int. Ed.*, **2021**, 60, 6362-6366.

48. R. Pallach, J. Keupp, K. Terlinden, L. Frentzel-Beyme, M. Kloß, A. Machalica, J. Kotschy, S. K. Vasa, P. A. Chater, C. Sternemann, M. T. Wharmby, R. Linser, R. Schmid, S. Henke, Frustrated flexibility in metal-organic frameworks, *Nat. Commun.*, **2021**, 12, 4097.

49. J. Fonseca, S. Choi, Flexible amorphous metal–organic frameworks with π Lewis acidic pore surface for selective adsorptive separations, *Dalton Trans.*, **2021**, 50, 3145-3154.

50. M. R. Ryder, T. D. Bennett, C. S. Kelley, M. D. Frogley, G. Cinque, J.-C. Tan, Tracking thermal-induced amorphization of a zeolitic imidazolate framework via synchrotron in situ far-infrared spectroscopy, *Chem. Commun*., **2017**, 53, 7041-7044.

51. K. T. Butler, S. D. Worrall, C. D. Molloy, C. H. Hendon, M. P. Attfield, R. A. Dryfe, A. Walsh, Electronic structure design for nanoporous, electrically conductive zeolitic imidazolate frameworks, *J. Mater. Chem. C.*, **2017**, 5, 7726-7731.

52. A. S. Babal, B. E. Souza, A. F. Möslein, M. Gutiérrez, M. D. Frogley, J.-C. Tan, Broadband Dielectric Behavior of an MIL-100 Metal–Organic Framework as a Function of Structural Amorphization, *ACS Appl. Electron. Mater.*, **2021**, 3, 1191-1198.

53. R. Lin, X. Li, A. Krajnc, Z. Li, M. Li, W. Wang, L. Zhuang, S. Smart, Z. Zhu, D. Appadoo, Mechanochemically synthesised flexible electrodes based on bimetallic metal-organic framework glasses for the oxygen evolution reaction, *Angew. Chem. Int. Ed.*, **2022**, 61, e202112880.

54. J. Hou, P. Chen, A. Shukla, A. Krajnc, T. Wang, X. Li, R. Doasa, L. H. Tizei, B. Chan, D. N. Johnstone, Liquid-phase sintering of lead halide perovskites and metal-organic framework glasses, *Science.*, **2021**, 374, 621-625.

55. J. Hou, M. L. Ríos Gómez, A. Krajnc, A. McCaul, S. Li, A. M. Bumstead, A. F. Sapnik, Z. Deng, R. Lin, P. A. Chater, D. S. Keeble, D. A. Keen, D. Appadoo, B. Chan, V. Chen, G. Mali, T. D. Bennett, Halogenated Metal–Organic Framework Glasses and Liquids, *J. Am. Chem. Soc.*, **2020**, 142, 3880-3890.

56. C. Zhou, L. Longley, A. Krajnc, G. J. Smales, A. Qiao, I. Erucar, C. M. Doherty, A. W. Thornton, A. J. Hill, C. W. Ashling, O. T. Qazvini, S. J. Lee, P. A. Chater, N. J. Terrill, A. J. Smith, Y. Yue, G. Mali, D. A. Keen, S. G. Telfer, T. D. Bennett, Metal-organic framework glasses with permanent accessible porosity, *Nat. Commun.*, **2018**, 9, 5042.

57. Y. Ohara, A. Hinokimoto, W. Chen, T. Kitao, Y. Nishiyama, Y.-l. Hong, S. Kitagawa, S. Horike, Formation of coordination polymer glass by mechanical milling: dependence on metal ions and molecular doping for H^+^ conductivity, *Chem. Commun*., **2018**, 54, 6859-6862.

58. R. S. K. Madsen, A. Qiao, J. Sen, I. Hung, K. Chen, Z. Gan, S. Sen, Y. Yue, Ultrahigh-field ^67^Zn NMR reveals short-range disorder in zeolitic imidazolate framework glasses, *Science.*, 2020, 367, 1473-1476.

59. W. Chen, S. Horike, D. Umeyama, N. Ogiwara, T. Itakura, C. Tassel, Y. Goto, H. Kageyama, S. Kitagawa, Glass Formation of a Coordination Polymer Crystal for Enhanced Proton Conductivity and Material Flexibility, *Angew. Chem. Int. Ed.*, **2016**, 55, 5195-5200.

60. S. Bordiga, F. Bonino, K. P. Lillerud, C. Lamberti, X-ray absorption spectroscopies: useful tools to understand metallorganic frameworks structure and reactivity, *Chem. Soc. Rev.*, **2010**, 39, 4885-4927.

61. A. Qiao, H. Tao, M. P. Carson, S. W. Aldrich, L. M. Thirion, T. D. Bennett, J. C. Mauro, Y. Yue, Optical properties of a melt-quenched metal-organic framework glass, *Opt. Lett.*, **2019**, 44, 1623-1625.

62. T. D. Bennett, J.-C. Tan, Y. Yue, E. Baxter, C. Ducati, N. J. Terrill, H. H.-M. Yeung, Z. Zhou, W. Chen, S. Henke, Hybrid glasses from strong and fragile metal-organic framework liquids, *Nat. Commun.*, **2015**, 6, 8079.

63. L. Longley, S. M. Collins, C. Zhou, G. J. Smales, S. E. Norman, N. J. Brownbill, C. W. Ashling, P. A. Chater, R. Tovey, C.-B. Schönlieb, T. F. Headen, N. J. Terrill, Y. Yue, A. J. Smith, F. Blanc, D. A. Keen, P. A. Midgley, T. D. Bennett, Liquid phase blending of metal-organic frameworks, *Nat. Commun.*, **2018**, 9, 2135.

64. J. Hou, C. W. Ashling, S. M. Collins, A. Krajnc, C. Zhou, L. Longley, D. N. Johnstone, P. A. Chater, S. Li, M.-V. Coulet, P. L. Llewellyn, F.-X. Coudert, D. A. Keen, P. A. Midgley, G. Mali, V. Chen, T. D. Bennett, Metal-organic framework crystal-glass composites, *Nat. Commun.*, **2019**, 10, 2580.

65. S. Li, S. Yu, S. M. Collins, D. N. Johnstone, C. W. Ashling, A. F. Sapnik, P. A. Chater, D. S. Keeble, L. N. McHugh, P. A. Midgley, D. A. Keen, T. D. Bennett, A new route to porous metal–organic framework crystal–glass composites, *Chem. Sci.*, **2020**, 11, 9910-9918.

66. T. D. Bennett, D. A. Keen, J.-C. Tan, E. R. Barney, A. L. Goodwin, A. K. Cheetham, Thermal Amorphization of Zeolitic Imidazolate Frameworks, *Angew. Chem. Int. Ed.*, **2011**, 50, 3067-3071.

67. A. F. Sapnik, I. Bechis, A. M. Bumstead, T. Johnson, P. A. Chater, D. A. Keen, K. E. Jelfs, T. D. Bennett, Multivariate analysis of disorder in metal–organic frameworks, *Nat. Commun.*, **2022**, 13, 2173.

68. J. E. M. Laulainen, D. N. Johnstone, I. Bogachev, L. Longley, C. Calahoo, L. Wondraczek, D. A. Keen, T. D. Bennett, S. M. Collins, P. A. Midgley, Mapping short-range order at the nanoscale in metal–organic framework and inorganic glass composites, *Nanoscale.*, **2022**, 14, 16524-16535.

69. R. Lin, J. Hou, M. Li, Z. Wang, L. Ge, S. Li, S. Smart, Z. Zhu, T. D. Bennett, V. Chen, Interfacial engineering of a polymer–MOF composite by in situ vitrification, *Chem. Commun.*, **2020**, 56, 3609-3612.

70. L. Liu, D. Zhang, Y. Zhu, Y. Han, Bulk and local structures of metal–organic frameworks unravelled by high-resolution electron microscopy, *Commun. Chem.*, **2020**, 3, 99.

71. L. Frentzel-Beyme, P. Kolodzeiski, J.-B. Weiß, A. Schneemann, S. Henke, Quantification of gas-accessible microporosity in metal-organic framework glasses, *Nat. Commun.*, **2022**, 13, 7750.

72. A. W. Thornton, K. E. Jelfs, K. Konstas, C. M. Doherty, A. J. Hill, A. K. Cheetham, T. D. Bennett, Porosity in metal–organic framework glasses. *Chem. Commun.,* **2016**, 52, 3750-3753.

73. S. Li, R. Limbach, L. Longley, A. A. Shirzadi, J. C. Walmsley, D. N. Johnstone, P. A. Midgley, L. Wondraczek, T. D. Bennett, Mechanical Properties and Processing Techniques of Bulk Metal–Organic Framework Glasses, *J. Am. Chem. Soc.*, **2019**, 141, 1027-1034.

74. T. To, S. S. Sørensen, M. Stepniewska, A. Qiao, L. R. Jensen, M. Bauchy, Y. Yue, M. M. Smedskjaer, Fracture toughness of a metal–organic framework glass, *Nat. Commun.*, **2020**, 11, 2593.

75. A. Qiao, T. To, M. Stepniewska, H. Tao, L. Calvez, X. Zhang, M. M. Smedskjaer, Y. Yue, Deformation mechanism of a metal–organic framework glass under indentation, *Phys. Chem. Chem. Phys.*, **2021**, 23, 16923-16931.

76. M. Stepniewska, K. Januchta, C. Zhou, A. Qiao, M. M. Smedskjaer, Y. Yue, Observation of indentation-induced shear bands in a metal-organic framework glass, *Proc. Natl. Acad. Sci.*, **2020**, 117, 10149-10154.

77. Q. Zheng, Y. Zhang, M. Montazerian, O. Gulbiten, J. C. Mauro, E. D. Zanotto, Y. Yue, Understanding Glass through Differential Scanning Calorimetry, *Chem. Rev.*, **2019**, 119, 7848-7939.

78. A. M. Bumstead, M. F. Thorne, T. D. Bennett, Identifying the liquid and glassy states of coordination polymers and metal–organic frameworks, *Faraday Discuss.*, **2021**, 225, 210-225.

79. L. Longley, C. Calahoo, R. Limbach, Y. Xia, J. M. Tuffnell, A. F. Sapnik, M. F. Thorne, D. S. Keeble, D. A. Keen, L. Wondraczek, T. D. Bennett, Metal-organic framework and inorganic glass composites, *Nat. Commun.*, **2020**, 11, 5800.

80. Y. Wang, H. Jin, Q. Ma, K. Mo, H. Mao, A. Feldhoff, X. Cao, Y. Li, F. Pan, Z. Jiang, A MOF Glass Membrane for Gas Separation, *Angew Chem. Int. Ed.*, **2020**, 59, 4365-4369.

81. C. Liu, J. Wang, J. Wan, Y. Cheng, R. Huang, C. Zhang, W. Hu, G. Wei, C. Yu, Amorphous Metal–Organic Framework‐Dominated Nanocomposites with Both Compositional and Structural Heterogeneity for Oxygen Evolution, *Angew. Chem. Int. Ed.*, **2020**, 59, 3630-3637.

82. Y. Sun, Y. Liu, Design of metal-organic framework membranes towards ultimate gas separation, *Green Chem. Eng.*, **2021**, 2, 14-16.

83. G. Jiang, C. Qu, F. Xu, E. Zhang, Q. Lu, X. Cai, S. Hausdorf, H. Wang, S. Kaskel, Glassy metal–organic‐framework‐based quasi‐solid‐state electrolyte for high‐performance lithium‐metal batteries, *Adv. Funct. Mater.*, **2021**, 31, 2104300.

84. M. Mubashir, L. F. Dumée, Y. Y. Fong, N. Jusoh, J. Lukose, W. S. Chai, P. L. Show, Cellulose acetate-based membranes by interfacial engineering and integration of ZIF-62 glass nanoparticles for CO_2_ separation, *J. Hazard Mater.*, **2021**, 415, 125639.

85. Q. Yang, N. Zhang, Q. Zhang, J.-Y. Zhang, Y.-Z. Fang, M. Zhou, Band bending induced charge redistribution on the amorphous MIL-53(Al)/Co-LDH conjunction to boost the supercapacitive and oxygen evolution performance, *Electrochim. Acta.*, **2022**, 429, 141057.

86. X. Liu, X. Zhao, H. Meng, J. Jin, Dual MOFs composites: MIL-53 coated with amorphous UiO-66 for enhanced photocatalytic oxidation of tetracycline and methylene blue, *Nano Res.*, **2023**, 16, 6160-6166.

87. C. W. Ashling, L. K. Macreadie, T. J. Southern, Y. Zhang, L. N. McHugh, R. C. Evans, S. Kaskel, S. G. Telfer, T. D. Bennett, Guest size limitation in metal–organic framework crystal–glass composites, *J. Mater. Chem. A.*, **2021**, 9, 8386-8393.

88. L. Longley, S. M. Collins, S. Li, G. J. Smales, I. Erucar, A. Qiao, J. Hou, C. M. Doherty, A. W. Thornton, A. J. Hill, Flux melting of metal–organic frameworks, *Chem. Sci.*, **2019**, 10, 3592-3601.

89. Y. Zhang, Y. Wang, H. Xia, P. Gao, Y. Cao, H. Jin, Y. Li, A hybrid ZIF-8/ZIF-62 glass membrane for gas separation, *Chem. Commun.*, **2022**, 58, 9548-9551.

90. H. Xia, H. Jin, Y. Zhang, H. Song, J. Hu, Y. Huang, Y. Li, A long-lasting TIF-4 MOF glass membrane for selective CO_2_ separation, *J. Membr. Sci.*, **2022**, 655, 120611.

91. J. Li, J. Wang, Q. Li, M. Zhang, J. Li, C. Sun, S. Yuan, X. Feng, B. Wang, Coordination Polymer Glasses with Lava and Healing Ability for High‐Performance Gas Sieving, *Angew. Chem.*, **2021**, 133, 21474-21479.

92. M. A. Ali, X. Liu, H.-T. Sun, J. Ren, J. Qiu, Metal Inorganic–Organic Complex Glass and Fiber for Photonic Applications, *Chem. Mater.*, **2022**, 34, 2476-2483.

93. N. K. Kulachenkov, S. Bruyere, S. A. Sapchenko, Y. A. Mezenov, D. Sun, A. A. Krasilin, A. Nominé, J. Ghanbaja, T. Belmonte, V. P. Fedin, Ultrafast Melting of Metal–Organic Frameworks for Advanced Nanophotonics, *Adv. Funct. Mater.*, **2020**, 30, 1908292.

94. X. Miao, P. Wang, R. Sun, J. Li, Z. Wang, T. Zhang, R. Wang, Z. Li, Y. Bai, R. Hao, Liquid metal‐organic frameworks in‐situ derived interlayer for high‐performance solid‐state Na‐metal batteries, *Adv. Energy Mater.*, **2021**, 11, 2102396.

95. C. Thanaphatkosol, N. Ma, K. Kageyama, T. Watcharatpong, T. Tiyawarakul, K. Kongpatpanich, S. Horike, Modulation of proton conductivity in coordination polymer mixed glasses, *Chem. Commun.*, **2022**, 58, 6064-6067.

96. T. Ogawa, K. Takahashi, T. Kurihara, S. S. Nagarkar, K. Ohara, Y. Nishiyama, S. Horike, Network Size Control in Coordination Polymer Glasses and Its Impact on Viscosity and H^+^ Conductivity, *Chem. Mater.*, **2022** , 34, 5832-5841.

97. N. Ma, S. Kosasang, A. Yoshida, S. Horike, Proton-conductive coordination polymer glass for solid-state anhydrous proton batteries, *Chem. Sci.*, **2021**, 12, 5818-5824.

98. C. Gao, Z. Jiang, S. Qi, P. Wang, L. R. Jensen, M. Johansen, C. K. Christensen, Y. Zhang, D. B. Ravnsbæk, Y. Yue, Metal‐organic framework glass anode with an exceptional cycling‐induced capacity enhancement for lithium‐ion batteries, *Adv. Mater.*, **2022**, 34, 2110048.

99. R. Lin, Y. Yao, M. Y. B. Zulkifli, X. Li, S. Gao, W. Huang, S. Smart, M. Lyu, L. Wang, V. Chen, Binder-free mechanochemical metal–organic framework nanocrystal coatings, *Nanoscale.*, **2022**, 14, 2221-2229.

100. Z. Yang, D. Ao, X. Guo, L. Nie, Z. Qiao, C. Zhong, Preparation and characterization of small-size amorphous MOF mixed matrix membrane, *Sep. Purif. Technol.*, **2021**, 272, 118860.

101. H. Mahdavi, J. F. Olorunyomi, N. T. Eden, C. M. Doherty, D. Acharya, S. J. D. Smith, X. Mulet, M. R. Hill, Design and Development of a Self-Supporting ZIF-62 Glass MOF Membrane with Enhanced Molecular Sieving for High H_2_ Separation Efficiency. *ACS Omega,* **2025**, 10, 7441-7451.

102. X. Jin, G.-Q. Wang, D. Ma, S.-Q. Deng, S.-L. Cai, J. Fan, W.-G. Zhang, S.-R. Zheng, Cationic Amorphous Metal–Organic Cage-Based Materials for the Removal of Oxo-Anions from Wate, *ACS Appl. Nano Mater.*, **2019**, 2, 5824-5832.

103. K. W. Chapman, D. F. Sava, G. J. Halder, P. J. Chupas, T. M. Nenoff, Trapping guests within a nanoporous metal–organic framework through pressure-induced amorphization, *J. Am. Chem. Soc.*, **2011**, 133, 18583-18585.

104. S. Horike, W. Chen, T. Itakura, M. Inukai, D. Umeyama, H. Asakura, S. Kitagawa, Order-to-disorder structural transformation of a coordination polymer and its influence on proton conduction, *Chem. Commun.*, **2014**, 50, 10241-10243.

105. S. S. Nagarkar, S. Horike, T. Itakura, B. Le Ouay, A. Demessence, M. Tsujimoto, S. Kitagawa, Enhanced and optically switchable proton conductivity in a melting coordination polymer crystal, *Angew. Chem. Int. Ed.*, **2017**, 129, 5058-5063.

106. F. Yang, W. Li, B. Tang, Facile synthesis of amorphous UiO-66 (Zr-MOF) for supercapacitor application, *J. Alloys Compd.*, **2018**, 733, 8-14.

107. X. Zhang, G. Li, Y. Zhang, D. Luo, A. Yu, X. Wang, Z. Chen, Amorphizing metal-organic framework towards multifunctional polysulfide barrier for high-performance lithium-sulfur batteries, *Nano Energy.*, **2021**, 86, 106094.

108. H. Zhang, H. Mei, D. Qin, Z. Li, Z. Hou, X. Lu, B. Xu, D. Sun, Conversion of Amorphous MOF Microspheres into a Nickel Phosphate Battery-Type Electrode Using the “Anticollapse” Two-Step Strategy, *Inorg. Chem.*, **2021**, 60, 17094-17102.

109. C. Lavenn, L. Okhrimenko, N. Guillou, M. Monge, G. Ledoux, C. Dujardin, R. Chiriac, A. Fateeva, A. Demessence, A luminescent double helical gold (I)–thiophenolate coordination polymer obtained by hydrothermal synthesis or by thermal solid-state amorphous-to-crystalline isomerization, *J. Mater. Chem. C.*, **2015**, 3, 4115-4125.

110. J.-W. Xiu, G.-E. Wang, M.-S. Yao, C.-C. Yang, C.-H. Lin, G. Xu, Electrical bistability in a metal–organic framework modulated by reversible crystalline-to-amorphous transformations, *Chem. Commun.*, **2017**, 53, 2479-2482.

111. C. Orellana-Tavra, M. Köppen, A. Li, N. Stock, D. Fairen-Jimenez, Biocompatible, crystalline, and amorphous bismuth-based metal-organic frameworks for drug delivery. *ACS Appl. Mater. Interfaces,* **2020**, 12, 5633-5641.

112. B. Zhou, Z. Qi, D. Yan, Highly Efficient and Direct Ultralong All‐Phosphorescence from Metal–Organic Framework Photonic Glasses, *Angew. Chem. Int. Ed*., **2022**, 61, e202208735.

113. M. A. Ali, X. Liu, Y. Li, J. Ren, J. Qiu, Nonlinear-Optical Response in Zeolitic Imidazolate Framework Glass, *Inorg. Chem.*, **2020**, 59, 8380-8386.

114. J. Dang, R. Zhu, W. Fang, Y. Hu, Y. Wu, S. Xin, M. Li, B. Chen, H. Zhao, Z. Li, The construction of a ratiometric dual-emitting amorphous europium-organic frameworks for sensitive detection of water in white spirit, *Dyes Pigm.*, **2022**, 206, 110602.
